# Two paralogous PHD finger proteins participate in natural genome editing in *Paramecium tetraurelia*

**DOI:** 10.1242/jcs.261979

**Published:** 2024-08-30

**Authors:** Lilia Häußermann, Aditi Singh, Estienne C. Swart

**Affiliations:** Max Planck Institute for Biology, Max-Planck-Ring 5, 72076 Tübingen, Germany

**Keywords:** Genome reorganization, PHD finger proteins, Small RNAs, *Paramecium tetraurelia*

## Abstract

The unicellular eukaryote *Paramecium tetraurelia* contains functionally distinct nuclei: germline micronuclei (MICs) and a somatic macronucleus (MAC). During sex, the MIC genome is reorganized into a new MAC genome and the old MAC is lost. Almost 45,000 unique internal eliminated sequences (IESs) distributed throughout the genome require precise excision to guarantee a functional new MAC genome. Here, we characterize a pair of paralogous PHD finger proteins involved in DNA elimination. DevPF1, the early-expressed paralog, is present in only some of the gametic and post-zygotic nuclei during meiosis. Both DevPF1 and DevPF2 localize in the new developing MACs, where IES excision occurs. Upon *DevPF2* knockdown (KD), long IESs are preferentially retained and late-expressed small RNAs decrease; no length preference for retained IESs was observed in *DevPF1*-KD and development-specific small RNAs were abolished. The expression of at least two genes from the new MAC with roles in genome reorganization seems to be influenced by *DevPF1-* and *DevPF2*-KD. Thus, both PHD fingers are crucial for new MAC genome development, with distinct functions, potentially via regulation of non-coding and coding transcription in the MICs and new MACs.

## Teaser

**Summary:** Investigation of the PHD finger proteins DevPF1 and DevPF2 indicates that they play crucial roles in genome reorganization in the ciliate *Paramecium*, potentially by regulating the coding and non-coding transcription during development.

## INTRODUCTION

A unique feature shared by all ciliates is the presence of nuclear dimorphism. In *Paramecium tetraurelia* (henceforth *Paramecium*) the two micronuclei (MICs) resemble the germline of multicellular organisms, being transcriptionally silent throughout most of the life cycle and generating haploid nuclei during meiosis that develop and give rise to all nuclei in the subsequent generation. Also similar to the multicellular soma, the macronucleus (MAC) is optimized for most gene expression, and originates from a MIC copy. The old MAC is fragmented during sexual division and subsequently diluted across cell divisions, with the new MAC completely taking over somatic expression. The development from the MIC genome into the MAC genome in *Paramecium* is a natural form of genome editing that requires extensive reorganization, including genome amplification (∼800n), chromosome fragmentation and the elimination of ∼25% of the sequence content (Arnaiz et al., 2012; Guérin et al., 2017). These MIC genome-specific sequences comprise repeats, transposable elements and internal eliminated sequences (IESs).

In contrast to other elimination events, IES elimination requires precise excision in *Paramecium*. Precise IES excision is not characteristic of all ciliates. Notably, in *Tetrahymena*, an oligohymenophorean relative of *Paramecium*, IESs are predominantly imprecisely excised and mostly tolerated in intergenic regions (Hamilton et al., 2016). The ∼45,000 IESs in *Paramecium* are scattered throughout the genome in both non-coding and coding regions and vary from tens to thousands of base pairs in length (Arnaiz et al., 2012). The coding density of the *Paramecium* MAC genome is high and there is only a weak bias towards IESs being located in intergenic regions, so most IESs are intragenic (Arnaiz et al., 2012). *Paramecium* IESs are flanked by conserved 5′-TA-3′ dinucleotides (Klobutcher and Herrick, 1995) and excised by PiggyMAC (Pgm). Pgm is a domesticated transposase derived from PiggyBac transposases (Baudry et al., 2009), like the excisase responsible for IES excision in *Tetrahymena* (Cheng et al., 2010). The weakly conserved ∼5 bp long inverted repeats at *Paramecium* IES ends (Klobutcher and Herrick, 1995) fail to provide enough specificity for reliable Pgm recruitment (Arnaiz et al., 2012). This suggests that other factors are needed for precise IES targeting.

The targeting of MIC-specific sequences for elimination is thought to be assisted by small non-coding RNAs, first characterized in *Tetrahymena* (Chalker and Yao, 2001; Mochizuki et al., 2002). Like *Tetrahymena*, the biogenesis of the 25-nucleotide (nt) scan RNAs (scnRNAs) occurs during meiosis in the *Paramecium* MICs. Bidirectional non-coding transcription of the MIC genome is thought to be initiated by the putative transcription elongation factor Spt5m (Gruchota et al., 2017) and followed by the cleavage of long double-stranded RNA (dsRNA) by the closely related Dicer-like protein paralogs Dcl2 and Dcl3 (Hoehener et al., 2018; Lepère et al., 2009; Sandoval et al., 2014). Together the Argonaute/Piwi proteins Ptiwi01 and Ptiwi09 (collectively denoted Ptiwi01/09; also close paralogs) process the resulting short dsRNAs, removing one of the two strands, and stabilize single-stranded scnRNAs throughout the selection process in the parental MAC and targeting of MIC-specific sequences in the new MACs (Bouhouche et al., 2011; Furrer et al., 2017). In the parental MAC, Gtsf1 has recently been proposed to promote ubiquitination and subsequent degradation of the Ptiwi01/09 complexes harboring MAC-matching scnRNAs (Charmant et al., 2023 preprint; Wang et al., 2023 preprint). In the new MACs, the putative transcription elongation factor TFIIS4 is proposed to promote non-coding transcription, which is required for scanning the developing genome (Maliszewska-Olejniczak et al., 2015).

In *Tetrahymena*, H3K9 and H3K27 methylation precede IES excision (Liu et al., 2007; Taverna et al., 2002), and it has been shown in *Paramecium* that development-specific H3K9me3 and H3K27me3 histone mark (me3, trimethylation) deposition by the PRC2 complex depends on scnRNAs and is essential for the elimination of transposons and IESs (Frapporti et al., 2019; Ignarski et al., 2014; Lhuillier-Akakpo et al., 2014; Miró-Pina et al., 2022; Wang et al., 2022). We have recently shown that the ISWI1 chromatin remodeling complex is necessary for IES excision precision, and Ptiwi01/09 co-immunoprecipitate together with ISWI1 in a crosslinked treatment (Singh et al., 2022, 2023 preprint). After the initial onset of IES excision, additional single-stranded sRNAs, iesRNAs, ranging in size from ∼26 to 30 bp, are produced by Dcl5 from excised IES fragments and further processed and transported by Ptiwi10 and Ptiwi11 (collectively Ptiwi10/11) (Furrer et al., 2017; Sandoval et al., 2014). iesRNAs have been proposed to participate in a positive feedback loop for the efficient removal of all IES copies (Sandoval et al., 2014). Nevertheless, only a fraction of IES excision appears to depend on scnRNAs or iesRNAs (Nowacki et al., 2005; Sandoval et al., 2014).

Despite the knowledge gained in the past decades, the picture of IES excision is far from complete. To identify novel genes involved in IES excision, we examined proteins potentially associated with ISWI1, a chromatin remodeler that we have recently shown facilitates precise IES excision (Singh et al., 2022).

## RESULTS

### Identification of a novel protein involved in IES excision

Recently, we reported evidence supporting the formation of a protein complex involving ISWI1 and the ICOP proteins (Singh et al., 2023 preprint). We conducted an RNAi screen of additional genes that were unique in the ISWI1 co-immunoprecipitation (IP) mass spectrometry (MS) data and exhibited upregulation in a developmental gene expression time course from ParameciumDB (Arnaiz et al., 2017) (Fig. S1A).

In the screening, we sought phenotypic evidence for failed genome reorganization in the form of growth defects (assessed by survival tests), and substantial IES retention (assessed by IES retention PCRs). *ND7*, a gene involved in trichocyst discharge (Lefort-Tran et al., 1981), was used as a negative control as its silencing does not impair genome reorganization (Nowacki et al., 2005). NOWA1-KD, which affects the excision of scnRNA-dependent IESs (Nowacki et al., 2005), was used as a positive control. Candidate 2 (PTET.51.1.G0620188) displayed both IES retention and lethality in the new progeny, whereas candidate 1 (PTET.51.1.G0990120) showed high lethality without IES retention (Fig. S1B,C). Therefore, candidate 2 was selected for further investigation.

### DevPF2 and DevPF1 are paralogous PHD finger proteins

**Fig. 1.**
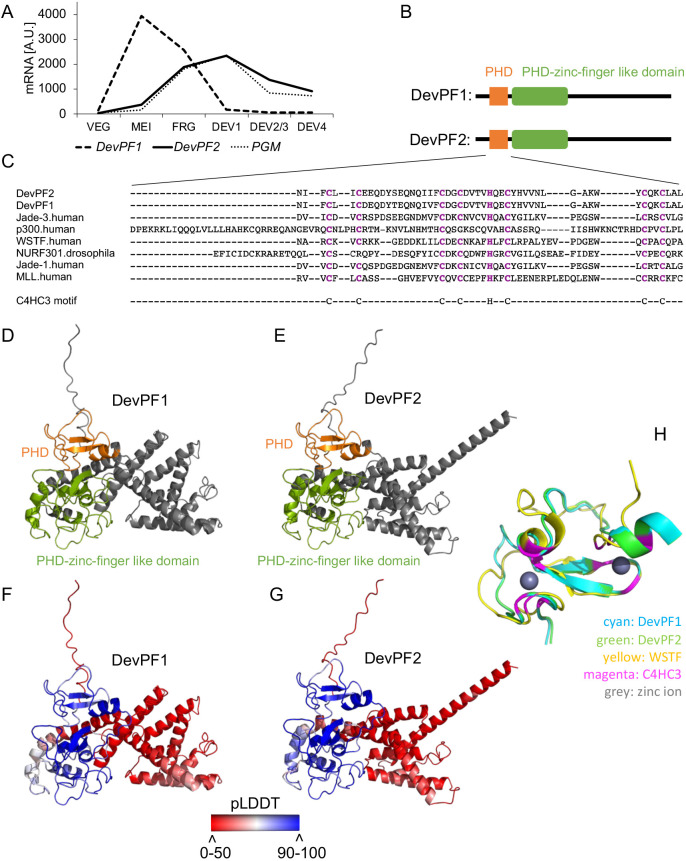
**Features of the PHD finger proteins DevPF1 and DevPF2.** (A) mRNA expression profiles for *DevPF1*, *DevPF2* and *PGM* during various developmental stages: VEG (vegetative growth), MEI (micronuclear meiosis and macronuclear fragmentation), FRG (∼50% of the population with fragmented maternal MACs), DEV1 (significant proportion with visible anlagen), DEV2/3 (majority of cells have visible anlagen), DEV4 (majority of cells have visible anlagen). Expression data retrieved from ParameciumDB (Arnaiz et al., 2017). (B) Schematic representation of predicted domain architecture for DevPF1 and DevPF2. (C) Multiple sequence alignment (Clustal Omega) of DevPF1 and DevPF2 amino acid sequence with PHD domains of published human and *Drosophila* PHD finger proteins. (D–F) Predicted protein structures (AlphaFold2) for DevPF1 and DevPF2, colored by domain (PHD, orange; PHD-zinc-finger-like domain, green) (D,E) and by prediction confidence (pLDDT: predicted local distance difference test) (F,G). (H) Structure predictions of DevPF1 and DevPF2 PHD domain superimposed with NMR structure of the WSTF PHD domain (PDB 1F62).

The *Paramecium aurelia* species complex, to which *P. tetraurelia* belongs, has undergone multiple whole-genome duplications, with many closely related paralogs generated from the most recent of these (Sellis et al., 2021). The chosen candidate has a closely related paralog (PTET.51.1.G0240213) with which it shares 86.6% identity at both the nucleotide and amino acid levels. The paralog is upregulated during sexual development as well, although earlier (Fig. 1A). HMMER3 searches of the Pfam database (Finn et al., 2003) predicted two domains in both proteins: a PHD and a PHD zinc-finger-like domain (Pfam ID: PF13831 and PF13832, respectively; Fig. 1B,D,E). Given that they show development-specific upregulation and (Fig. 1A), we named the paralogs development-specific PHD finger 1 (DevPF1; early-expressed paralog) and 2 (DevPF2; late-expressed paralog).

The highly conserved PHD domain has often been reported to mediate the interaction of nuclear proteins with histone modifications (Sanchez and Zhou, 2011), but other binding affinities have also been described (see Discussion). PHD domains possess a well-conserved motif consisting of eight cysteine and histidine residues (C4HC3) that coordinate two zinc ions, thereby providing it with structural stability. The presence of the C4HC3 motif in both paralogs was confirmed using a multiple sequence alignment with PHD domains from well-established PHD finger proteins from *Homo sapiens* and *Drosophila melanogaster* (Fig. 1C).

AlphaFold2 predicted the structures of the DevPFs with high confidence for the domains (Fig. 1F,G). We compared the PHD predictions with the published structure of the Williams syndrome transcription factor (WSTF; also known as BAZ1B) PHD finger (Pascual et al., 2000). WSTF, which is associated with the Williams syndrome (Lu et al., 1998), is a subunit of the ISWI-containing chromatin remodeling complex WICH (Bozhenok et al., 2002). The superimposition confirmed the orientation of the eight C4HC3 residues in the DevPFs towards the two zinc ions (Fig. 1H), supporting the idea that both paralogs function as PHD finger proteins.

The PiggyMac (Pgm) and PiggyMac-like (Pgml) proteins of *Paramecium* have PHD-like cross-brace zinc fingers (Morellet et al., 2018). In *Tetrahymena*, Lia5, a PiggyMac homolog required for genome rearrangement, was originally identified as a ‘PHD type’ zinc finger protein, then subsequently revised as having a zinc ribbon domain annotation (Shieh and Chalker, 2013). Furthermore, the cross-brace zinc finger domains of Pgm and Pgmls align well to the corresponding Lia5 zinc ribbon domain (Guérineau et al., 2021).

A PHD finger protein called ‘New MAC protein 1’ (Nmp1) in *Tetrahymena* has been reported as being associated with a condensin complex component (Cpd2; Cap-D Two 2) during development (Howard-Till et al., 2019). Nmp1 and the DevPFs are orthologs in OrthoDB v11 (Kuznetsov et al., 2023) (see https://www.orthodb.org/?gene=412030_0:0077fc; the identifier GSPATT00019914001 is the equivalent *P. tetraurelia* strain d4-2 protein of our first paralog and the *Tetrahymena* Nnmp1 identifier is TTHERM_00222270). HA-tagged Nmp1 localizes first in vegetative MACs of *Tetrahymena*, then in its developing new MACs (Howard-Till et al., 2019). However, Nmp1 has not been characterized in detail experimentally and its exact function remains unknown.

### DevPF1 and DevPF2 show distinct nuclear localization

**Fig. 2.**
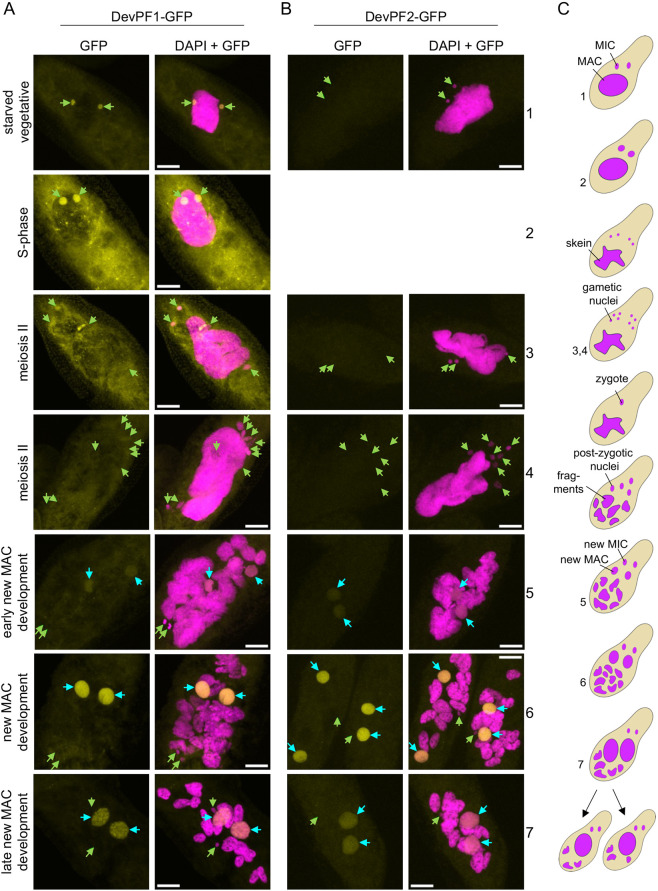
**Subcellular localization of DevPF–GFP proteins.** DevPF1–GFP (A) and DevPF2–GFP (B) localization at various developmental stages. DNA (stained with DAPI) in magenta. GFP signal in yellow. No image of DevPF2–GFP during S-phase was acquired. Green arrows, MICs; cyan arrows, new MAC. Maximum intensity projections of multiple *z*-planes. Scale bars: 10 µm. (C) Schematic overview of nuclear morphology during sexual development, with corresponding cell stages in the images indicated by numbers. Images in A and B are representative of ∼3–5 cells examined for each stage.

**Fig. 3.**
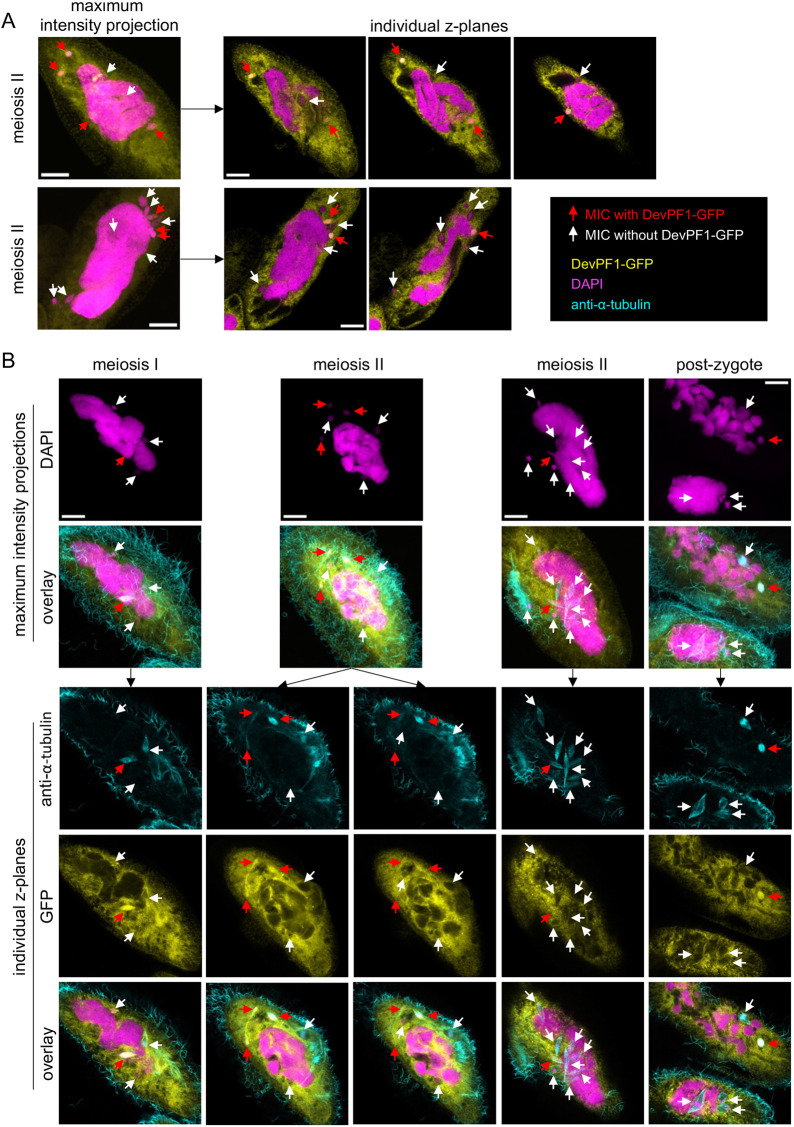
**Selective DevPF1-GFP localization in *Paramecium* MICs.** (A) Overlay of DAPI (DNA stain; pink) and GFP (yellow) signal in two DevPF1–GFP-injected *Paramecium* cells during meiotic stages. Maximum intensity projections (left) and individual *z*-planes of the same stack (right). The maximum-intensity projection images are the same as those shown for meiosis II in Fig. 2A. (B) DevPF1–GFP localization with visualization of nuclear spindle. DAPI (pink), GFP (yellow) and anti-α-tubulin staining (cyan). Maximum intensity projections (top) for DAPI and overlay (DAPI, GFP and anti-α-tubulin). Individual *z*-planes of the same stacks (bottom) for anti-α-tubulin, GFP and overlay. In A and B, red arrows, MICs with DevPF1–GFP localization; white arrows, MICs without DevPF1–GFP localization. Scale bars: 10 µm. Images are representative of 10 repeats.

To determine the localization of both paralogs, we injected DNA constructs encoding DevPF1 and 2 C-terminally tagged with GFP into MACs of vegetative paramecia. The cells were collected during *Paramecium* sexual development for confocal microscopy. The injected cultures displayed no growth defects compared to non-transformed cells (Fig. S2A). However, we observed variable numbers of gametic MICs (Figs 2, 3) and new MACs (Fig. S2C) in some cells, which has been observed frequently for transgenes (e.g. the Nowa1–GFP fusion; Nowacki et al., 2005).

Consistent with the early peak in mRNA expression of DevPF1 from the developmental time course in the ParameciumDB, DevPF1–GFP was expressed during the onset of sexual development, but not in vegetative cells with food vacuoles containing bacteria (Fig. 2A; Fig. S2B). DevPF1–GFP was distributed throughout the cytoplasm and localized in both MICs before and during the S-phase of meiosis, when these nuclei swell (Fig. 2A). Throughout the subsequent meiotic divisions, DevPF1–GFP localized to only a few of the gametic MICs (Fig. 3A). Its micronuclear localization appeared to be independent of nuclear division as detected by the presence of the spindle apparatus (Fig. 3A,B). During post-zygotic mitotic divisions, DevPF1–GFP was observable in certain post-zygotic nuclei, but not in all of them (Fig. 3B). Later during development, DevPF1–GFP was present in the early new MACs and remained in the new MACs throughout development up to very late stages (Fig. 2A) despite the drop in its mRNA levels (Fig. 1B). During new MAC development, there was also comparatively little cytoplasmic DevPF1–GFP compared to that during meiosis.

Consistent with its mRNA expression profile, DevPF2–GFP emerged after the onset of new MAC development and localized within the new MACs, where it remained up to the late stages (Fig. 2B).

### Silencing constructs partially co-silence both paralogs

**Fig. 4.**
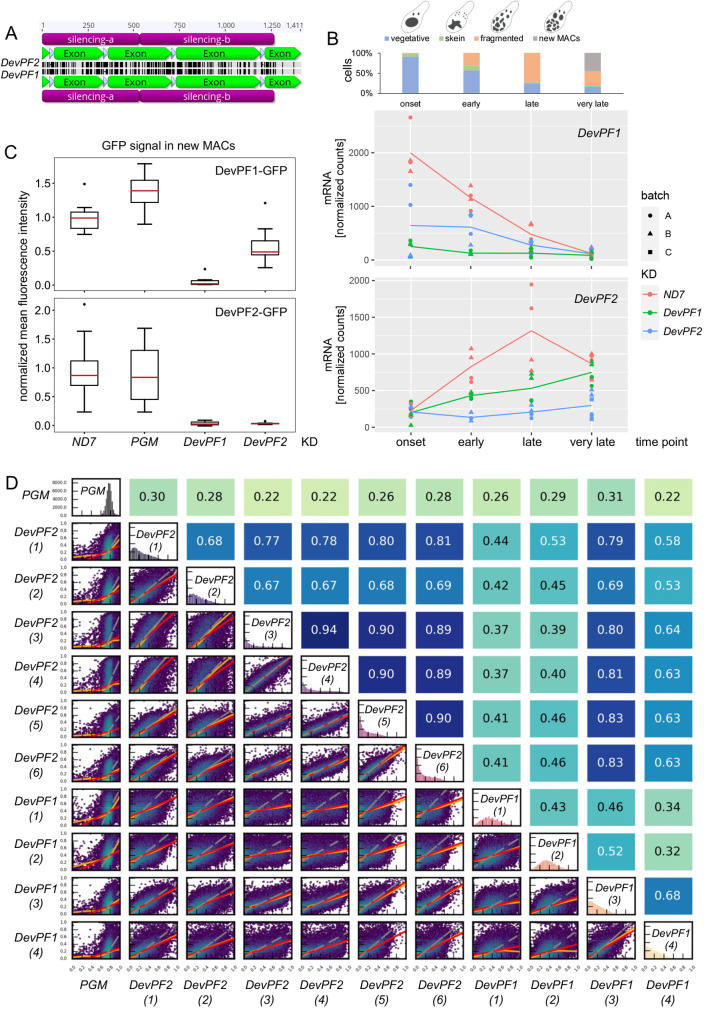
**Co-silencing effects observed in *DevPF* knockdowns.** (A) Nucleotide identity across *DevPF1* (bottom) and *DevPF2* (top) genes. Screenshot of pairwise sequence alignment in Geneious prime software. Silencing region (violet), exon (green), intron (white), perfect identity (gray) and mismatch/gap (black). Scale in base pairs is at the top. (B) mRNA expression levels of *DevPF1* (middle) and *DevPF2* (bottom) upon KDs [*ND7* (control), *DevPF1* and *DevPF2*] at different developmental time points (onset, early, late and very late). Lines represent the mean of all replicates (*n*=4 for all stages except for ‘very late’, where it is 6) for a given KD and time point. The cell stage composition of each time point averaged over all KDs is shown at the top (individual compositions are in Fig. S5), along with schematic representations of the considered cell stages. (C) Protein expression upon KD: fluorescence intensities of DevPF1–GFP (top) and DevPF2–GFP (bottom). The box represents the 25–75th percentiles, and the median is indicated (red line). Whiskers show 1.5 times the interquartile range from the lower or upper quartile. Dots show data points outside the whiskers. Sample size=10. (D) IES retention score (IRS) correlations between *DevPF1*- and *DevPF2*-KD replicates. Diagonal, IRS distributions of individual KDs. Below diagonal, correlation graphs of pairwise comparisons. Above diagonal, corresponding Spearman correlation coefficients. Red lines, ordinary least-squares (OLS) regression; orange lines, locally weighted scatterplot smoothing (LOWESS); gray lines, orthogonal distance regression (ODR).

We used RNAi by feeding to investigate the influence of the DevPFs on IES excision. Two silencing regions were selected (a and b) on each *DevPF1* and *DevPF2* (Fig. 4A). Owing to the lack of regions with sufficient specificity for either of the paralogs, co-silencing was predicted (see Materials and Methods). Hence, we first experimentally verified the possibility of co-silencing with mRNA and protein levels using silencing region a, given that it exhibited less off-target hits.

The mRNA levels of *DevPF1* and *DevPF2* were examined during a time course experiment (more details and further analysis follow below) (Fig. 4B). Consistent with the published expression profiles (Arnaiz et al., 2017), *DevPF1* expression in the *ND7* control knockdown (KD) cells was highest during onset of development and gradually declined to almost no expression at the ‘very late’ time point. The late-expressed *DevPF2* peaked at the ‘late’ time point in the control KD. The expression of both genes was strongly reduced upon their respective KDs (*DevPF1* mRNA levels were reduced upon *DevPF1*-KD; *DevPF2* mRNA levels were reduced upon *DevPF2*-KD). A lesser reduction was also observed upon silencing of the respective paralog (*DevPF1* levels were reduced in *DevPF2*-KD and vice versa). Thus, the *DevPF1* and *DevPF2* silencing constructs led to co-silencing that was less efficient than the target gene silencing.

To investigate how the changes in mRNA levels affect protein levels, we checked the localization of the GFP-tagged DevPFs upon KDs. As *DevPF1* is expressed throughout development, multiple developmental time points were assessed (Fig. S3A). For the late-expressed *DevPF2*, only cell stages with clearly visible new MACs were considered (Fig. S3B). In addition to *ND7*-KD, the knockdown of *PGM*, the gene encoding the PiggyMac IES excisase (Baudry et al., 2009), was performed to test whether the disturbance of IES excision alters DevPF localization. Neither the localization of DevPF1–GFP nor of DevPF2–GFP was impaired by either of the control KDs. In contrast, the GFP signals were almost completely lost upon *DevPF1*- or *DevPF2*-KD. To quantify this observation, GFP fluorescence signals were measured in new MACs (Fig. 4C) as both paralogs exclusively localize to the new MACs during late stages. In line with the observed reduction in mRNA levels, DevPF1–GFP expression was efficiently reduced upon *DevPF1*-KD, whereas *DevPF2*-KD led to a weaker reduction. For DevPF2–GFP, the levels were almost equally reduced in *DevPF1*- and *DevPF2*-KD. Thus, we confirmed co-silencing on both mRNA and protein levels with reduced silencing efficiency compared to the targeted KD. Therefore, all results obtained in KD experiments must be considered, at least in part, as a combined effect of silencing both DevPF1 and DevPF2, albeit with only a partial contribution from the non-targeted gene silencing.

To further investigate the impact of co-silencing on the KD analysis we examined IES retention score (IRS) correlations of multiple KD replicates (more details and further analysis are below). The *DevPF2*-KD replicates showed high to moderate correlation among each other whereas they correlated less well with two out of four *DevPF1*-KD replicates (Fig. 4D). This suggests that despite the partial co-silencing, individual KD effects might be observed.

### *DevPF1* and *DevPF2* affect IES excision genome wide

**Fig. 5.**
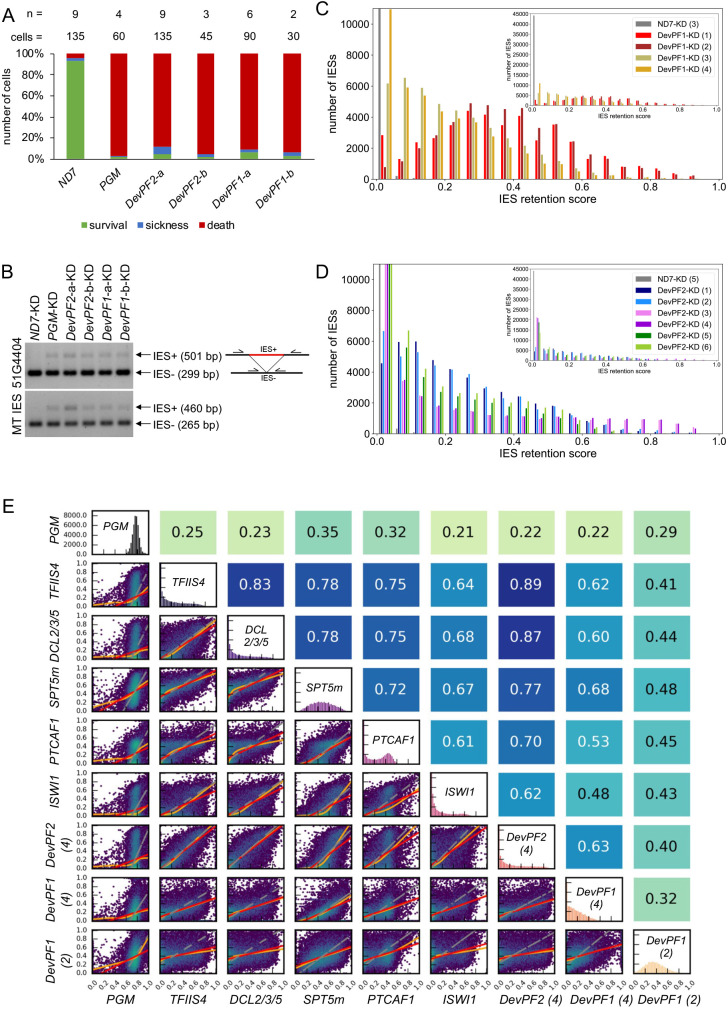
**Effects of *DevPF* knockdowns on genome-wide IES retention.** (A) Viability of new progeny after KDs [*ND7* (negative control), *PGM* (positive control), *DevPF1* and *DevPF2*] during sexual development. For *DevPF1* and *DevPF2*, two silencing regions were targeted (a and b, see Fig. 4A). The numbers of experiments (*n*) and cells counted (cells) are indicated at the top. Survival: normal division. Sickness: reduced growth. Death: 3 or less cells after 3 days. (B) IES retention PCRs for two IESs on genomic DNA isolated from KD cells. Images are representative of five repeats. (C,D) IES retention score (IRS) histograms for *DevPF1* (C) and *DevPF2* (D) KD replicates, indicated in parentheses. The insets show the full scale. (E) IRS correlation between KDs. Diagonal, IRS distributions of individual KDs. Below diagonal, correlation graphs of pairwise comparisons. Above diagonal, corresponding Spearman correlation coefficients. Red lines, ordinary least-squares (OLS) regression; orange lines: locally weighted scatterplot smoothing (LOWESS); gray lines, orthogonal distance regression (ODR).

The influence of the DevPFs on genome reorganization was initially investigated with survival tests and IES retention PCRs upon KDs. Reduced protein levels during sexual development can induce errors including IES retention, impacting the survival of the subsequent generation. For survival tests, the growth of the cells that completed their sexual development was followed for several divisions. IES retention PCRs test for the presence (failed excision) of specific IESs in the new MAC genome. *ND7*-KD and *PGM*-KD were used as negative and positive control, respectively. To investigate the possibility that the observed effects result from off-target silencing of an unrelated gene, two silencing probes (a and b) were tested for each paralog (Fig. 4A). *DevPF1* and *DevPF2* KDs with either of the silencing probes resembled *PGM*-KD, with high lethality in the new progeny (Fig. 5A) and retention of selected IESs (Fig. 5B). This indicates that both *DevPF1* and *DevPF2* contribute to IES excision.

Next, we tested genome-wide IES retention in enriched new MAC DNA samples. We observed considerably elevated levels of retained IESs in both *DevPF1*- and *DevPF2*-KD (Fig. 5C,D). Notably, differences between replicates of the same KDs were observed, whereas replicate pairs processed in parallel (see Materials and Methods) exhibited similar profiles. Correlations among the paralog replicates indicated that despite varying IES retention distributions, *DevPF2*-KD replicates demonstrated high correlations among themselves (Fig. 4D). *DevPF1*-KD replicates correlated less well with each other, and *DevPF1*-KD replicate 3 (3) showed a high correlation with the *DevPF2*-KDs. This indicates that *DevPF2*-KD replicates were more consistent than the *DevPF1*-KD replicates.

Genes that work closely together are expected to show similar KD effects on IES retention. To identify functionally related genes, we examined correlations between the *DevPF1*-KD and *DevPF2*-KD IRS data and published data from other gene KDs (Fig. 5E). *DevPF2*-KD (4) was selected from the *DevPF2* replicates. *DevPF1*-KD (2) and *DevPF1*-KD (4) were selected as representative of the variability observed in the *DevPF1*-KDs. *DevPF2*-KD (4) displayed high correlations with other KDs, such as *TFIIS4* and the triple combination of *DCL2*, *DCL3* and *DCL5* (collectively denoted *DCL2/3/5*) (Fig. 5E). A moderate correlation was observed for *DevPF1*-KD (4) with *SPT5m*, whereas *DevPF1*-KD (2) did not correlate well with any of the tested KDs.

Short IESs are proposed to predominantly rely on the excision complex [specifically Pgm (Baudry et al., 2009) and Ku80c (Marmignon et al., 2014)] for removal, whereas long IESs tend to require additional molecules for excision (Sellis et al., 2021). To determine whether *DevPF1*- and *DevPF2*-KD preferentially affect long IESs, the length distribution of the top 10% of highly retained IESs in each KD was plotted (Fig. S4A,B; Table S1). In comparison to the length distribution of all IESs, *DevPF2*-KD (4) showed an overrepresentation of long IESs, similar to observations in *EZL1*-KD, silencing of the catalytic subunit of the PCR2 complex (Frapporti et al., 2019; Lhuillier-Akakpo et al., 2014) or *DCL2/3/5*-KD, and upon silencing of the scnRNA and iesRNA biogenesis proteins (Lepère et al., 2009; Sandoval et al., 2014) (Fig. S4A). Conversely, the highly retained IESs in *DevPF1*-KD (2) did not show the same overrepresentation and resembled the profile of *PGM*- and *KU80c*-KD, silencing of two members of the excision complex. Again, the replicates of the *DevPF* KDs exhibited variation in the extent of the observed effect (Fig. S4B).

Defects in IES excision not only result in the retention of IESs but can also lead to excision at alternative TA boundaries. So far, alternative excision above background levels has only been reported for silencing of ISWI1 and its complex partners (Singh et al., 2022, 2023 preprint). Neither *DevPF1*-KD nor *DevPF2*-KD resulted in elevated levels of alternative excision (Fig. S4, Table S2).

### *DevPF1*- and *DevPF2*-KD alter the small RNA population

**Fig. 6.**
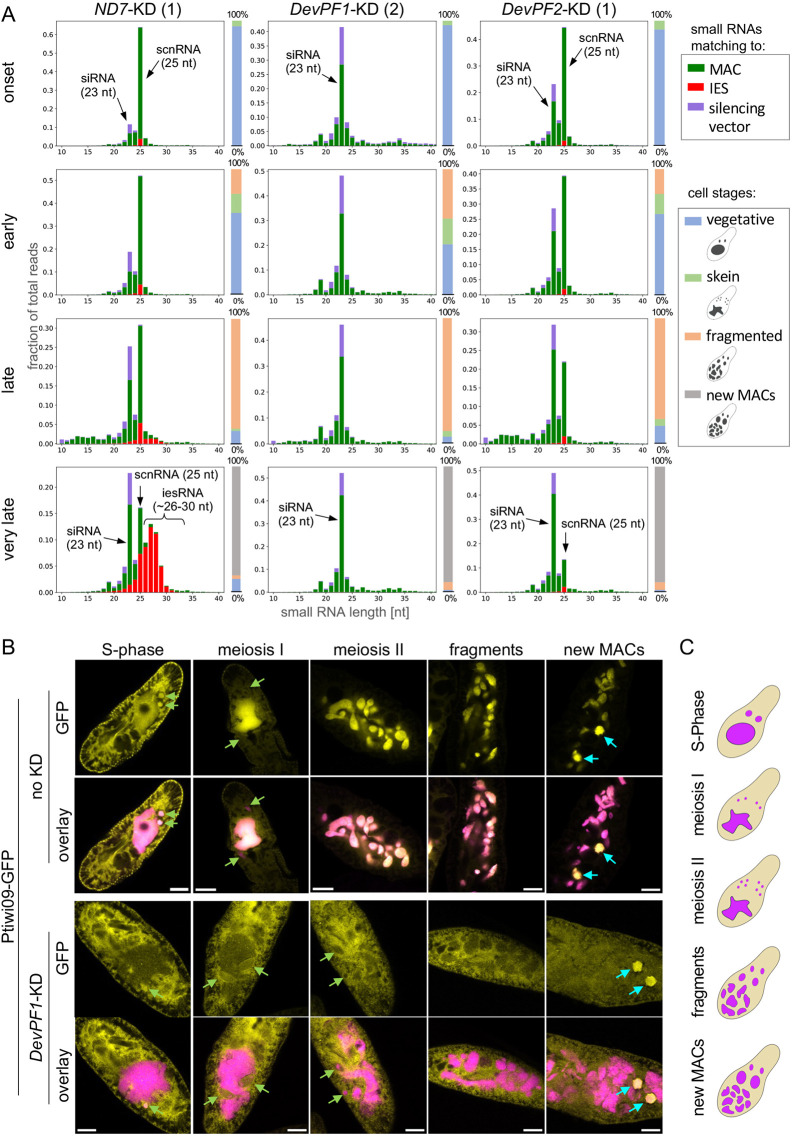
**Changes of small RNA populations upon *DevPF* knockdowns.** (A) Small RNA populations (10–40 nt) at developmental time points (onset, early, late and very late) in different KDs [*ND7* (control), *DevPF1* and *DevPF2*], mapping to silencing plasmid backbone (vector), MAC or IES sequences (*n*=1; graphs for replicates are shown in Fig. S6A). Individual cell stage compositions are indicated by the bar to the right of each diagram, along with schematic representations of the cell stages considered. (B) Ptiwi09–GFP localization at different developmental stages in the context of no (top) and *DevPF1* KD (bottom). DAPI (pink) and GFP (yellow). Individual *z*-planes for GFP and overlay (DAPI and GFP). Green arrows, MICs; cyan arrows, new MAC. Scale bars: 10 µm. Images are representative of ten repeats. (C) Schematic representation of cell stages in B.

The early-produced 25 nt scnRNAs and the late-produced 26–30 nt iesRNAs have been proposed to assist MIC-specific sequence targeting in the new MACs (Sandoval et al., 2014). Therefore, the small RNA populations across developmental time points upon DevPF KDs were analyzed (Fig. 6A; Fig. S6A). In *DevPF1*-KD (2), scnRNA production was completely abolished, an effect also observed upon the KD of genes proposed to be involved in scnRNA production, particularly, the two scnRNA-processing genes *DCL2* and *DCL3* (Sandoval et al., 2014), and *STP5m*, which is involved in the generation of the transcripts serving as substrates for Dcl2 and Dcl3 cleavage (Gruchota et al., 2017). The KD of the late-expressed *DevPF2* showed a much weaker reduction of scnRNA production, which might be caused by co-silencing of *DevPF1*.

To further investigate the effect of DevPF1 on the scnRNA pathway, we observed Ptiwi09–GFP localization upon *DevPF1*-KD. Ptiwi09, together with Ptiwi01, stabilizes the scnRNAs throughout scnRNA selection in the parental MAC and targeting of MIC-specific sequences in the new MACs (Bouhouche et al., 2011; Furrer et al., 2017). As previously described (Bouhouche et al., 2011; Singh et al., 2023 preprint), Ptiwi09–GFP localizes first to the cytoplasm and parental MAC with a transient localization in the swelling MICs before shifting to the new MAC (Fig. 6B). Upon *DevPF1*-KD (Fig. 6B), the localization to the MICs before meiosis I is not impaired; however, the translocation into the parental MAC is strongly reduced, and Ptiwi09–GFP predominantly remains in the cytoplasm throughout meiosis II and MAC fragmentation. We have reported a similar change in Ptiwi09–GFP localization upon *DCL2/3*-KD (Singh et al., 2023 preprint), suggesting that the loss of scnRNAs is responsible for the failed protein transfer into the parental MAC. Similar to what is seen in *DCL2/3*-KD, *DevPF1* depletion does not affect the localization of Ptiwi09–GFP to the new MACs (Fig. 6B).

Interestingly, DevPF1-HA immunoprecipitation (IP) at two developmental time points (early, ∼30% fragmentation; late, visible new MACs in fragmented cells) identified Ptiwi01 and Ptiwi09 as potential interaction partners of DevPF1 with a higher enrichment in the early than the late time point (Fig. S6B, Table S3). None of the other small RNA-related proteins were detected (Dcls, Spt5m, TFIIS4, Ptiwi10 or Ptiwi11).

For both *DevPF1-* and *DevPF2*-KD, iesRNA production was impaired. iesRNAs are proposed to derive from dsRNAs transcribed from excised IESs (Allen et al., 2017; Sandoval et al., 2014). Hence, failed excision of IESs in *DevPF1*- or *DevPF2*-KD contributes to reduced iesRNA levels, as has consistently been observed for many other KDs of genes involved in *Paramecium* genome editing (Charmant et al., 2023 preprint; de Vanssay et al., 2020; Ignarski et al., 2014; Maliszewska-Olejniczak et al., 2015; Singh et al., 2022; Wang et al., 2023 preprint). The lack of scnRNAs in the *DevPF1*-KD cannot explain the absence of iesRNAs, as these accumulate even if the preceding scnRNA production is blocked (Sandoval et al., 2014). In the late time point analyzed for DevPF IPs, peptides mapping to Ptiwi10, Ptiwi11 and Ptiwi06 were detected in the DevPF2 IP (Fig. S6C, Table S3), but not the DevPF1 IP (Fig. S6B). Therefore, DevPF2 might contribute to iesRNA biogenesis through an interaction with Ptiwi proteins.

### *DevPF1*- and *DevPF2*-KD affect mRNA expression

Given that PHD finger proteins have often been reported to be involved in gene expression regulation (Aasland et al., 1995; Sanchez and Zhou, 2011) we sought to investigate whether the *DevPF* KDs alter mRNA expression levels during development. Batch effects had a major influence on the variance within the replicates (Fig. S7A), as observed for IES retention (Fig. S5C,D).

**Fig. 7.**
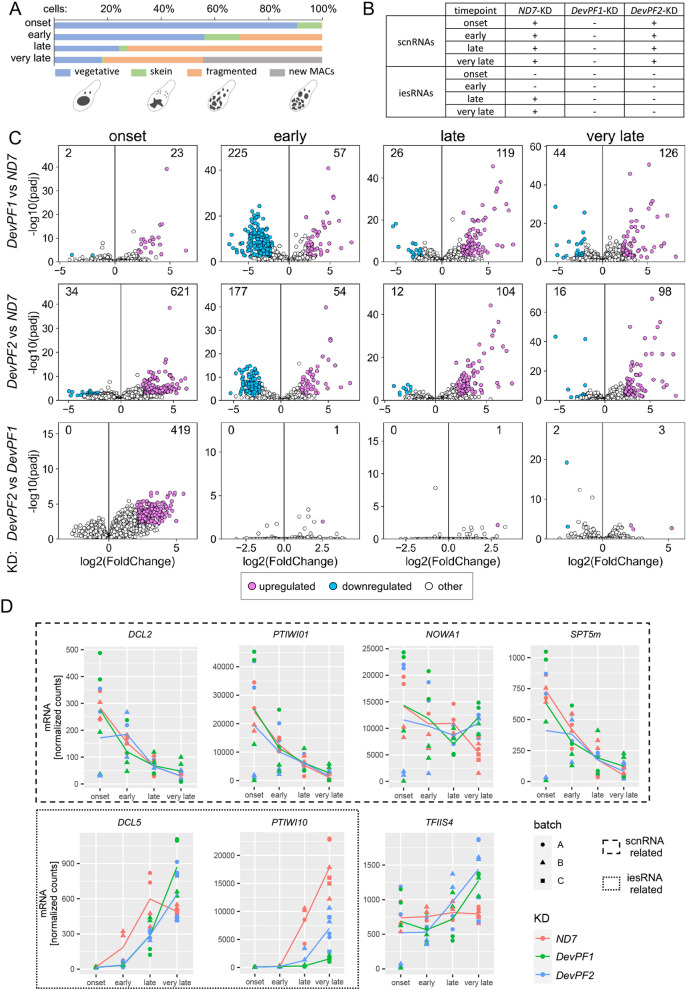
**Differential gene expression in *DevPF* knockdowns.** (A) Cell stage composition of each time point averaged over all KDs (individual compositions in Fig. S5), along with schematic representations of the considered cell stages. (B) Presence or absence of scnRNAs and iesRNAs in different KDs (*ND7*, *DevPF1* and *DevPF2*) and time points (onset, early, late and very late). (C) Differentially expressed genes in *DevPF1*- (top) or *DevPF2*- (middle) compared to *ND7*-KD or *DevPF1*-KD compared to *DevPF2*-KD (bottom) at different developmental time points (onset, early, late and very late). Thresholds for up- and down-regulation: adjusted *P*-value<0.01; |log2(fold change)|>2. The number of up- and down-regulated genes is indicated in each diagram. For all comparisons, 35,777 transcripts were analyzed, except for: DevPF1-ND7 onset (33,696), DevPF2-ND7 early (35,083), and DevPF2-DevPF1 onset (34,389). (D) Gene expression levels of selected genes upon KDs [*ND7* (control), *DevPF1* and *DevPF2*] at different developmental time points (onset, early, late and very late). The lines represent the mean of all replicates (*n*=4 for all stages except for ‘very late’, where it is 6) in a given KD and time point.

*DevPF1*-KD showed almost no differentially expressed genes compared to *ND7*-KD during the onset of development (Fig. 7A–C). During this early stage, genes are transcribed solely from the parental MAC, where DevPF1–GFP does not localize (Figs 2A, 3). Surprisingly, in *DevPF2*-KD, a high number of genes were differentially expressed during the onset of development (Fig. 7A,C). Given that *DevPF2* is late-expressed and *DevPF1*-KD showed no effect, the observed difference might be caused by differing cell stages within the collected populations of *DevPF2*-KD and *ND7*-KD. During the ‘early’, ‘late’ and ‘very late’ time points, *DevPF1*- and *DevPF2*-KD showed similar changes in mRNA expression.

The abolishment of development-specific small RNAs in the *DevPF*-KDs might result from downregulation of genes involved in scnRNA or iesRNA production. We observed no general trend indicating a drastic reduction of expression of scnRNA-related genes, like *DCL2*, *PTIWI01* or *SPT5m* (Fig. 7B,D; Fig. S7B, Tables S4, S5). However, these trends in expression should be considered with the caveat of considerable expression variability and limitation of the number of replicates that could practically be obtained. At least for Ptiwi09, the localization experiments upon *DevPF1*-KD confirmed no loss in protein levels (Fig. 6B).

The expression of iesRNA-related genes was altered in both *DevPF1*- and *DevPF2*-KD compared to *ND7*-KD (Fig. 7D; Fig. S7B, Tables S4, S5). *DCL5*, the Dicer-like protein responsible for the initial cleavage of IES derived dsRNAs into small iesRNAs (Sandoval et al., 2014), was downregulated (Tables S4, S5) in early stages, but tended to be upregulated in the very late stage (Tables S4, S5). *PTIWI10/11*, which encode the Piwi proteins responsible for further processing and stabilization of iesRNAs during the positive feedback loop (Furrer et al., 2017), were downregulated in both *DevPF1*- and *DevPF2*-KD (Tables S4, S5). Successful expression of *PTIWI10*/*11* has been proposed to depend on IES excision since both genes are expressed from the new MAC and harbor IESs in their flanking or coding regions (Furrer et al., 2017) (Fig. S7C). If IES retention were the only cause for downregulation, one would expect higher IRSs for these IESs in KDs with lower mRNA levels. Although the mRNA reduction is stronger in *DevPF1*-KD than in *DevPF2*-KD (Fig. 7D; Table S4, S5), this trend is not reflected in the IRSs of the IESs whose retention is proposed to interfere with *PTIWI10*/*11* expression (Table 1). In most of the KD replicates, there is no or low retention (IRS<0.1) and the replicates showing moderate to high retention (0.1<IRS<0.3) belong to both *DevPF1*- and *DevPF2*-KD. Hence, the reduced mRNA levels of *PTIWI10*/*11* cannot only be explained by IES retention.

**Table 1.**
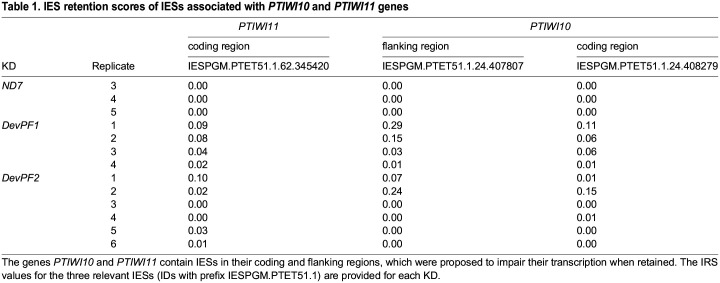
IES retention scores of IESs associated with *PTIWI10* and *PTIWI11* genes

## DISCUSSION

### Implications of the PHD domain for DevPF1 and DevPF2 functions

Genome reorganization is a fundamental process underlying cell and immune system development and some diseases (Bassing et al., 2002; Forment et al., 2012; Mani and Chinnaiyan, 2010; Rooney et al., 2004). Ciliates undergo massive genome reorganization during the maturation of their somatic genome. This makes them excellent models for studying the complex mechanisms involved in the targeted elimination of genomic sequences (Beisson et al., 2010a).

In the present study, we described two paralogous PHD finger proteins, DevPF1 and DevPF2, involved in IES excision in *Paramecium*. Both paralogs harbor a PHD and a PHD zinc finger-like domain (Fig. 1). These domains belong to the zinc-finger family, and the PHD domain is characterized by a well-conserved C4HC3 motif (Aasland et al., 1995; Schindler et al., 1993). The eight core amino acids of this motif coordinate two zinc ions and thereby provide structural stability to the domain (Pascual et al., 2000). Among other histone-binding domains, such as bromodomains and PWWP domains, PHD fingers are the smallest (Fleck et al., 2021; Miller et al., 1985). Multiple sequence alignment and structure predictions confirmed the presence of the characteristic C4HC3 motif in both DevPF1 and DevPF2 (Fig. 1), suggesting that both PHDs might be functional.

Epigenetic marks, such as DNA methylation and histone modifications, are frequently associated with the regulation of programmed DNA elimination (Wang and Davis, 2014; Dedukh and Krasikova, 2022; Goday and Pigozzi, 2010). PHD fingers are often epigenetic readers, recognizing histone modifications, primarily on the histone 3 (H3) N-terminal tail (Sanchez and Zhou, 2011). Peptides matching to histones were enriched in the DevPF-IPs of late developmental time points (Fig. S6B,D, Table S3); however, none of them was specific to H3. PHD fingers have been reported to bind non-H3 partners, like DNA, histone 4, or other proteins (Bienz, 2006; Black and Kutateladze, 2023; Gaurav and Kutateladze, 2023; Liu et al., 2012; Oppikofer et al., 2017). The combination of the PHD and PHD-zinc-finger-like domain in the DevPFs might enable the paralogs to simultaneously recognize two adjacent histone modifications, as demonstrated for tandem PHD domains (Zeng et al., 2010).

An ortholog of the DevPFs, Nmp1, associates with a condensin complex protein in *Tetrahymena* and is present in its new MACs (Howard-Till et al., 2019). However, the localization of Nmp1 in vegetative MACs means it is not restricted to development. This differs from what is seen for the development-specific DevPFs. Given the duplication that created DevPF1 and DevPF2, and differences in localization between them, important differences have clearly evolved between these proteins and Nmp1. Recently one of two paralogs of a condensin complex subunit SMC4, has been reported to be required for *Paramecium* DNA elimination (Zhang et al., 2024). However, that study does not report on associated PHD finger proteins. We observed no enrichment of *Paramecium* SMC4 homologs or *Tetrahymena* condensin protein (Cpd2) homologs in the DevPF IP mass spectrometry data. Nevertheless, in future, it would be worthwhile using other experimental techniques to investigate whether either of the DevPFs associate with a condensin complex involved in DNA elimination.

PHD domains are also found in various chromatin-associated proteins involved in gene regulation. Notably, ISWI-containing chromatin remodeling complexes often include a subunit with a PHD domain, such as the ACF (Eberharter et al., 2004), NURF (Li et al., 2006; Wysocka et al., 2006) or WICH (Bozhenok et al., 2002) complexes. DevPF2 was initially identified in ISWI1 protein pulldowns, and, thus far, no PHD-containing protein has been shown to be a part of this remodeling complex (Singh et al., 2022, 2023 preprint). It is intriguing to consider that DevPF2 might contribute PHD functionality to the ISWI1 chromatin remodeling complex. However, *DevPF2*-KD does not show elevated levels of alternative excision (Fig. S4C–E), which is a characteristic of other members of the complex identified so far (Singh et al., 2022, 2023 preprint) and ISWI1 was not identified as a potential interaction partner in the DevPF2-IP (Fig. S6C). If DevPF2 interacts with the ISWI1 complex, we infer that it might not be a core complex component, particularly as it does not contribute to excision precision.

### A potential role for DevPF1 and DevPF2 as transcription factors?

#### A potential role in non-coding transcription in the MICs for scnRNA production

The localization of DevPF1 in the MICs (Figs 2A, 3) and its importance for scnRNA production (Fig. 6A) could point towards its involvement in the bidirectional transcription of the MIC genome for scnRNA production. Spt5m (Gruchota et al., 2017) and TFIIS2 and TFIIS3 (Maliszewska-Olejniczak et al., 2015) are proposed to be involved in this transcription. One of the *DevPF1*-KD replicates showed moderate IRS correlation with *SPT5m* (Fig. 5E) (to our knowledge, no IRS data exists for TFIIS2 or TFIIS3) and *SPT5m*-KD also reduces scnRNA production. The localization of Dcl2–GFP (Lepère et al., 2009), Ptiwi09–GFP (Fig. 6B) and DevPF1–GFP (Fig. 2A) in the swelling MICs suggests that scnRNA biogenesis occurs during the meiosis S-phase. Ptiwi09 and DevPF1 might interact in the MICs or the cytoplasm. Non-crosslinked IPs would be needed to further verify this interaction. However, *PTIWI01*/*09*-KD does not completely abolish scnRNAs (Furrer et al., 2017), indicating that DevPF1 acts upstream of scnRNA loading and guide strand removal. Future investigations of bi-directional transcription and scnRNA biogenesis will allow identification of how these molecules cooperate.

Spt5m–GFP, TFIIS2– and TFIIS3–GFP and DevPF1–GFP are present in the MICs beyond S-phase and localize to the new MACs at later stages (Gruchota et al., 2017; Maliszewska-Olejniczak et al., 2015). Their role in the MIC during meiotic divisions remains unknown. It has been speculated that Spt5m might be involved in co-transcriptional deposition of epigenetic marks that sustain meiotic processes, ultimately aiding in IES targeting (Gruchota et al., 2017). The potential of PHD domains to bind histone modifications raises a similar possibility for DevPF1. However, its role appears to be more specific, as DevPF1 is not present in all gametic and zygotic nuclei simultaneously (Figs 2, 3).

Msh4 and Msh5, homologs of proteins essential for crossover, are also present in all gametic nuclei during the first and second meiotic division, and their silencing leads to substantial IES retention (Rzeszutek et al., 2022). However, their non-canonical functions that lead to IES retention are not yet fully understood (Rzeszutek et al., 2022). Given that new MACs develop in *DevPF1*-KD (Fig. 6B, Fig. S3) and *MSH5*-KD cells, neither of the genes are essential for crossover or karyogamy. More research will be needed in future to decipher the functions of the DevPF proteins in the gametic nuclei.

#### A potential role in non-coding transcription in the new MAC for scnRNA-based targeting and iesRNA production

Non-coding transcription in the new MAC, which is hypothesized to generate substrates for scnRNA pairing, has been proposed to be regulated by the putative transcription elongation factor TFIIS4 that specifically localizes to the early new MACs (Maliszewska-Olejniczak et al., 2015). *DevPF2*-KD IRSs of some replicates correlated most strongly with *TFIIS4*-KD (Fig. 5E), pointing towards a shared functionality. Both DevPF1 and DevPF2 have the potential to act in the same regulatory process as TFIIS4 because both their GFP fusions localize to the new MACs. In fact, there are reports of transcription factors that combine the TFIIS and PHD domains – Bypass of Ess1 (Bye1) protein in S*accharomyces cerevisiae* harbors a PHD and a TFIIS-like domain, with the former recognizing histone 3 lysine 4 trimethylation and the latter establishing contact with polymerase II for transcriptional regulation (Kinkelin et al., 2013; Pinskaya et al., 2014). It is possible that similar functionality is separated on two individual proteins in *Paramecium*. However, TFIIS4 was not detected in either of the DevPF IPs in the late developmental stage.

The production of iesRNAs has also been proposed to depend on the non-coding transcription of excised IESs (Allen et al., 2017; Sandoval et al., 2014). Little is known about the proposed bidirectional transcription to produce substrates for Dcl5 cleavage, though Allen et al. speculated on the involvement of TFIIS4. Given that iesRNA production is almost completely abolished in *DevPF1*- and *DevPF2*-KD, a contribution to this transcription is plausible. The potential function of the DevPFs might extend far beyond TFIIS4-dependent transcription; whereas TFIIS4–GFP localizes transiently to early new MACs (Maliszewska-Olejniczak et al., 2015), DevPF2–GFP and DevPF1–GFP remain in the new MACs far longer (Fig. 2).

#### A potential role in gene transcription in the parental and the new MAC

Early in development, the parental MAC is solely responsible for gene expression and, after genome reorganization progresses, the new MAC contributes at later stages (Berger, 1973). In *Tetrahymena*, E2F family transcription factors have been shown to control the cell cycle through gene expression during meiosis (Zhang et al., 2018). DevPF1 and DevPF2 are unlikely to be active in the parental MAC given that none of the GFP fusion proteins localized there (Fig. 2). Consistent with this, *DevPF1*-KD showed no differential gene expression compared to *ND7*-KD during the onset of development (Fig. 7C) and Ptiwi09–GFP expression was not impaired upon *DevPF1*-KD (Fig. 6B). However, it is difficult to reach a definite conclusion for other genes due to the high variability in expression between the replicates (Fig. 7D; Fig. S7B) and the high number of differentially expressed genes in *DevPF2*-KD (Fig. 7C) observed during the onset of development. Cells in the ‘onset’ time point are challenging to collect because cell staging relies on MAC morphology changes visualized by DAPI staining. Truly vegetative cells cannot be distinguished from cells initiating meiosis given that their MACs look the same; however, the gene expression profiles are expected to differ substantially (Fig. 2A; Fig. S2B). The collection of subsequent time points is more reliable because the alteration of old MAC shape as development progresses is pronounced.

At the subsequent stages, *DevPF1*- and *DevPF2*-KD affected similar genes. Either the changes are nonspecific to the *DevPF*-KDs and result from the proposed nuclear crosstalk to adjust transcription levels to accommodate for failed IES excision (Bazin-Gélis et al., 2023) or they are specific to the *DevPF*-KDs and both paralogs exhibit similar functions in the regulation of gene expression. Interestingly, differential expression was observed at the ‘early’ time point (Fig. 7C). *GTSF1*-KD, also causing substantial IES retention, hardly shows any differentially expressed genes at a comparable stage [*DevPF1*-KD and *DevPF2*-KD have 282 and 231 differentially expressed genes, respectively, at ∼30% fragmentation (Fig. 7C); *GTSF1*-KD has 10 differentially expressed genes at ∼30–50% fragmentation (Wang et al., 2023 preprint)]. This indicates that the early change in gene expression might be specific to *DevPF*-KDs, potentially mediated by other proteins shuttling into the parental MAC. Given that Ptiwi09–GFP translocates efficiently to the parental MAC upon *GTSF1*-KD (Wang et al., 2023 preprint) but not upon *DevPF1*-KD (Fig. 6B), it might be worth investigating differential expression upon *PTIWI01/09*-KD.

Late in development, gene expression starts from the new MACs (Berger, 1973), where both DevPF paralogs localized (Fig. 2). Some late-expressed genes, like *PTIWI10*, are expressed only from the new MAC after the initial onset of IES excision (Furrer et al., 2017). Indeed, *PTIWI10/11* mRNA levels are downregulated in *DevPF1*-KD or *DevPF2*-KD (Fig. 7D, Fig. S7B, Tables S4, S5). This trend cannot be explained solely by the strength of retention observed for the IESs interfering with *PTIWI10*/*11* expression (Table 1). It suggests that DevPF1 and DevPF2 might regulate gene expression in the new MAC, albeit specifically for some genes like *PTIWI10/11.* The extent of gene expression regulation by the DevPFs beyond these genes remains uncertain. To further investigate whether the DevPFs serve as transcription factors, and if so, which genes they regulate, genes associated with DevPF binding could be identified by techniques like ‘cut-and-run’ (Skene et al., 2018) and compared to mRNA expression changes upon *DevPF*-KDs.

### Potential cytoplasmic functions

In contrast to the other putative transcription factors discussed so far (Spt5m, TFIIS2, TFIIS3 and TFIIS4, and DevPF2), DevPF1–GFP exhibits a pronounced cytoplasmic distribution in the early stages of development (Fig. 2A). Although most described PHD fingers are nuclear proteins, some can be recruited to the cytoplasm or plasma membrane by binding partners (Betz et al., 2004; Gozani et al., 2003). DevPF1 might play a role in transmitting signals of sensed starvation to the MICs, initiating sexual development. As *DevPF1* is not constitutively expressed during vegetative growth (Fig. 1B; Fig. S2B), another factor is needed to first initiate gene expression of *DevPF1* in the parental MAC. However, DevPF1 might interact with specific markers of starvation in the cytosol, promoting early sexual processes. If that is the case, DevPF1 is not essential for general meiotic processes, as neither meiosis nor new MAC development show defects in *DevPF1*-depleted cells (Fig. 6B, Fig. S3). Given that peptides matching Ptiwi01/09 were identified in the DevPF -IP, the Ptiwi01/09 complex is a potential binding partner of DevPF1 in the cytoplasm. However, as Ptiwi01 and Ptiwi09 are highly expressed proteins (Bouhouche et al., 2011), further IP experiments would be needed to verify this interaction.

### The selective localization of DevPF1 to gametic and post-zygotic nuclei

The selective localization of DevPF1 to certain gametic and post-zygotic nuclei (Fig. 3) raises intriguing questions about its potential role in nuclear fate decisions. The survival and destruction of the gametic nuclei depends on their subcellular positioning (Grandchamp and Beisson, 1981). DevPF1 might play a role in either promoting their movement or preparing for their degradation. However, the observed number of nuclei simultaneously containing DevPF1–GFP (zero to four) neither fits the number of nuclei selected for survival (one) nor for degradation (seven). DevPF1 might either contribute to this process successively or its role might not be directly related to the nuclear fate itself. The fate of the post-zygotic nuclei is decided during the second mitotic division by the subcellular localization of the division products (Grandchamp and Beisson, 1981). This means, from each post-zygotic nucleus, one of the division products will remain as MIC and one develops into a new MAC. During the second mitotic division, DevPF1–GFP was observed in one of the two dividing nuclei. Its localization in the precursor of one MIC and one MAC without being present in the precursor of the other MIC and MAC, does not imply its involvement in the nuclear fate decision. Furthermore, *DevPF1*-KD neither impaired the selection of gametic nuclei nor the differentiation of the new MACs.

The specific localization of nuclear proteins to certain nuclei in multinuclear cells has been studied extensively in insect embryos. In *Drosophila*, the transcription factors Bicoid (Driever and Nüsslein-Volhard, 1988) and Dorsal (Roth et al., 1989) establish the anterior-posterior and dorsal-ventral axis, respectively, by initiating gene expression depending on the cytoplasmic localization of the nuclei. The activity of the transcription factors is restricted by gradients to a certain cytoplasmic region (Morisato and Anderson, 1995; Spirov et al., 2009). However, the nuclear localization of DevPF1–GFP does not appear to be associated with subcellular localization of the nuclei and it remains unclear how DevPF1–GFP is specifically recruited.

As only fixed cells were examined, the dynamics of DevPF1–GFP localization were not captured. The fact that DevPF1–GFP localization is independent of nuclear divisions (Fig. 3B), combined with observations of cells at the meiotic or mitotic division stage with an absence of DevPF1–GFP in all nuclei (Fig. S2C), suggests that DevPF1 localization might be asynchronous and transient. Possibly it is recruited to each of the gametic nuclei at some point before the completion of the second meiotic division and to each of the post-zygotic nuclei before completion of the second mitotic divisions. Live-cell imaging could illuminate the dynamics of DevPF1 localization and its correlation with nuclear fate. However, this approach presents technical challenges due to the requirement of confocal imaging to capture DevPF1–GFP MIC localization, and the observation time scale needing to span across hours of *Paramecium* development.

### DevPF1 – a general factor for IES excision

DevPF1 plays a role throughout sexual development, from the early stages before meiosis to the very late stages (Fig. 2A). It appears to influence various aspects of genome reorganization in the MICs and the new MACs, including scnRNA production and potentially expression of certain genes. Consequently, the depletion of *DevPF1* affects the excision of a wide range of IESs (Fig. 5C). However, it is important to reiterate that we observed high batch-to-batch variability in the *DevPF* replicates in both IES retention (Fig. 5C,D) and mRNA expression (Fig. 7D; Fig. S7B). The collection time points had a major influence on mRNA levels (Fig. S7A). Variable new MAC enrichment by a sucrose gradient might introduce variation into the IRS analysis, as fragments of the parental MAC add unexcised IES sequences, diluting the effect of IES retention (Charmant et al., 2023 preprint). Fluorescence-activated nuclear sorting (FANS) enables better nuclear separation in *Paramecium* (Charmant et al., 2023 preprint; Guérin et al., 2017) and should be able to eliminate most of such variation. Additionally, microinjection of DNA into macronuclei before RNAi experiments can be used to control for contaminating DNA from old MAC fragments.

Revisiting previous KD experiments with additional replicates would be worthwhile to explore the extent of batch-to-batch IRS and expression variance for other KDs. It is noteworthy that variability in IES retention across replicates has recently been shown for *GTSF1* (Charmant et al., 2023 preprint*;* Wang et al., 2023 preprint), suggesting this phenomenon is not restricted to *DevPF1* and *DevPF2*. In general, KD experiments are challenging to tightly control for reproducibility, and more effort should be invested in generating knockouts in *Paramecium*, as established in *Tetrahymena* (Chalker, 2012).

It has been shown that evolutionarily old IESs tend to be short and excised early in development, and that this occurs independently of additional factors apart from the excisase (Sellis et al., 2021). On the other hand, evolutionarily young IESs tend to be long, excised later and dependent on the scnRNA pathway and histone modification deposition in the new MAC for their excision (Sellis et al., 2021; Swart et al., 2014; Zangarelli et al., 2022). In line with this, most gene KDs tested in this study exhibited an overrepresentation of long IESs among their most highly retained IESs, including that for *DevPF2* (Fig. S4A,B). Only *PGM*-KD, *KU80c*-KD and two of the *DevPF1*-KD replicates showed no preference for long IESs. Pgm and Ku80c are components of the excision machinery and are therefore expected to affect all IESs. Although DevPF1 might not be a direct part of the excision machinery, it appears to have a general contribution to IES excision, regardless of the length of the IES. Consequently, we propose that DevPF2 contributes to the excision of long IESs, whereas DevPF1 might serve as a more general factor.

## MATERIALS AND METHODS

### *Paramecium tetraurelia* cultivation

Mating type 7 (MT7) cells from strain 51 of *Paramecium tetraurelia* were grown in wheat grass powder (WGP, Pines International) medium supplemented with 10 mM sodium phosphate buffer (pH 7.3). WGP medium was bacterized with *E. coli* strain HT115 (DE3) (a gift from Mariusz Nowacki, University of Bern, Switzerland) to feed paramecia, and the cultures were maintained either at 27°C or at 18°C according to the standard protocol (Beisson et al., 2010b,c).

### Protein localization imaging by fluorescence microscopy

Plasmids for microinjection were generated by amplifying the coding and flanking sequences from MT7 genomic DNA and introducing them with the PCR-based method CPEC (Quan and Tian, 2011) into the L4440 plasmid (Addgene #1654). DevPF1 was expressed with its endogenous flanking regions (304 bp upstream of the DevPF1 start codon and 272 bp downstream of the DevPF1 stop codon). DevPF2 endogenous flanking regions (455 bp upstream the DevPF2 start codon and 273 bp downstream of the DevPF2 stop codon) yielded no expression. Therefore, as *PGM* exhibits a similar expression profile to *DevPF2* (Fig. 1B), DevPF2 genomic coding sequence was inserted between the *PGM* flanking regions (96 bp upstream of the *PGM* start codon and 54 bp downstream of the *PGM* stop codon). Before the stop codon, the *GFP* coding sequence was connected to the protein coding sequences via a glycine-serine-linker (SSGGGSGGSGGGS). 60 μg of plasmid DNA was linearized with AhdI (New England Biolabs, UK) and extracted with phenol-chloroform for injection.

*Paramecia* were microinjected with either C-terminally GFP-tagged DevPF1 (endogenous regulatory regions) or C-terminally GFP-tagged DevPF2 (*PGM* regulatory regions) following the standard protocol (Beisson et al., 2010d). Sexual development was induced by starvation and cells of different developmental stages were collected and stored in 70% ethanol at −20°C. To stain cells with DAPI, cells were dried on a microscopy slide, washed twice with phosphate-buffered saline (PBS) and permeabilized for 10 min at room temperature (RT) with 1% Triton X-100 in PHEM buffer (5× PHEM: 50 mM EGTA, 125 mM HEPES, 10 mM MgCl_2_, 300 mM PIPES, pH 6.9), fixed with 2% paraformaldehyde (PFA) in PHEM and washed once for 5 min at RT with 3% bovine serum albumin (BSA, Merck-Sigma, Germany) in Tris-buffered saline (TBS) with 10 mM EGTA and 2 mM MgCl_2_ (TBSTEM). After DAPI (2 μg/ml in 3% BSA) incubation for 7–10 min at RT. The cells were mounted 40 µl of ProLong Gold Antifade mounting medium (Invitrogen, USA) or ProLong Glass Antifade mounting medium (Invitrogen, USA). For α-tubulin staining, after permeabilization and fixation, cells were blocked for 1 h at RT with 3% BSA and 0.1% Triton X-100. Primary rat anti-α-tubulin antibody (YOLI/34, ab6161, Abcam, UK) was diluted 1:200 in 3% BSA and 0.1% Triton X-100 in TBSTEM and incubated overnight at 4°C. After three washes with 3% BSA in TBSTEM, goat anti-rat-IgG secondary antibody conjugated to Alexa Fluor 568 (ab175476, Abcam, UK) was diluted 1:500 in 3% BSA and 0.1% Triton X-100 in TBSTEM and incubated for 1 h at RT. After two washes, cells were stained with DAPI and mounted with Prolong Glass Antifade mounting medium.

Images were acquired on a confocal SP8 Leica fluorescence microscope (60×/1.4 oil objective) with constant laser settings. The detector (photon multiplier) gain for the DAPI signal (430–470 nm) varied to accommodate differences in signal strength (500–550 V). Postprocessing was done in Fiji software (version 2.14.0/1.54f) (Schindelin et al., 2012). The laser settings for the GFP signal were set the same in the all the images to be compared between wild type and knockdown samples (Fig. 6B). Brightness and contrast in the GFP channel were set the same in all the images to be compared (Fig. 2, Fig. S2B: DevPF1–GFP: Min 0, Max 681 and DevPF2-GFP: Min 0, Max 170; Fig. 3, Fig. S2C: DevPF1–GFP: Min 0, Max 703; Fig. S3: constant settings for each cell stage).

### Knockdown efficiency validation using fluorescence intensity

Cells injected with either DevPF2–GFP or DevPF1–GFP were subjected to KDs of *ND7*, *PGM*, *DevPF2* and *DevPF1* genes. Cells during new MAC development were collected (for details, see the section on silencing experiments), then stained with DAPI and mounted on ProLong Glass Antifade as described above. Images of a single *z*-plane through the new MAC were acquired on a SP8 Leica Confocal microscope with 60×/1.4 oil objective using the same laser settings for all images. For each KD, 10 cells were imaged. In Fiji software (version 2.14.0/1.54f), the brightness and contrast in the GFP channel was set the same values for all images compared in the same analysis (DevPF1–GFP-injected cells: Min 0, Max 1078; DevPF2–GFP-injected cells: Min 0, Max 298). Fluorescence signal was measured in a constant area in 1 MAC of each cell and the area mean was used as intensity for this nucleus. The area was set in the DAPI channel, and the fluorescence was measured in the GFP channel. Given that the same area was measured for each nucleus, no normalization was used to account for nuclear size variation. To account for background fluorescence, GFP fluorescence in non-transformed wild type cells was measured and the mean of all wild type cells was subtracted from all measured intensities. All intensities were normalized to the mean of all *ND7*-KD cells in the corresponding injection. In house scripts were used for quantification of fluorescent signals.

### Co-immunoprecipitation

Paramecia were injected with either Human influenza hemagglutinin (HA)-tagged DevPF1 (same cloning strategy as described for protein localization) or GFP-tagged DevPF2. For DevPF1–HA, an early time point (∼30% fragmentation) and a late time point (new MACs clearly visible in fragmented cells) was collected, whereas for DevPF2–GFP, only the late time point was collected. Non-transformed wild-type cells were collected as controls. Cells were washed twice with 10 mM Tris and as much liquid was removed as possible. For 300 ml initial culture volume, cells were fixed with 1 ml 1% PFA for 10 min at RT and quenched with 100 µl of 1.25 M glycine for 5 min at RT. After one wash with PBS (centrifugation for 1 min at 4°C and 1000 ***g***), 2 ml lysis buffer [50 mM Tris-HCl pH 8.0, 150 mM NaCl, 5 mM MgCl_2_, 1% Triton X-100, 10% Glycerol and cOmplete protease inhibitor EDTA-free (Roche, Germany)] were added and cells were sonicated using an MS72 tip on a Bandelin Sonopulse device with 52% amplitude for 15 s on ice. The pellet and input fraction were separated by centrifugation (13,000 ***g***, 4°C, 30 min).

To enrich HA-tagged proteins, 50 µl beads (anti-HA-affinity matrix, Merck-Sigma, Germany) were washed three times (500 g, 4°C, 2 min) in ice-cold IP buffer [10 mM Tris-HCl pH 8, 150 mM NaCl, 1 mM MgCl_2_, 0.01% NP-40, 5% glycerol and cOmplete protease inhibitor EDTA-free (Roche, Germany)] and incubated with 1 ml of cleared input lysate overnight at 4°C. After four washes with ice-cold IP buffer, the bound proteins were eluted from the beads in 50 µl 2× PLB (10% SDS, 0.25M Tris-HCl pH 6.8, 50% glycerol, 0.2M DTT and 0.25% Bromophenol Blue) at 98°C for 20 min (IP fraction).

To enrich GFP-tagged proteins, 25 µl beads (GFP-Trap Agarose beads, Chromotek, Germany) were washed once with ice-cold 20 mM Tris pH 7.5 with 100 mM |NaCl (2500 g, 4°C, 5 min) and three times in ice-cold IP buffer. Beads were incubated with 1 ml cleared input lysate for 1 to 2 h at 4°C and washed four times with ice-cold IP buffer. Bound proteins were eluted in 30 µl 2× PLB at 98°C for 20 min (IP fraction).

For western blots, 0.5% of total input and 15% of total IP fraction were resolved on 10% SDS-PAGE gels and wet transferred onto a 0.45 µm nitrocellulose membrane for 2 h at 80 V and 4°C (Bio-Rad, Germany). The membrane was blocked for 1 h in 5% BSA in PBS plus 0.2% Tween 20 (PBST). HA-tagged proteins were detected with an HRP-conjugated anti-HA antibody (sc-7392 HRP, Santa Cruz Biotechnology, USA) diluted 1:500 in PBST and incubated overnight at 4°C. GFP-tagged proteins were detected with an primary anti-GFP antibody (ab290, Abcam, UK) diluted 1:2000 and incubated overnight at 4°C followed by an secondary anti-rabbit-IgG HRP-conjugated antibody (12-348, Merck Millipore, Germany) diluted 1:5000 in PBST and incubated for 1 h at RT. Membranes (Figs S10, S11) were screened using an AI600 imager (GE Healthcare, Germany).

Samples were sent to EMBL's Proteomics Core Facility in Germany for mass spectrometry experiments and analysis. Using R software, contaminants were removed from the FragPipe output files (protein.tsv; Kong et al., 2017), and only proteins quantified with a minimum of two razor peptides were included for subsequent analysis. After log2 transformation of raw TMT reporter ion intensities, batch effect correction (‘removeBatchEffects’ function from the limma R package; Ritchie et al., 2015), and variance stabilization normalization (vsn) with the vsn package (Huber et al., 2002), the abundance difference in WT and DevPF samples was maintained by determining different normalization coefficients. To investigate differential protein expression (limma package), replicate information was incorporated in the design matrix with the ‘lmFit’ limma function. ‘hit’ annotation: false discovery rate (FDR) smaller 5% and a fold change of at least 100%. ‘candidate’ annotation: FDR smaller 20% and a fold change of at least 50%. In house scripts were used for generation of volcano plots.

### Silencing experiments, survival test and IES retention PCR

Silencing constructs for *DevPF2* and *DevPF1* were generated by cloning genomic gene fragments into a T444T plasmid (Sturm et al., 2018) (Addgene #113081) using CPEC (Quan and Tian, 2011). For both *DevPF1* and *DevPF2*, two silencing regions were selected: DevPF1 silencing region a (525 bp fragment from 3–527; position 1 is the first nucleotide of the start codon); DevPF1 silencing region b (733 bp fragment from 532–1264); DevPF2 silencing region a (525 bp fragment from 3–527); DevPF2 silencing region b (731 bp fragment from 532–1262). Co-silencing was predicted with the RNAi off-target tool from ParameciumDB (Li and Durbin, 2009) for both silencing regions (*DevPF1* silencing region a and b: 19 and 30 hits, respectively, in *DevPF2* gene; *DevPF2* silencing region a and b: 19 and 30 hits, respectively, in *DevPF1* gene). The plasmids were transformed into HT115 (DE3) *E. coli* strain and expression was induced overnight in 1× WGP medium at 30°C with Isopropyl β-D-1-thiogalactopyranoside (IPTG; Carl Roth, Germany). Paramecia were seeded into the resulting silencing medium at a density of 100 cells/ml to induce sexual development by starvation after 4 to 6 divisions. KD experiments were performed as previously described (Beisson et al., 2010e).

After the paramecia finished sexual development, 15 cells were transferred into a regular, non-induced, feeding medium for the survival test. Paramecia were monitored for 3 days to observe growth effects. For IES retention PCRs, genomic DNA was extracted from cultures that finished sexual development using GeneElute – Mammalian Genomic DNA Miniprep Kit (Merck-Sigma, Germany). PCRs were done on specific genomic regions flanking an IES (Table S6) to check for the retention of IESs. 1–12.5 ng DNA was used as input and PCR products were resolved on 1–2% agarose gels (Figs S8, S9).

### Time course silencing experiments

The time course experiments were conducted in three batches, each processing two KD replicates in parallel (batch A: replicates 1 and 2 of *ND7*-, *DevPF1*- and *DevPF2*-KD; batch B: replicates 3 and 4 of *ND7*-, *DevPF1*- and *DevPF2*-KD; batch C: replicates 5 and 6 of *ND7*- and *DevPF2*-KD). In batch A and B, cells were collected as soon as the first meiotic cells were observed in the population (onset), at between 20 and 40% fragmentation (early), at 80–90% fragmentation (late) and 6 h after the late time point (very late). In batch C, cells were collected before the onset of autogamy (vegetative), at 50% fragmentation (early), at 100% fragmentation plus with visible anlagen (very late) and 6 h later (very late plus 6 h). Given that batch C was collected at different stages, only the ‘very late’ time point of batch C was considered for differential expression analysis. For all time course replicates, enriched new MAC DNA was analyzed for IES retention and total RNA was collected from the collected time points for sRNA and/or mRNA analysis.

### Macronuclear isolation and Illumina DNA sequencing

Samples for new MAC isolation were collected from the KD cultures of all time course experiments 3 days after completion of sexual development as described previously (Arnaiz et al., 2012). DNA library preparation (350 bp fragment sizes) and Illumina sequencing (paired-end, 150 bp reads) were done by Novogene (UK) according to their standard protocols.

### IES retention and alternative boundary analysis

For IES retention score analysis, whole-genome sequencing reads of enriched new MAC DNA after KD were adaptor trimmed using TrimGalore (Krueger, 2019) if significant Illumina adapter content was observed using FastQC v0.11.9 (Andrews, 2010) (see Table S7 for adapter sequences). The ‘Map’ module of ParTIES v1.05 pipeline was used to map the reads on MAC and MAC+IES reference genomes with changes in the /lib/PARTIES/Map.pm file as described previously (Singh et al., 2023 preprint). The IES retention scores (IRS) were calculated by using the ‘MIRET’ module (provided as DevPF_IRS.tab.gz). IRS correlations were calculated as described previously (Swart et al., 2014).

Alternative excision was analyzed as described previously (Singh et al., 2023 preprint). In brief, properly paired and mapped reads were selected from the output from the ParTIES ‘Map’ module for the MAC+IES reference genome and downsampled to the same library size [DevPF1-KD (1) and DevPF2-KD (2) were excluded due to small library size]. We then employed the ‘MILORD’ module of a pre-release version of ParTIES (13 August 2015) with default parameters to annotate alternative and cryptic IES excision.

The data generate in this study was compared with data of previously published KDs: *PGM*-KD (Arnaiz et al., 2012), *TFIIS4*-KD (Maliszewska-Olejniczak et al., 2015), *SPT5m*-KD (Gruchota et al., 2017), *PTCAF1*-KD (Ignarski et al., 2014), *DCL2/3/5*-KD (Sandoval et al., 2014), *KU80c*-KD (Abello et al., 2020), *EZL1*-KD (Lhuillier-Akakpo et al., 2014) and *ISWI1*-KD (Singh et al., 2022).

### RNA extraction and sequencing

Total RNA was either extracted with phenol-chloroform followed by use of the Monarch Total RNA Miniprep kit (New England Biolabs) or a Quick-RNA Miniprep kit (Zymo). For phenol-chloroform extraction (batch C), 300 ml cells subjected to RNAi were washed twice with 10 mM Tris-HCl pH 7.5 (RT, 280 ***g***, 2 min) and shock frozen by dropping them directly into liquid nitrogen. 500 μl of 2× DNA/RNA protection reagent from the Monarch kit were added to the frozen pellet and the cells thawed by vortexing. After adding 10 μl proteinase K and 1 ml RNA lysis buffer, the manufacturer's instructions (RNA Binding and Elution, Cultured Mammalian Cells) were followed. On-column DNase I treatment was included.

For RNA extraction with Quick-RNA Miniprep kit (batch A and B), 100 ml of *Paramecium* cultures subjected to RNAi by feeding were washed twice in 10 mM Tris-HCl pH 7.5 in pear-shaped oil flasks by centrifugation (RT, 280 ***g***, 2 min). After the final wash, cells were collected on ice and spun at 2000 ***g*** for 2 min and 4°C and as much liquid as possible was removed. 3× volume of 1× DNA/RNA Shield (Biozym) was mixed with the cells and the samples were stored at −70°C until further processing. For RNA extraction, samples were thawed at RT and mixed with 1× volume of RNA lysis buffer. The manufacturer's instructions were followed (section: (III) Total RNA Purification).

Extracted total RNA was send to Azenta Life Sciences for library preparation (sRNA, NEBNext Small RNA Library Prep Set for Illumina; mRNA: NEBNext Ultra II RNA Library Prep Kit for Illumina) and paired-end Illumina sequencing (NovaSeq 2×150 bp).

### Small RNA analysis

Small RNA sequencing reads were trimmed using cutadapt (Martin, 2011) version 3.2 with the parameter -a ‘AGATCGGAAGAGCACACGTCTGAACTCCAGTCA’ to remove the relevant Illumina adaptor sequence. Trimmed reads were mapped to the *Paramecium tetraurelia* strain 51 MAC+IES genome and L4440 (*ND7*-KD) or T444 T (*DevPF1*/*DevPF2*-KD) silencing vector with bwa version 0.7.17-r1188 (Li and Durbin, 2009). GNU grep (version 2.14) was used to select 10–49 bp long, uniquely mapped reads (possessing the SAM file format flags “XT:A:U”) and sRNA length histograms were generated with an in-house Python script.

### mRNA analysis

Illumina adapter sequences (Table S7) were trimmed from reads with TrimGalore (Krueger, 2019). Reads were mapped to the *Paramecium tetraurelia* strain 51 transcriptome with hisat2 (Kim et al., 2019) allowing 20 multimappings (-k 20). Using samtools (Li et al., 2009), the properly paired and mapped reads were filtered (-f2 flag) and sorted by the read name (-n flag). Unique mapping reads were acquired with eXpress (Roberts and Pachter, 2013) with five additional online expectation-maximization rounds to perform on the data after the initial online round (-O 5 flag) to improve accuracy with in-house scripts.

Read counts were normalized with DEseq2 (Love et al., 2014) package in R software (version 3.6.3). For plotting, DEseq2 in-build functions plotPCA, plotMA and plotCounts were combined with ggplot2 (Villanueva and Chen, 2019) package (version 3.4.3). Differentially expressed genes were identified for each time point with a Wald test [false discovery rate (alpha)=0.1]. Differentially expressed genes were filtered with an absolute log2(Fold Change)>2 (corresponding to a 4-fold change) and an adjusted *P*<0.01. The time point, KD and batch were known sources of variation in the dataset (design=∼ batch+timepoint+KD+ timepoint:KD).

### Structure prediction with AlphaFold

Protein structures were predicted with AlphaFold2 multimer (Evans et al., 2021 preprint; Jumper et al., 2021) using the ColabFold v1.5.2-patch (Mirdita et al., 2022) in Google Colab with default parameters.

### Sequence alignment

Domains were predicted using InterProScan (Paysan-Lafosse et al., 2023). The nucleotide sequence of DevPF2 and DevPF1 (including introns) were aligned with clustalOmega (Sievers et al., 2011) (version 1.2.3) pairwise sequence alignment tool in Geneious prime (version 2023.2.1) with default parameters (Fig. 4A).

Multiple sequence alignment of PHD domains was done with clustalOmega (version 1.2.1) using the MPI bioinformatics toolkit's web interface (Zimmermann et al., 2018) with default parameters.

### Manuscript writing

Grammar and language refinement were assisted by an AI language model developed by OpenAI (GPT-3.5 architecture; https://chat.openai.com/chat) After using these services, the authors reviewed and edited the content as needed.

## Supplementary Material



10.1242/joces.261979_sup1 Supplementary information

## References

[JCS261979C1] Aasland, R., Gibson, T. J. and Stewart, A. F. (1995). The PHD finger: implications for chromatin-mediated transcriptional regulation. *Trends Biochem. Sci.* 20, 56-59. 10.1016/S0968-0004(00)88957-4 7701562

[JCS261979C2] Abello, A., Régnier, V., Arnaiz, O., Le Bars, R., Bétermier, M. and Bischerour, J. (2020). Functional diversification of Paramecium Ku80 paralogs safeguards genome integrity during precise programmed DNA elimination. *PLoS Genet.* 16, e1008723. 10.1371/journal.pgen.1008723 32298257 PMC7161955

[JCS261979C3] Allen, S. E., Hug, I., Pabian, S., Rzeszutek, I., Hoehener, C. and Nowacki, M. (2017). Circular concatemers of ultra-short DNA segments produce regulatory RNAs. *Cell* 168, 990-999.e7. 10.1016/j.cell.2017.02.020 28283070 PMC5346157

[JCS261979C4] Andrews, S. (2010). *FastQC: A Quality Control Tool for High Throughput Sequence Data*. Babraham Bioinformatics.

[JCS261979C5] Arnaiz, O., Mathy, N., Baudry, C., Malinsky, S., Aury, J.-M., Denby Wilkes, C., Garnier, O., Labadie, K., Lauderdale, B. E., Le Mouël, A. et al. (2012). The *Paramecium* germline genome provides a niche for intragenic parasitic DNA: evolutionary dynamics of internal eliminated sequences. *PLoS Genet.* 8, e1002984. 10.1371/journal.pgen.1002984 23071448 PMC3464196

[JCS261979C6] Arnaiz, O., Van Dijk, E., Bétermier, M., Lhuillier-Akakpo, M., de Vanssay, A., Duharcourt, S., Sallet, E., Gouzy, J. and Sperling, L. (2017). Improved methods and resources for *Paramecium* genomics: transcription units, gene annotation and gene expression. *BMC Genomics* 18, 483. 10.1186/s12864-017-3887-z 28651633 PMC5485702

[JCS261979C7] Bassing, C. H., Swat, W. and Alt, F. W. (2002). The mechanism and regulation of chromosomal V(D)J recombination. *Cell* 109, S45-S55. 10.1016/S0092-8674(02)00675-X 11983152

[JCS261979C8] Baudry, C., Malinsky, S., Restituito, M., Kapusta, A., Rosa, S., Meyer, E. and Bétermier, M. (2009). PiggyMac, a domesticated piggyBac transposase involved in programmed genome rearrangements in the ciliate Paramecium tetraurelia. *Genes Dev.* 23, 2478-2483. 10.1101/gad.547309 19884254 PMC2779751

[JCS261979C9] Bazin-Gélis, M., Eleftheriou, E., Zangarelli, C., Lelandais, G., Sperling, L., Arnaiz, O. and Bétermier, M. (2023). Inter-generational nuclear crosstalk links the control of gene expression to programmed genome rearrangements during the *Paramecium* sexual cycle. *Nucleic Acids Res*. 51, 12337-12351. 10.1093/nar/gkad1006 37953377 PMC10711438

[JCS261979C10] Beisson, J., Bétermier, M., Bré, M.-H., Cohen, J., Duharcourt, S., Duret, L., Kung, C., Malinsky, S., Meyer, E., Preer, J. R. et al. (2010a). Paramecium tetraurelia: the renaissance of an early unicellular model. *Cold Spring Harb. Protoc.* 2010, pdb.emo140. 10.1101/pdb.emo140 20150105

[JCS261979C11] Beisson, J., Bétermier, M., Bré, M.-H., Cohen, J., Duharcourt, S., Duret, L., Kung, C., Malinsky, S., Meyer, E., Preer, J. R. et al. (2010b). Maintaining clonal *Paramecium tetraurelia* cell lines of controlled age through daily reisolation. *Cold Spring Harb. Protoc.* 2010, pdb.prot5361. 10.1101/pdb.prot5361 20150120

[JCS261979C12] Beisson, J., Bétermier, M., Bré, M.-H., Cohen, J., Duharcourt, S., Duret, L., Kung, C., Malinsky, S., Meyer, E., Preer, J. R. et al. (2010c). Mass culture of *Paramecium tetraurelia*. *Cold Spring Harb. Protoc* 2010, pdb.prot5362. 10.1101/pdb.prot5362 20150121

[JCS261979C13] Beisson, J., Bétermier, M., Bré, M.-H., Cohen, J., Duharcourt, S., Duret, L., Kung, C., Malinsky, S., Meyer, E., Preer, J. R. et al. (2010d). DNA microinjection into the macronucleus of paramecium. *Cold Spring Harb. Protoc.* 2010, pdb.prot5364. 10.1101/pdb.prot5364 20150123

[JCS261979C14] Beisson, J., Bétermier, M., Bré, M.-H., Cohen, J., Duharcourt, S., Duret, L., Kung, C., Malinsky, S., Meyer, E., Preer, J. R. et al. (2010e). Silencing specific Paramecium tetraurelia genes by feeding double-stranded RNA. *Cold Spring Harb. Protoc.* 2010, pdb.prot5363. 10.1101/pdb.prot5363 20150122

[JCS261979C15] Berger, J. D. (1973). Nuclear differentiation and nucleic acid synthesis in well-fed exconjugants of Paramecium aurelia. *Chromosoma* 42, 247-268. 10.1007/BF00284774 4354261

[JCS261979C16] Betz, C., Schlenstedt, G. and Bailer, S. M. (2004). Asr1p, a novel yeast ring/PHD finger protein, signals alcohol stress to the nucleus. *J. Biol. Chem.* 279, 28174-28181. 10.1074/jbc.M401595200 15117954

[JCS261979C17] Bienz, M. (2006). The PHD finger, a nuclear protein-interaction domain. *Trends Biochem. Sci.* 31, 35-40. 10.1016/j.tibs.2005.11.001 16297627

[JCS261979C18] Black, J. C. and Kutateladze, T. G. (2023). Atypical histone targets of PHD fingers. *J. Biol. Chem.* 299, 104601. 10.1016/j.jbc.2023.104601 36907441 PMC10124903

[JCS261979C19] Bouhouche, K., Gout, J.-F., Kapusta, A., Bétermier, M. and Meyer, E. (2011). Functional specialization of Piwi proteins in *Paramecium tetraurelia* from post-transcriptional gene silencing to genome remodelling. *Nucleic Acids Res.* 39, 4249-4264. 10.1093/nar/gkq1283 21216825 PMC3105430

[JCS261979C20] Bozhenok, L., Wade, P. A. and Varga-Weisz, P. (2002). WSTF-ISWI chromatin remodeling complex targets heterochromatic replication foci. *EMBO J.* 21, 2231-2241. 10.1093/emboj/21.9.2231 11980720 PMC125993

[JCS261979C21] Chalker, D. L. (2012). Transformation and strain engineering of Tetrahymena. *Methods Cell Biol.* 109, 327-345. 10.1016/B978-0-12-385967-9.00011-6 22444150

[JCS261979C22] Chalker, D. L. and Yao, M. C. (2001). Nongenic, bidirectional transcription precedes and may promote developmental DNA deletion in Tetrahymena thermophila. *Genes Dev.* 15, 1287-1298. 10.1101/gad.884601 11358871 PMC313804

[JCS261979C23] Charmant, O., Gruchota, J., Arnaiz, O., Zangarelli, C., Bétermier, M., Nowak, K., Legros, V., Chevreux, G., Nowak, J. and Duharcourt, S. (2023). The nuclear PIWI-interacting protein Gtsf1 controls the selective degradation of small RNAs in *Paramecium*. *BioRxiv*, 10.1101/2023.09.19.558372. 10.1101/2023.09.19.558372 PMC11724296 39571614

[JCS261979C24] Cheng, C.-Y., Vogt, A., Mochizuki, K. and Yao, M.-C. (2010). A domesticated piggyBac transposase plays key roles in heterochromatin dynamics and DNA cleavage during programmed DNA deletion in Tetrahymena thermophila. *Mol. Biol. Cell* 21, 1753-1762. 10.1091/mbc.e09-12-1079 20357003 PMC2869380

[JCS261979C25] Dedukh, D. and Krasikova, A. (2022). Delete and survive: strategies of programmed genetic material elimination in eukaryotes. *Biol. Rev. Camb. Philos. Soc.* 97, 195-216. 10.1111/brv.12796 34542224 PMC9292451

[JCS261979C26] Deutsch, E. W., Bandeira, N., Perez-Riverol, Y., Sharma, V., Carver, J. J., Mendoza, L., Kundu, D. J., Wang, S., Bandla, C., Kamatchinathan, S. et al. (2023). The ProteomeXchange consortium at 10 years: 2023 update. *Nucleic Acids Res.* 51, D1539-D1548. 10.1093/nar/gkac1040 36370099 PMC9825490

[JCS261979C27] de Vanssay, A., Touzeau, A., Arnaiz, O., Frapporti, A., Phipps, J. and Duharcourt, S. (2020). The Paramecium histone chaperone Spt16-1 is required for Pgm endonuclease function in programmed genome rearrangements. *PLoS Genet.* 16, e1008949. 10.1371/journal.pgen.1008949 32702045 PMC7402521

[JCS261979C28] Driever, W. and Nüsslein-Volhard, C. (1988). A gradient of bicoid protein in Drosophila embryos. *Cell* 54, 83-93. 10.1016/0092-8674(88)90182-1 3383244

[JCS261979C29] Eberharter, A., Vetter, I., Ferreira, R. and Becker, P. B. (2004). ACF1 improves the effectiveness of nucleosome mobilization by ISWI through PHD-histone contacts. *EMBO J.* 23, 4029-4039. 10.1038/sj.emboj.7600382 15457208 PMC524333

[JCS261979C30] Evans, R., O'Neill, M., Pritzel, A., Antropova, N., Senior, A. W., Green, T., Žídek, A., Bates, R., Blackwell, S., Yim, J. et al. (2021). Protein complex prediction with AlphaFold-Multimer. *BioRxiv*, 10.1101/2021.10.04.463034. 10.1101/2021.10.04.463034

[JCS261979C31] Finn, R., Griffiths-Jones, S. and Bateman, A. (2003). Identifying protein domains with the Pfam database. *Curr. Protoc. Bioinformatics* 2, Unit 2.5. 10.1002/0471250953.bi0205s01 18428696

[JCS261979C32] Fleck, K., Nitz, M. and Jeffers, V. (2021). “Reading” a new chapter in protozoan parasite transcriptional regulation. *PLoS Pathog.* 17, e1010056. 10.1371/journal.ppat.1010056 34855919 PMC8638923

[JCS261979C33] Forment, J. V., Kaidi, A. and Jackson, S. P. (2012). Chromothripsis and cancer: causes and consequences of chromosome shattering. *Nat. Rev. Cancer* 12, 663-670. 10.1038/nrc3352 22972457

[JCS261979C34] Frapporti, A., Miró Pina, C., Arnaiz, O., Holoch, D., Kawaguchi, T., Humbert, A., Eleftheriou, E., Lombard, B., Loew, D., Sperling, L. et al. (2019). The Polycomb protein Ezl1 mediates H3K9 and H3K27 methylation to repress transposable elements in Paramecium. *Nat. Commun.* 10, 2710. 10.1038/s41467-019-10648-5 31221974 PMC6586856

[JCS261979C35] Furrer, D. I., Swart, E. C., Kraft, M. F., Sandoval, P. Y. and Nowacki, M. (2017). Two sets of piwi proteins are involved in distinct sRNA pathways leading to elimination of germline-specific DNA. *Cell Rep.* 20, 505-520. 10.1016/j.celrep.2017.06.050 28700949 PMC5522536

[JCS261979C36] Gaurav, N. and Kutateladze, T. G. (2023). Non-histone binding functions of PHD fingers. *Trends Biochem. Sci.* 48, 610-617. 10.1016/j.tibs.2023.03.005 37061424 PMC10330121

[JCS261979C37] Goday, C. and Pigozzi, M. I. (2010). Heterochromatin and histone modifications in the germline-restricted chromosome of the zebra finch undergoing elimination during spermatogenesis. *Chromosoma* 119, 325-336. 10.1007/s00412-010-0260-2 20217426

[JCS261979C38] Gozani, O., Karuman, P., Jones, D. R., Ivanov, D., Cha, J., Lugovskoy, A. A., Baird, C. L., Zhu, H., Field, S. J., Lessnick, S. L. et al. (2003). The PHD finger of the chromatin-associated protein ING2 functions as a nuclear phosphoinositide receptor. *Cell* 114, 99-111. 10.1016/S0092-8674(03)00480-X 12859901

[JCS261979C39] Grandchamp, S. and Beisson, J. (1981). Positional control of nuclear differentiation in paramecium. *Dev. Biol.* 81, 336-341. 10.1016/0012-1606(81)90297-9 7202844

[JCS261979C40] Gruchota, J., Denby Wilkes, C., Arnaiz, O., Sperling, L. and Nowak, J. K. (2017). A meiosis-specific Spt5 homolog involved in non-coding transcription. *Nucleic Acids Res.* 45, 4722-4732. 10.1093/nar/gkw1318 28053118 PMC5416832

[JCS261979C41] Guérin, F., Arnaiz, O., Boggetto, N., Denby Wilkes, C., Meyer, E., Sperling, L. and Duharcourt, S. (2017). Flow cytometry sorting of nuclei enables the first global characterization of *Paramecium* germline DNA and transposable elements. *BMC Genomics* 18, 327. 10.1186/s12864-017-3713-7 28446146 PMC5405496

[JCS261979C111] Guérineau, M., Bessa, L., Moriau, S., Lescop, E., Bontems, F., Mathy, N., Guittet, E., Bischerour., J., Bétermier, M., et al. (2021). The unusual structure of the PiggyMac cysteine-rich domain reveals zinc finger diversity in PiggyBac-related transposases. *Mob. DNA* 12, 12. 10.1186/s13100-021-00240-4 PMC8086355 33926516

[JCS261979C42] Hamilton, E. P., Kapusta, A., Huvos, P. E., Bidwell, S. L., Zafar, N., Tang, H., Hadjithomas, M., Krishnakumar, V., Badger, J. H., Caler, E. V. et al. (2016). Structure of the germline genome of *Tetrahymena thermophila* and relationship to the massively rearranged somatic genome. *Elife* 5, e19090. 10.7554/eLife.19090 27892853 PMC5182062

[JCS261979C43] Hoehener, C., Hug, I. and Nowacki, M. (2018). Dicer-like enzymes with sequence cleavage preferences. *Cell* 173, 234-247.e7. 10.1016/j.cell.2018.02.029 29551264 PMC5871716

[JCS261979C44] Howard-Till, R., Tian, M. and Loidl, J. (2019). A specialized condensin complex participates in somatic nuclear maturation in Tetrahymena thermophila. *Mol. Biol. Cell* 30, 1326-1338. 10.1091/mbc.E18-08-0487 30893010 PMC6724606

[JCS261979C45] Huber, W., von Heydebreck, A., Sültmann, H., Poustka, A. and Vingron, M. (2002). Variance stabilization applied to microarray data calibration and to the quantification of differential expression. *Bioinformatics* 18, S96-S104. 10.1093/bioinformatics/18.suppl_1.S96 12169536

[JCS261979C46] Ignarski, M., Singh, A., Swart, E. C., Arambasic, M., Sandoval, P. Y. and Nowacki, M. (2014). Paramecium tetraurelia chromatin assembly factor-1-like protein PtCAF-1 is involved in RNA-mediated control of DNA elimination. *Nucleic Acids Res.* 42, 11952-11964. 10.1093/nar/gku874 25270876 PMC4231744

[JCS261979C47] Jumper, J., Evans, R., Pritzel, A., Green, T., Figurnov, M., Ronneberger, O., Tunyasuvunakool, K., Bates, R., Žídek, A., Potapenko, A. et al. (2021). Highly accurate protein structure prediction with AlphaFold. *Nature* 596, 583-589. 10.1038/s41586-021-03819-2 34265844 PMC8371605

[JCS261979C48] Kim, D., Paggi, J. M., Park, C., Bennett, C. and Salzberg, S. L. (2019). Graph-based genome alignment and genotyping with HISAT2 and HISAT-genotype. *Nat. Biotechnol.* 37, 907-915. 10.1038/s41587-019-0201-4 31375807 PMC7605509

[JCS261979C49] Kinkelin, K., Wozniak, G. G., Rothbart, S. B., Lidschreiber, M., Strahl, B. D. and Cramer, P. (2013). Structures of RNA polymerase II complexes with Bye1, a chromatin-binding PHF3/DIDO homologue. *Proc. Natl. Acad. Sci. USA* 110, 15277-15282. 10.1073/pnas.1311010110 24003114 PMC3780847

[JCS261979C50] Klobutcher, L. A. and Herrick, G. (1995). Consensus inverted terminal repeat sequence of *Paramecium* IESs: resemblance to termini of Tc1-related and *Euplotes* Tec transposons. *Nucleic Acids Res.* 23, 2006-2013. 10.1093/nar/23.11.2006 7596830 PMC306976

[JCS261979C51] Kong, A. T., Leprevost, F. V., Avtonomov, D. M., Mellacheruvu, D. and Nesvizhskii, A. I. (2017). MSFragger: ultrafast and comprehensive peptide identification in mass spectrometry-based proteomics. *Nat. Methods* 14, 513-520. 10.1038/nmeth.4256 28394336 PMC5409104

[JCS261979C52] Krueger, F. (2019). *TrimGalore: A wrapper around Cutadapt and FastQC to consistently apply adapter and quality trimming to FastQ files, with extra functionality for RRBS data*. GitHub.

[JCS261979C53] Kuznetsov, D., Tegenfeldt, F., Manni, M., Seppey, M., Berkeley, M., Kriventseva, E. V. and Zdobnov, E. M. (2023). OrthoDB v11: annotation of orthologs in the widest sampling of organismal diversity. *Nucleic Acids Res.* 51, D445-D451. 10.1093/nar/gkac998 36350662 PMC9825584

[JCS261979C54] Lefort-Tran, M., Aufderheide, K., Pouphile, M., Rossignol, M. and Beisson, J. (1981). Control of exocytotic processes: cytological and physiological studies of trichocyst mutants in Paramecium tetraurelia. *J. Cell Biol.* 88, 301-311. 10.1083/jcb.88.2.301 7204496 PMC2111747

[JCS261979C55] Leinonen, R., Akhtar, R., Birney, E., Bower, L., Cerdeno-Tárraga, A., Cheng, Y., Cleland, I., Faruque, N., Goodgame, N., Gibson, R. et al. (2011). The european nucleotide archive. *Nucleic Acids Res.* 39, D28-D31. 10.1093/nar/gkq967 20972220 PMC3013801

[JCS261979C56] Lepère, G., Nowacki, M., Serrano, V., Gout, J.-F., Guglielmi, G., Duharcourt, S. and Meyer, E. (2009). Silencing-associated and meiosis-specific small RNA pathways in *Paramecium tetraurelia*. *Nucleic Acids Res.* 37, 903-915. 10.1093/nar/gkn1018 19103667 PMC2647294

[JCS261979C57] Lhuillier-Akakpo, M., Frapporti, A., Denby Wilkes, C., Matelot, M., Vervoort, M., Sperling, L. and Duharcourt, S. (2014). Local effect of enhancer of zeste-like reveals cooperation of epigenetic and cis-acting determinants for zygotic genome rearrangements. *PLoS Genet.* 10, e1004665. 10.1371/journal.pgen.1004665 25254958 PMC4177680

[JCS261979C58] Li, H. and Durbin, R. (2009). Fast and accurate short read alignment with Burrows-Wheeler transform. *Bioinformatics* 25, 1754-1760. 10.1093/bioinformatics/btp324 19451168 PMC2705234

[JCS261979C59] Li, H., Ilin, S., Wang, W., Duncan, E. M., Wysocka, J., Allis, C. D. and Patel, D. J. (2006). Molecular basis for site-specific read-out of histone H3K4me3 by the BPTF PHD finger of NURF. *Nature* 442, 91-95. 10.1038/nature04802 16728978 PMC4690523

[JCS261979C60] Li, H., Handsaker, B., Wysoker, A., Fennell, T., Ruan, J., Homer, N., Marth, G., Abecasis, G. and Durbin, R. and 1000 Genome Project Data Processing Subgroup. (2009). The sequence alignment/map format and SAMtools. *Bioinformatics* 25, 2078-2079. 10.1093/bioinformatics/btp352 19505943 PMC2723002

[JCS261979C61] Liu, Y., Taverna, S. D., Muratore, T. L., Shabanowitz, J., Hunt, D. F. and Allis, C. D. (2007). RNAi-dependent H3K27 methylation is required for heterochromatin formation and DNA elimination in Tetrahymena. *Genes Dev.* 21, 1530-1545. 10.1101/gad.1544207 17575054 PMC1891430

[JCS261979C62] Liu, L., Qin, S., Zhang, J., Ji, P., Shi, Y. and Wu, J. (2012). Solution structure of an atypical PHD finger in BRPF2 and its interaction with DNA. *J. Struct. Biol.* 180, 165-173. 10.1016/j.jsb.2012.06.014 22820306

[JCS261979C63] Love, M. I., Huber, W. and Anders, S. (2014). Moderated estimation of fold change and dispersion for RNA-seq data with DESeq2. *Genome Biol.* 15, 550. 10.1186/s13059-014-0550-8 25516281 PMC4302049

[JCS261979C64] Lu, X., Meng, X., Morris, C. A. and Keating, M. T. (1998). A novel human gene, WSTF, is deleted in Williams syndrome. *Genomics* 54, 241-249. 10.1006/geno.1998.5578 9828126

[JCS261979C65] Maliszewska-Olejniczak, K., Gruchota, J., Gromadka, R., Denby Wilkes, C., Arnaiz, O., Mathy, N., Duharcourt, S., Bétermier, M. and Nowak, J. K. (2015). TFIIS-dependent non-coding transcription regulates developmental genome rearrangements. *PLoS Genet.* 11, e1005383. 10.1371/journal.pgen.1005383 26177014 PMC4503560

[JCS261979C66] Mani, R.-S. and Chinnaiyan, A. M. (2010). Triggers for genomic rearrangements: insights into genomic, cellular and environmental influences. *Nat. Rev. Genet.* 11, 819-829. 10.1038/nrg2883 21045868

[JCS261979C67] Marmignon, A., Bischerour, J., Silve, A., Fojcik, C., Dubois, E., Arnaiz, O., Kapusta, A., Malinsky, S. and Bétermier, M. (2014). Ku-mediated coupling of DNA cleavage and repair during programmed genome rearrangements in the ciliate Paramecium tetraurelia. *PLoS Genet.* 10, e1004552. 10.1371/journal.pgen.1004552 25166013 PMC4148214

[JCS261979C68] Martin, M. (2011). Cutadapt removes adapter sequences from high-throughput sequencing reads. *EMBnet J.* 17, 10. 10.14806/ej.17.1.200

[JCS261979C69] Miller, J., McLachlan, A. D. and Klug, A. (1985). Repetitive zinc-binding domains in the protein transcription factor IIIA from Xenopus oocytes. *EMBO J.* 4, 1609-1614. 10.1002/j.1460-2075.1985.tb03825.x 4040853 PMC554390

[JCS261979C70] Mirdita, M., Schütze, K., Moriwaki, Y., Heo, L., Ovchinnikov, S. and Steinegger, M. (2022). ColabFold: making protein folding accessible to all. *Nat. Methods* 19, 679-682. 10.1038/s41592-022-01488-1 35637307 PMC9184281

[JCS261979C71] Miró-Pina, C., Charmant, O., Kawaguchi, T., Holoch, D., Michaud, A., Cohen, I., Humbert, A., Jaszczyszyn, Y., Chevreux, G., Del Maestro, L. et al. (2022). Paramecium Polycomb repressive complex 2 physically interacts with the small RNA-binding PIWI protein to repress transposable elements. *Dev. Cell* 57, 1037-1052.e8. 10.1016/j.devcel.2022.03.014 35429435

[JCS261979C72] Mochizuki, K., Fine, N. A., Fujisawa, T. and Gorovsky, M. A. (2002). Analysis of a piwi-related gene implicates small RNAs in genome rearrangement in tetrahymena. *Cell* 110, 689-699. 10.1016/S0092-8674(02)00909-1 12297043

[JCS261979C73] Morellet, N., Li, X., Wieninger, S. A., Taylor, J. L., Bischerour, J., Moriau, S., Lescop, E., Bardiaux, B., Mathy, N., Assrir, N. et al. (2018). Sequence-specific DNA binding activity of the cross-brace zinc finger motif of the piggyBac transposase. *Nucleic Acids Res.* 46, 2660-2677. 10.1093/nar/gky044 29385532 PMC5861402

[JCS261979C74] Morisato, D. and Anderson, K. V. (1995). Signaling pathways that establish the dorsal-ventral pattern of the Drosophila embryo. *Annu. Rev. Genet.* 29, 371-399. 10.1146/annurev.ge.29.120195.002103 8825480

[JCS261979C75] Nowacki, M., Zagorski-Ostoja, W. and Meyer, E. (2005). Nowa1p and Nowa2p: novel putative RNA binding proteins involved in trans-nuclear crosstalk in Paramecium tetraurelia. *Curr. Biol.* 15, 1616-1628. 10.1016/j.cub.2005.07.033 16169483

[JCS261979C76] Oppikofer, M., Sagolla, M., Haley, B., Zhang, H.-M., Kummerfeld, S. K., Sudhamsu, J., Flynn, E. M., Bai, T., Zhang, J., Ciferri, C. et al. (2017). Non-canonical reader modules of BAZ1A promote recovery from DNA damage. *Nat. Commun.* 8, 862. 10.1038/s41467-017-00866-0 29021563 PMC5636791

[JCS261979C77] Pascual, J., Martinez-Yamout, M., Dyson, H. J. and Wright, P. E. (2000). Structure of the PHD zinc finger from human Williams-Beuren syndrome transcription factor. *J. Mol. Biol.* 304, 723-729. 10.1006/jmbi.2000.4308 11124022

[JCS261979C78] Paysan-Lafosse, T., Blum, M., Chuguransky, S., Grego, T., Pinto, B. L., Salazar, G. A., Bileschi, M. L., Bork, P., Bridge, A., Colwell, L. et al. (2023). InterPro in 2022. *Nucleic Acids Res.* 51, D418-D427. 10.1093/nar/gkac993 36350672 PMC9825450

[JCS261979C79] Perez-Riverol, Y., Bai, J., Bandla, C., García-Seisdedos, D., Hewapathirana, S., Kamatchinathan, S., Kundu, D. J., Prakash, A., Frericks-Zipper, A., Eisenacher, M. et al. (2022). The PRIDE database resources in 2022: a hub for mass spectrometry-based proteomics evidences. *Nucleic Acids Res.* 50, D543-D552. 10.1093/nar/gkab1038 34723319 PMC8728295

[JCS261979C80] Pinskaya, M., Ghavi-Helm, Y., Mariotte-Labarre, S., Morillon, A., Soutourina, J. and Werner, M. (2014). PHD and TFIIS-Like domains of the Bye1 transcription factor determine its multivalent genomic distribution. *PLoS One* 9, e102464. 10.1371/journal.pone.0102464 25029256 PMC4100922

[JCS261979C81] Quan, J. and Tian, J. (2011). Circular polymerase extension cloning for high-throughput cloning of complex and combinatorial DNA libraries. *Nat. Protoc.* 6, 242-251. 10.1038/nprot.2010.181 21293463

[JCS261979C82] Ritchie, M. E., Phipson, B., Wu, D., Hu, Y., Law, C. W., Shi, W. and Smyth, G. K. (2015). limma powers differential expression analyses for RNA-sequencing and microarray studies. *Nucleic Acids Res.* 43, e47. 10.1093/nar/gkv007 25605792 PMC4402510

[JCS261979C83] Roberts, A. and Pachter, L. (2013). Streaming fragment assignment for real-time analysis of sequencing experiments. *Nat. Methods* 10, 71-73. 10.1038/nmeth.2251 23160280 PMC3880119

[JCS261979C84] Rooney, S., Chaudhuri, J. and Alt, F. W. (2004). The role of the non-homologous end-joining pathway in lymphocyte development. *Immunol. Rev.* 200, 115-131. 10.1111/j.0105-2896.2004.00165.x 15242400

[JCS261979C85] Roth, S., Stein, D. and Nüsslein-Volhard, C. (1989). A gradient of nuclear localization of the dorsal protein determines dorsoventral pattern in the Drosophila embryo. *Cell* 59, 1189-1202. 10.1016/0092-8674(89)90774-5 2688897

[JCS261979C86] Rzeszutek, I., Swart, E. C., Pabian-Jewuła, S., Russo, A. and Nowacki, M. (2022). Early developmental, meiosis-specific proteins - Spo11, Msh4-1, and Msh5 - Affect subsequent genome reorganization in Paramecium tetraurelia. *Biochim. Biophys. Acta Mol. Cell Res.* 1869, 119239. 10.1016/j.bbamcr.2022.119239 35181406

[JCS261979C87] Sanchez, R. and Zhou, M.-M. (2011). The PHD finger: a versatile epigenome reader. *Trends Biochem. Sci.* 36, 364-372. 10.1016/j.tibs.2011.03.005 21514168 PMC3130114

[JCS261979C88] Sandoval, P. Y., Swart, E. C., Arambasic, M. and Nowacki, M. (2014). Functional diversification of Dicer-like proteins and small RNAs required for genome sculpting. *Dev. Cell* 28, 174-188. 10.1016/j.devcel.2013.12.010 24439910

[JCS261979C89] Schindelin, J., Arganda-Carreras, I., Frise, E., Kaynig, V., Longair, M., Pietzsch, T., Preibisch, S., Rueden, C., Saalfeld, S., Schmid, B. et al. (2012). Fiji: an open-source platform for biological-image analysis. *Nat. Methods* 9, 676-682. 10.1038/nmeth.2019 22743772 PMC3855844

[JCS261979C90] Schindler, U., Beckmann, H. and Cashmore, A. R. (1993). HAT3.1, a novel Arabidopsis homeodomain protein containing a conserved cysteine-rich region. *Plant J.* 4, 137-150. 10.1046/j.1365-313X.1993.04010137.x 8106082

[JCS261979C91] Sellis, D., Guérin, F., Arnaiz, O., Pett, W., Lerat, E., Boggetto, N., Krenek, S., Berendonk, T., Couloux, A., Aury, J.-M. et al. (2021). Massive colonization of protein-coding exons by selfish genetic elements in *Paramecium* germline genomes. *PLoS Biol.* 19, e3001309. 10.1371/journal.pbio.3001309 34324490 PMC8354472

[JCS261979C92] Shieh, A. W. Y. and Chalker, D. L. (2013). LIA5 is required for nuclear reorganization and programmed DNA rearrangements occurring during tetrahymena macronuclear differentiation. *PLoS One* 8, e75337. 10.1371/journal.pone.0075337 24069402 PMC3775806

[JCS261979C93] Sievers, F., Wilm, A., Dineen, D., Gibson, T. J., Karplus, K., Li, W., Lopez, R., McWilliam, H., Remmert, M., Söding, J. et al. (2011). Fast, scalable generation of high-quality protein multiple sequence alignments using Clustal Omega. *Mol. Syst. Biol.* 7, 539. 10.1038/msb.2011.75 21988835 PMC3261699

[JCS261979C94] Singh, A., Maurer-Alcalá, X. X., Solberg, T., Häußermann, L., Gisler, S., Ignarski, M., Swart, E. C. and Nowacki, M. (2022). Chromatin remodeling is required for sRNA-guided DNA elimination in Paramecium. *EMBO J.* 41, e111839. 10.15252/embj.2022111839 36221862 PMC9670198

[JCS261979C95] Singh, A., Häußermann, L., Emmerich, C., Nischwitz, E., Seah, B. K., Butter, F. K. B., Nowacki, M. and Swart, E. C. (2023). ISWI1 complex proteins facilitate developmental genome editing in Paramecium. *BioRxiv*, 10.1101/2023.08.09.552620. 10.1101/2023.08.09.552620 PMC11789628 39542647

[JCS261979C96] Skene, P. J., Henikoff, J. G. and Henikoff, S. (2018). Targeted in situ genome-wide profiling with high efficiency for low cell numbers. *Nat. Protoc.* 13, 1006-1019. 10.1038/nprot.2018.015 29651053

[JCS261979C97] Spirov, A., Fahmy, K., Schneider, M., Frei, E., Noll, M. and Baumgartner, S. (2009). Formation of the bicoid morphogen gradient: an mRNA gradient dictates the protein gradient. *Development* 136, 605-614. 10.1242/dev.031195 19168676 PMC2685955

[JCS261979C98] Sturm, Á., Saskoi, É., Tibor, K., Weinhardt, N. and Vellai, T. (2018). Highly efficient RNAi and Cas9-based auto-cloning systems for C. elegans research. *Nucleic Acids Res.* 46, e105. 10.1093/nar/gky516 29924347 PMC6158509

[JCS261979C99] Swart, E. C., Wilkes, C. D., Sandoval, P. Y., Arambasic, M., Sperling, L. and Nowacki, M. (2014). Genome-wide analysis of genetic and epigenetic control of programmed DNA deletion. *Nucleic Acids Res.* 42, 8970-8983. 10.1093/nar/gku619 25016527 PMC4132734

[JCS261979C100] Taverna, S. D., Coyne, R. S. and Allis, C. D. (2002). Methylation of histone h3 at lysine 9 targets programmed DNA elimination in tetrahymena. *Cell* 110, 701-711. 10.1016/S0092-8674(02)00941-8 12297044

[JCS261979C101] Villanueva, R. A. M. and Chen, Z. J. (2019). ggplot2: elegant graphics for data analysis (2nd ed.). *Meas. Interdiscip. Res. Perspect.* 17, 160-167. 10.1080/15366367.2019.1565254

[JCS261979C102] Wang, J. and Davis, R. E. (2014). Programmed DNA elimination in multicellular organisms. *Curr. Opin. Genet. Dev.* 27, 26-34. 10.1016/j.gde.2014.03.012 24886889 PMC4125452

[JCS261979C103] Wang, C., Solberg, T., Maurer-Alcalá, X. X., Swart, E. C., Gao, F. and Nowacki, M. (2022). A small RNA-guided PRC2 complex eliminates DNA as an extreme form of transposon silencing. *Cell Rep.* 40, 111263. 10.1016/j.celrep.2022.111263 36001962 PMC10073204

[JCS261979C104] Wang, C., Lv, L., Solberg, T., Wen, Z., Zhang, H. and Gao, F. (2023). Conservation of the ancestral function of GTSF1 in transposon silencing in the unicellular eukaryote *Paramecium tetraurelia*. *BioRxiv*, 10.1101/2023.10.06.561219. 10.1101/2023.10.06.561219

[JCS261979C105] Wysocka, J., Swigut, T., Xiao, H., Milne, T. A., Kwon, S. Y., Landry, J., Kauer, M., Tackett, A. J., Chait, B. T., Badenhorst, P. et al. (2006). A PHD finger of NURF couples histone H3 lysine 4 trimethylation with chromatin remodelling. *Nature* 442, 86-90. 10.1038/nature04815 16728976

[JCS261979C106] Zangarelli, C., Arnaiz, O., Bourge, M., Gorrichon, K., Jaszczyszyn, Y., Mathy, N., Escoriza, L., Bétermier, M. and Régnier, V. (2022). Developmental timing of programmed DNA elimination in Paramecium tetraurelia recapitulates germline transposon evolutionary dynamics. *Genome Res.* 32, 2028-2042. 10.1101/gr.277027.122 36418061 PMC9808624

[JCS261979C107] Zeng, L., Zhang, Q., Li, S., Plotnikov, A. N., Walsh, M. J. and Zhou, M.-M. (2010). Mechanism and regulation of acetylated histone binding by the tandem PHD finger of DPF3b. *Nature* 466, 258-262. 10.1038/nature09139 20613843 PMC2901902

[JCS261979C108] Zhang, J., Yan, G., Tian, M., Ma, Y., Xiong, J. and Miao, W. (2018). A DP-like transcription factor protein interacts with E2fl1 to regulate meiosis in Tetrahymena thermophila. *Cell Cycle* 17, 634-642. 10.1080/15384101.2018.1431595 29417875 PMC5969552

[JCS261979C109] Zhang, F., Bechara, S. and Nowacki, M. (2024). Structural maintenance of chromosomes (SMC) proteins are required for DNA elimination in Paramecium. *Life Sci. Alliance* 7, e202302281. 10.26508/lsa.202302281 38056908 PMC10700549

[JCS261979C110] Zimmermann, L., Stephens, A., Nam, S.-Z., Rau, D., Kübler, J., Lozajic, M., Gabler, F., Söding, J., Lupas, A. N. and Alva, V. (2018). A completely reimplemented MPI bioinformatics toolkit with a new hhpred server at its core. *J. Mol. Biol.* 430, 2237-2243. 10.1016/j.jmb.2017.12.007 29258817

