# Supplementary material for: Two paralogous PHD finger proteins participate in natural genome editing in Paramecium tetraurelia
Source: J Cell Sci. 2024 Aug 30;137(16):jcs261979. doi: 10.1242/jcs.261979 (PMC11385659; doi:10.1242/jcs.261979)
Supplement: Supplementary information [file joces-137-261979-s1.pdf]

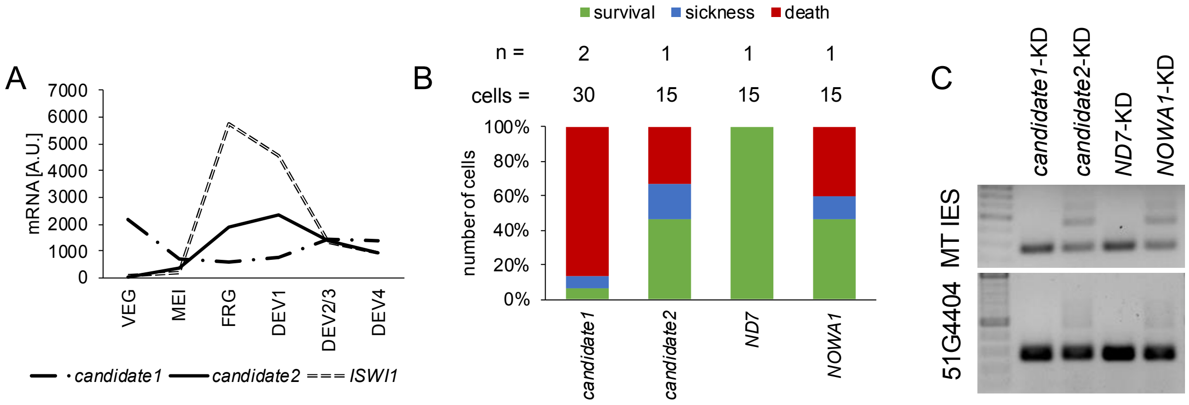

Fig. S1. Screening for new candidate genes involved in *Paramecium* genome reorganization

(A) mRNA expression profiles in arbitrary units for candidate 1, candidate 2 and *ISWI1* during various developmental stages: VEG (vegetative growth), MEI (micronuclear meiosis and macronuclear fragmentation), FRG (~50% of the population with fragmented maternal MACs), DEV1 (significant proportion with visible anlagen), DEV2/3 (majority with visible anlagen), DEV4 (majority with visible anlagen). Expression data retrieved from ParameciumDB (Arnaiz et al., 2017). (B) Viability of new progeny after knockdowns (*ND7* (negative control) and *NOWA1* (positive control) compared to two candidate genes). Survival: no growth defects. Sickness: reduced division rate. Death: 3 or less cells after three days. The numbers of experiments (n) and cells counted (cells) are indicated at the top. (C) IES retention PCRs for two IESs on genomic DNA isolated from knockdown cells.

Journal of Cell Science • Supplementary information

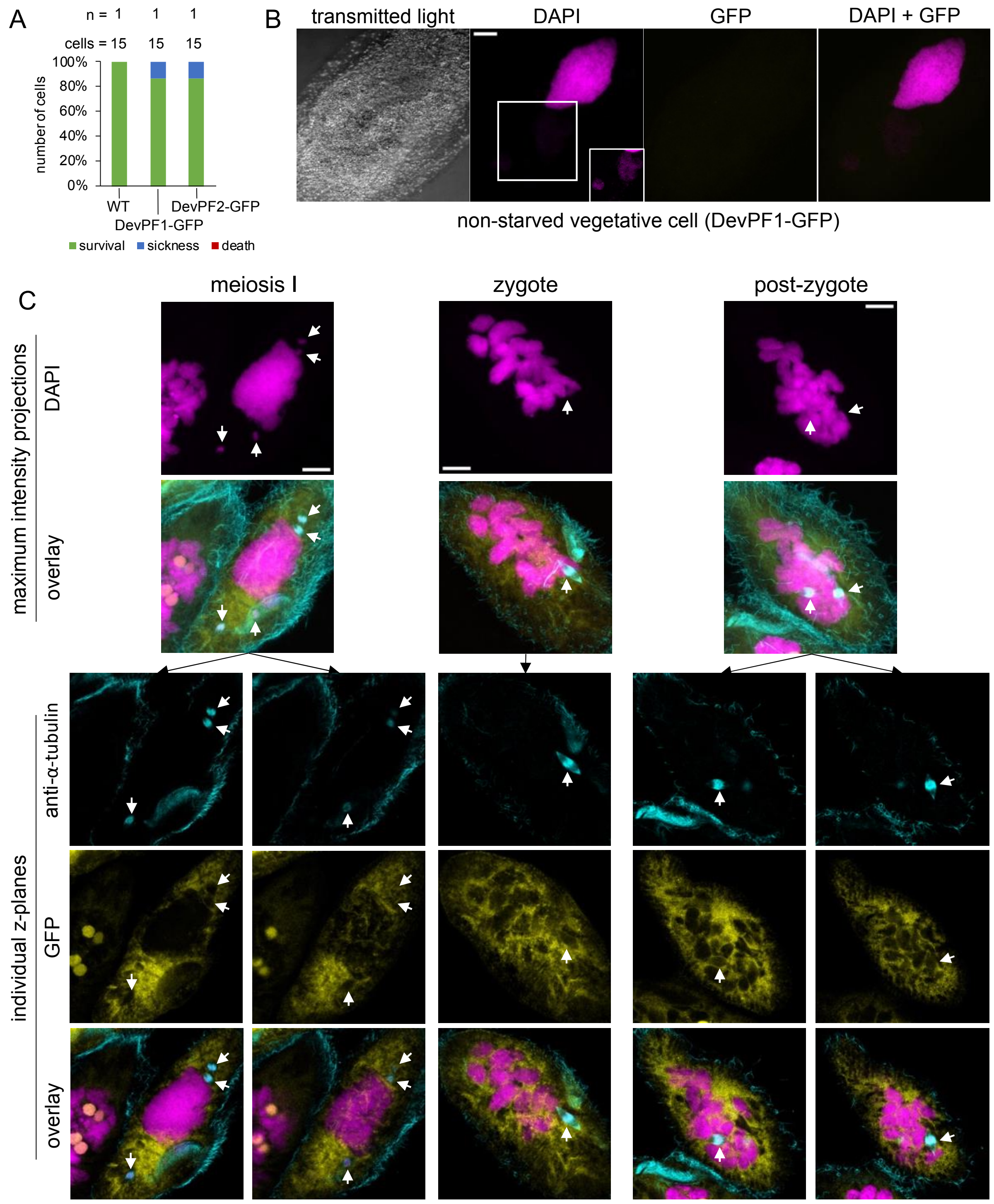

Fig. S2. Additional imaging of DevPF1-GFP localization

(A) Viability of cells injected with GFP-fusion proteins compared to non-transformed wild type (WT). Survival: normal division. Sickness: reduced growth. Death: 3 or less cells after three days. The numbers of experiments (n) and cells counted (cells) are indicated at the top. (B) DevPF1-GFP localization in non-starved vegetative cells with bacteria inside food vacuoles. (C) Gametic and post-zygotic nuclei lacking DevPF1-GFP localization. Maximum intensity projections of multiple confocal z-planes for (B) and (C) and individual confocal planes for (C). (B) and (C): DNA (stained with DAPI) in pink. GFP in yellow. Scale bar = 10 µm.

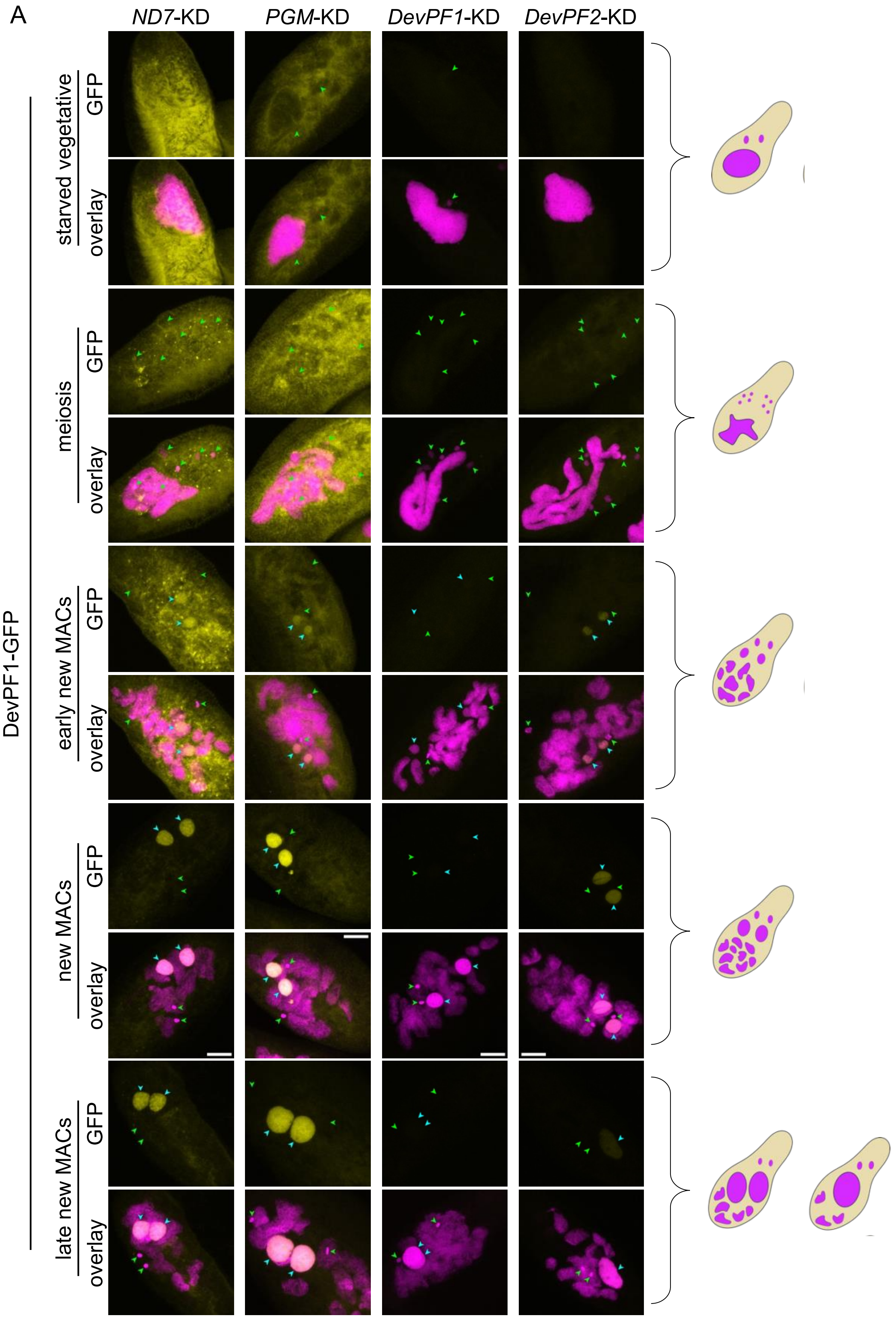

Journal of Cell Science • Supplementary information

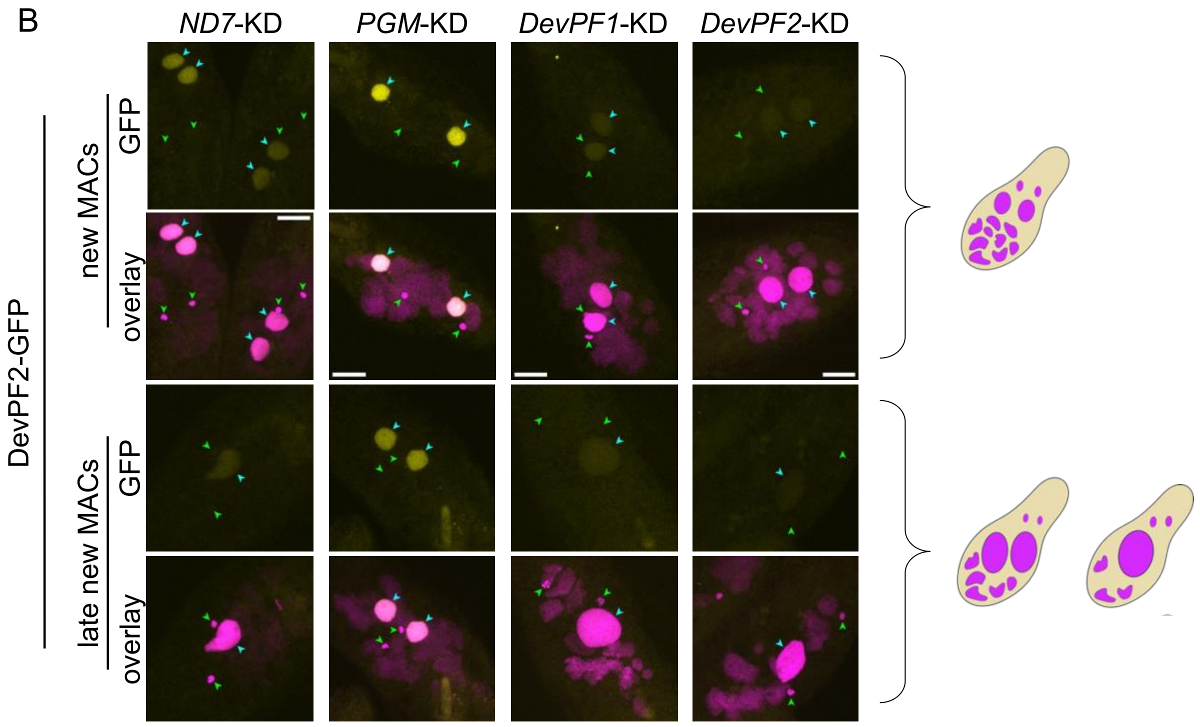

Fig. S3. Localization of DevPF-GFP proteins upon gene knockdowns DevPF1-GFP (A) and DevPF2-GFP (B) localization upon *ND7*, *PGM*, *DevPF1* and *DevPF2* knockdown. GFP signal (yellow) and DNA stained with DAPI (pink). Scale bar = 10 μm. Schematic representation of the corresponding cell stage (right).

Journal of Cell Science • Supplementary information

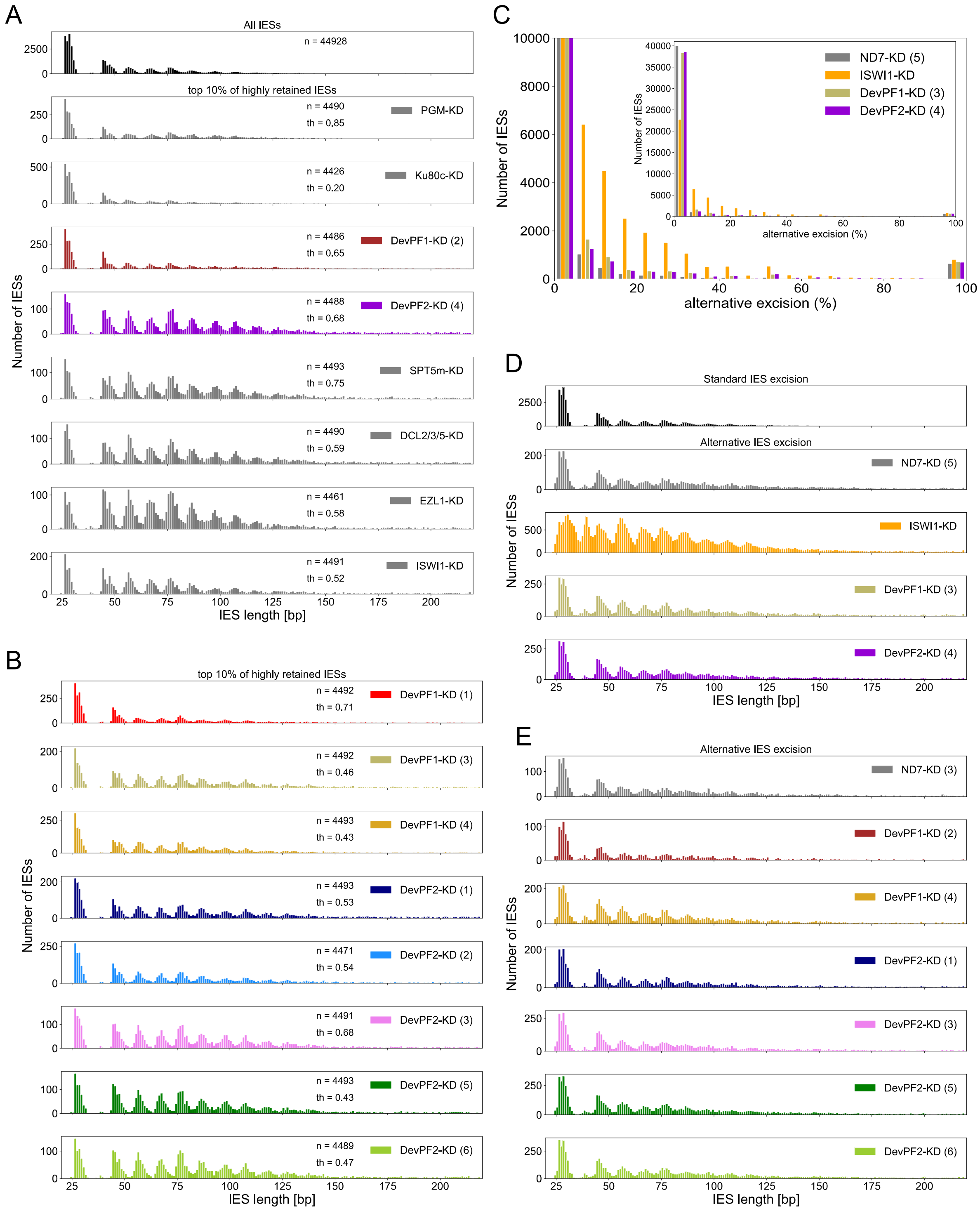

Fig. S4. Analysis of length distributions of standard and alternatively excised IESs upon gene knockdown

(A) and (B): Length distribution of the top 10% most highly retained IESs in different knockdowns (A) and *DevPF* replicates (B). Indicated are the number of IESs (n) and the IRS threshold (th). (C) Histogram showing the fraction of alternative excision (%) for each IES in various knockdowns. *ND7:* negative control, *ISWI1*: positive control. (D) and (E): Length distribution of alternatively excised IES in different knockdowns (D) and *DevPF* replicates (E).

Journal of Cell Science • Supplementary information

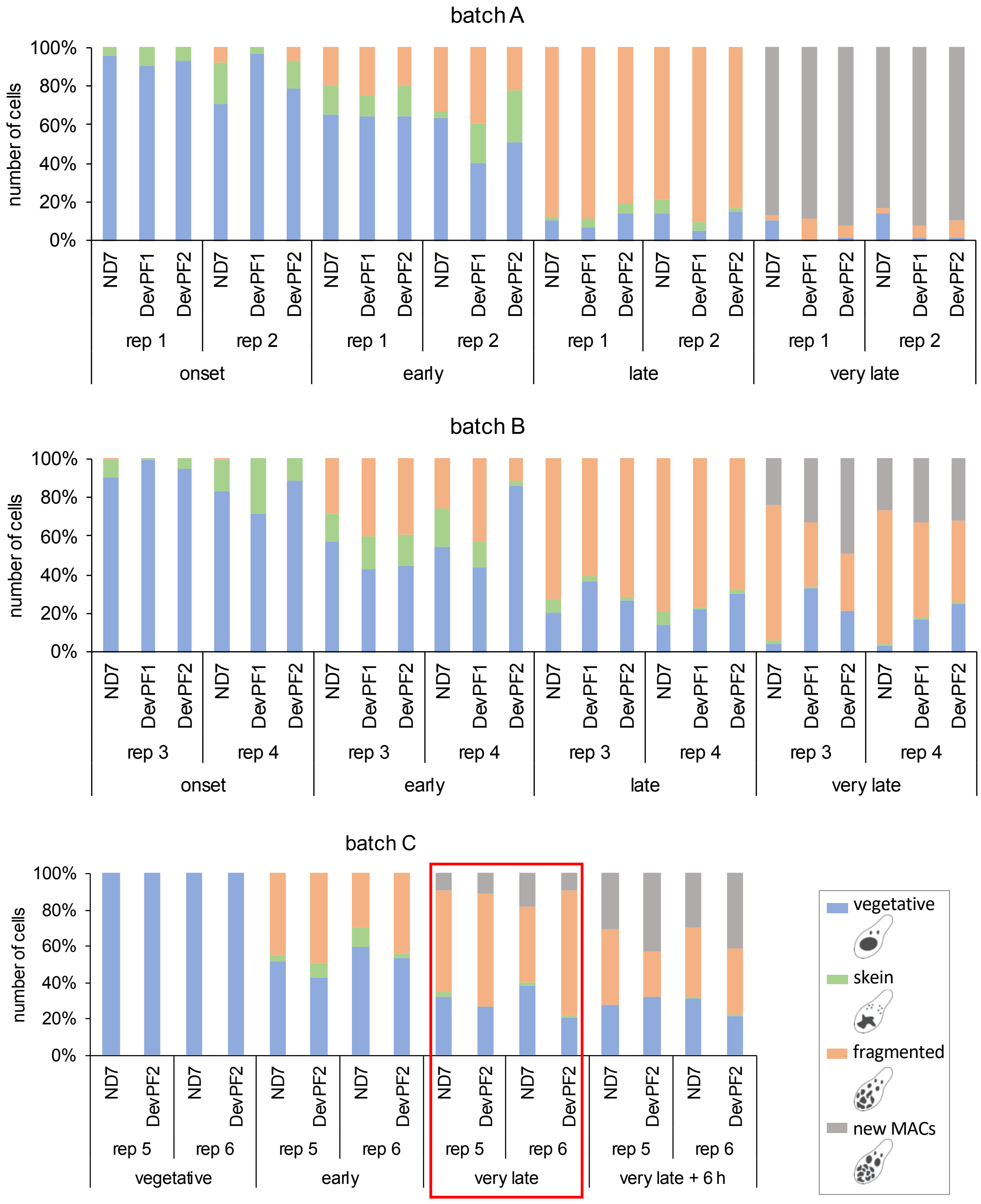

Fig. S5. Cell staging for gene knockdown time course experiments

Cell staging of 6 time series conducted in 3 batches. A and B included *ND7*-KD, *DevPF1*-KD and *DevPF2*-KD, while C included *ND7*-KD and *DevPF2*-KD. All replicates and time points of batch A and B and "very late" time point of batch C were used for mRNA differential expression analysis. Cells with visible new MACs were considered in the "very late" and "very late + 6 h" time points. The schematic representation of the considered cell stages is provided.

Journal of Cell Science • Supplementary information

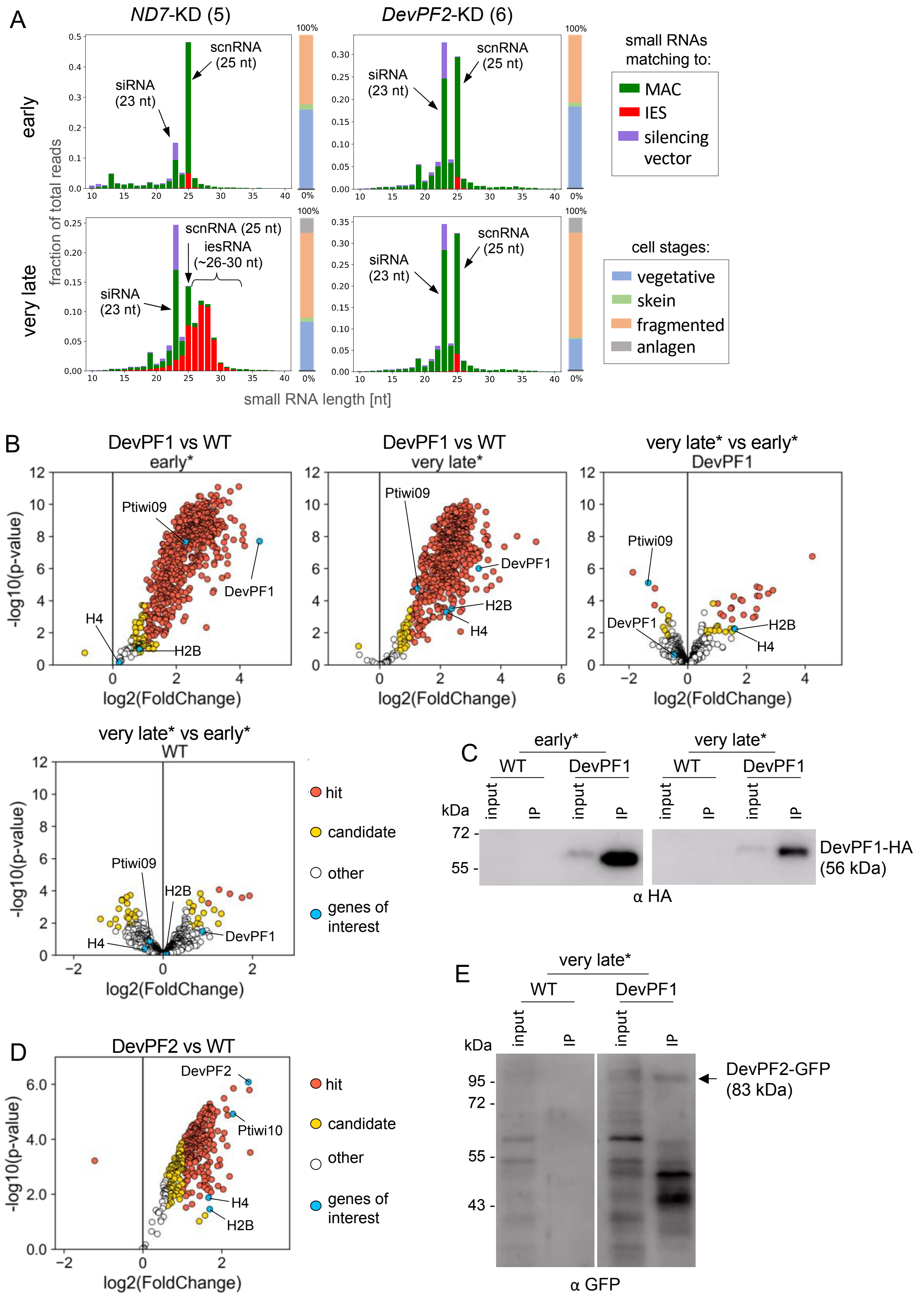

Journal of Cell Science • Supplementary information

Fig. S6. Small RNA populations and potential DevPF interaction partners

(A) Small RNA populations (10-40 nt) at “early” and “very late” time points in different knockdowns (*ND7* (control) and *DevPF2*) mapping to silencing plasmid backbone (vector), MAC or IES sequences. Individual cell stage compositions are indicated in the bar to the right of each diagram, along with schematic representations of the cell stages considered. (B) and (D): Genes identified by mass spectrometry in DevPF1-HA (B) or DevPF2-GFP (D) immunoprecipitations (IP). Non-transformed wildtype cells (WT) as control. “hit”: false discovery rate (FDR) smaller 5% and a fold-change (FC) of at least 100%; “candidate”: FDR below 20% and a FC of at least 50%. Early (about 30% fragmentation) in (B) and very late (100% fragmentation + visible new MACs) time points in (B) and (D) were collected. For DevPF1-IP (B), multiple comparisons are shown. Above each subgraph the comparison between samples is indicated, with the common condition just below. Genes of interest are labeled with one name in cases where the detected peptides cannot be unambiguously distinguished. (C) and (E): Western blots of input and IP fraction from IPs performed on DevPF1-HA (HA-affinity IP) (E) and DevPF2-GFP (GFP-affinity IP) (F) with wildtype (WT) as control. (*): These time points are not represented in the cell stage compositions in Fig S5.

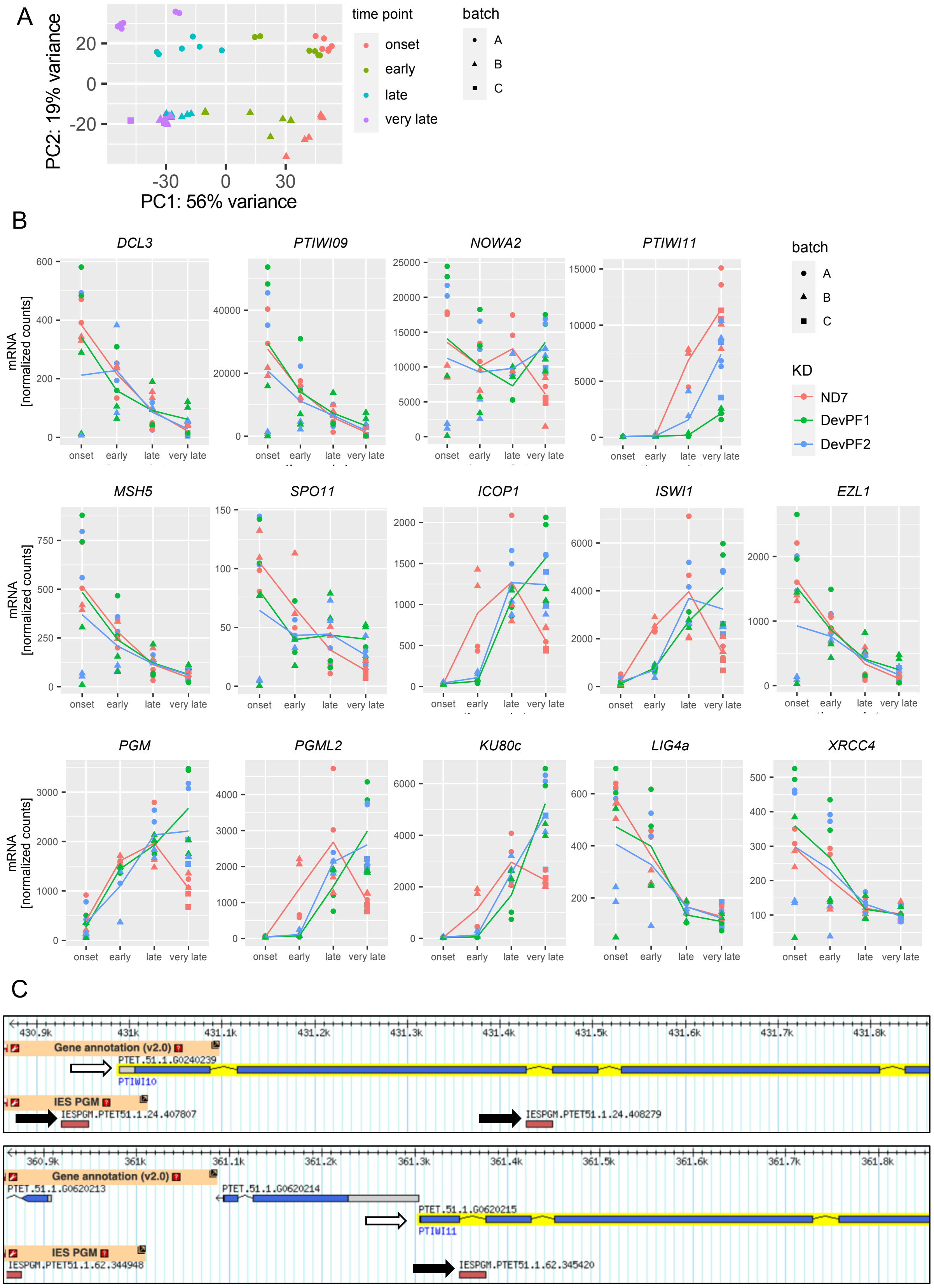

Fig. S7. Differential expression of genes involved in *Paramecium* genome reorganization upon *DevPF* knockdowns

(A) Principal Component Analysis (PCA) for mRNA-seq samples. (B) Gene expression levels of selected genes upon knockdowns (*ND7* (control), *DevPF1* and *DevPF2*) at developmental time points (onset, early, late and very late). The lines represent the mean of all replicates in a given knockdown and time point. (C) Screenshot from GBrowse tool on ParameciumDB showing IESs in *PTIWI10* (top) and *PTIWI11* (bottom) flanking/coding regions. White arrow: start of the gene. Black arrow: IES.

Journal of Cell Science • Supplementary information

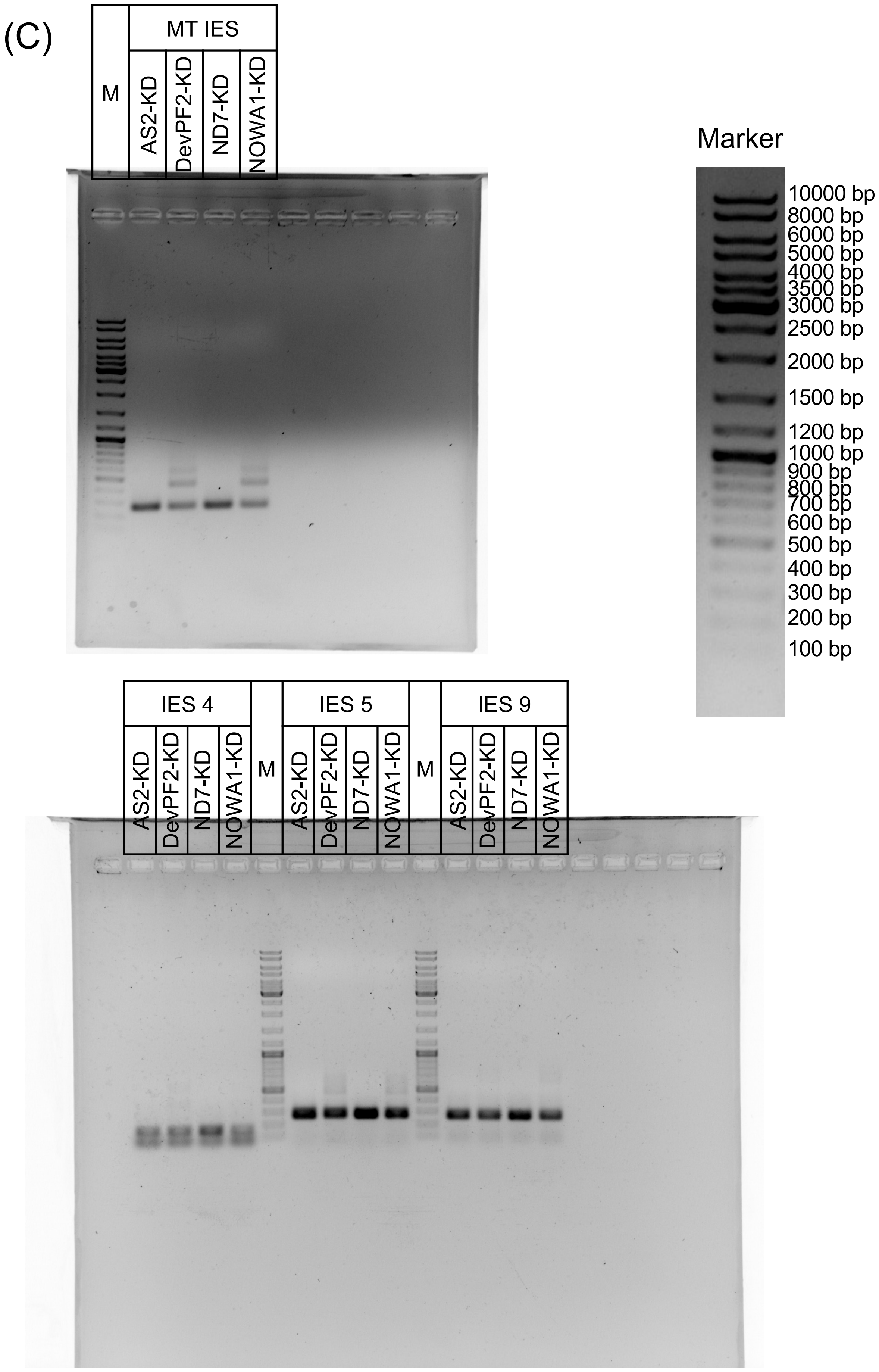

Fig. S8. Blot Transparency. Full inverted gel images for Fig. S1C

Journal of Cell Science • Supplementary information

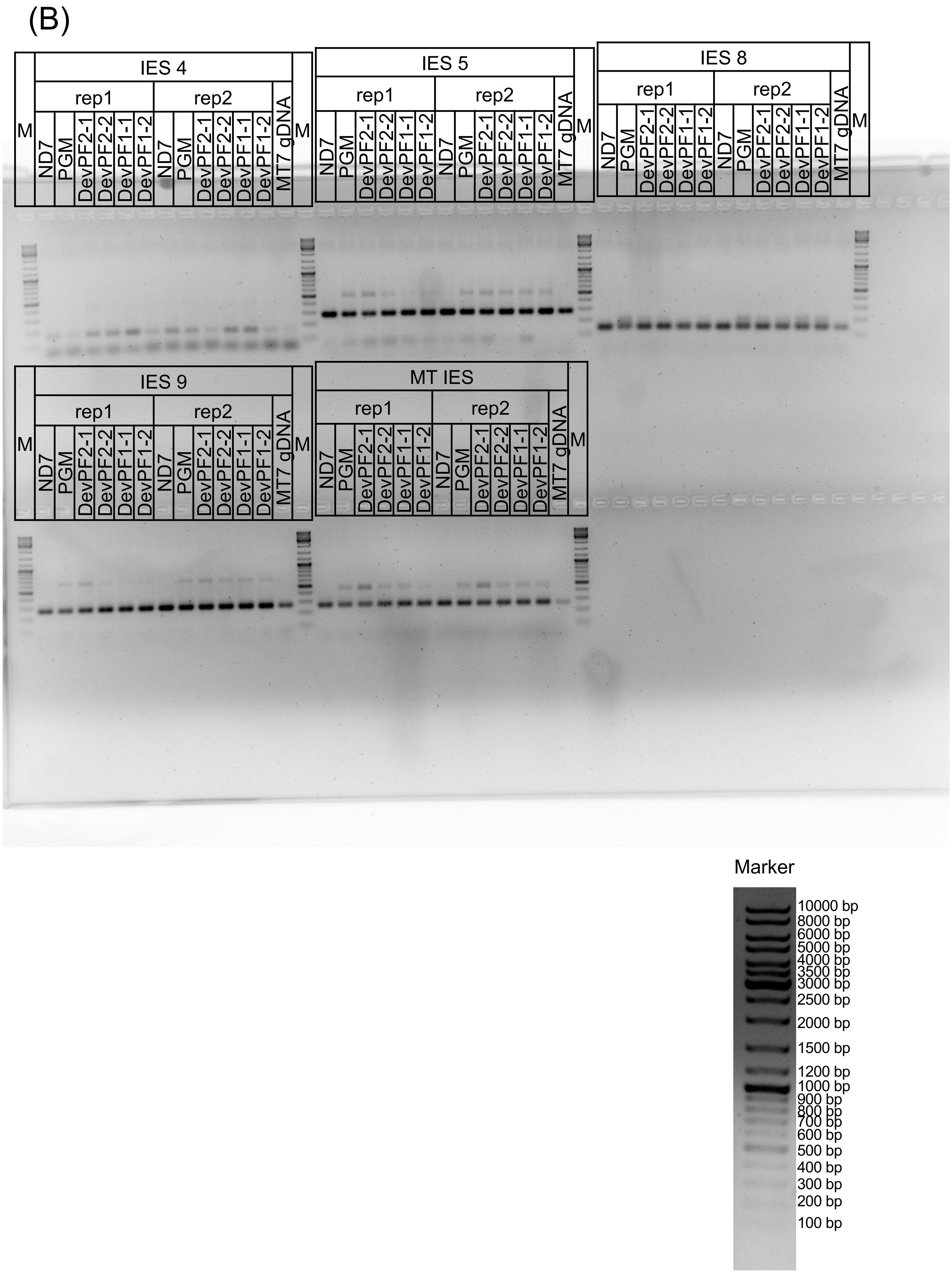

Fig. S9. Full inverted gel images for Fig. 5B

early time point (30% fragmentation)

(C)

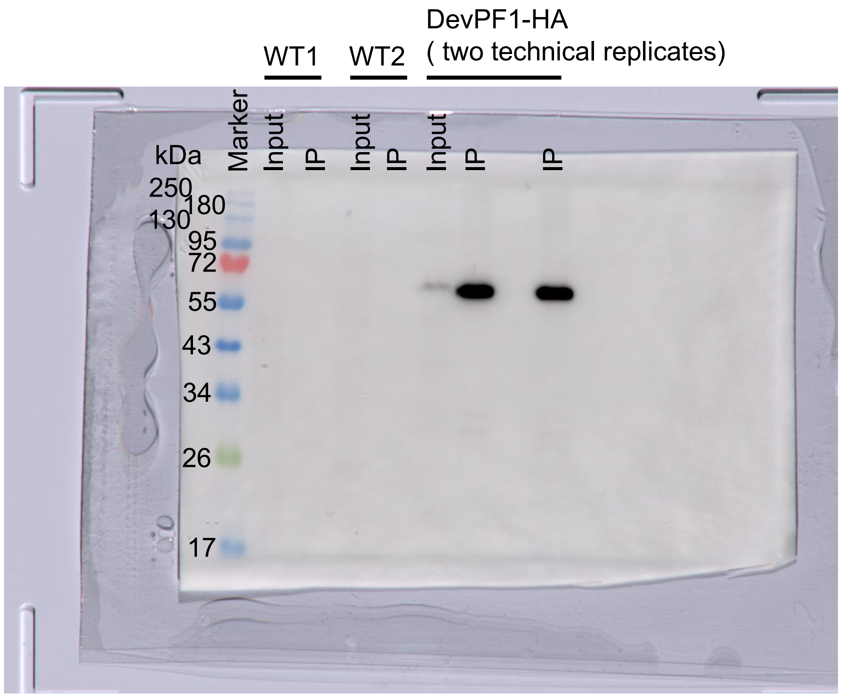

10µl of total lysate was loaded as Input, 10µl of elution (50µl total) was loaded as IP.
1:500 of HRP conjugated HA (Santa Cruz) was incubated overnight at 4°C. 13s exposure

late time point (100% fragmentation+Anlagen)

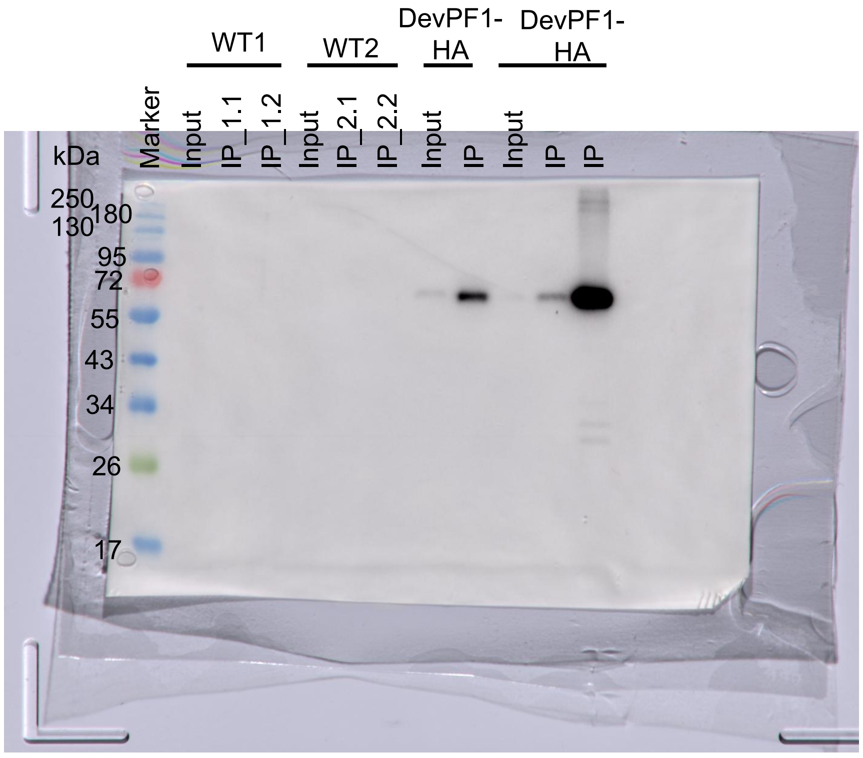

10µl of total lysate was loaded as Input, 10µl of elution (50µl total) was loaded as IP.
1:500 of HRP conjugated HA (Santa Cruz) was incubated overnight at 4°C. 7s exposure

Fig. S10. Full blot images for Fig. S6C

Journal of Cell Science • Supplementary information

(E)

late time point (100% fragmentation+Anlagen)

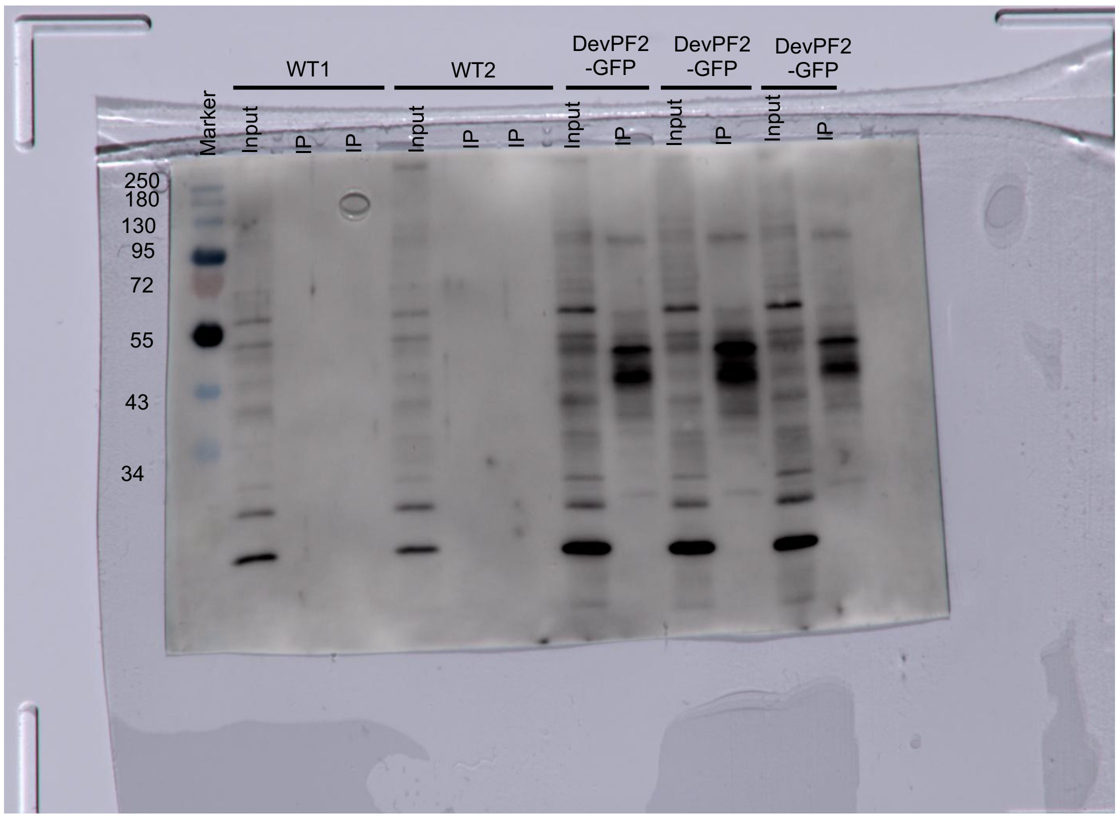

Fig. S11. Full blot image for Fig. S6E

Journal of Cell Science • Supplementary information

Table S1. Lengths of the most highly retained IESs

For each KD, the median and mean length [bp] of the top 10% most highly retained IESs are provided.

Knockdown	Median [bp]	Mean [bp]
all IESs	50	79.1
PGM-KD	67	130.0
KU80c-KD	45	86.8
DevPF1-KD (2)	66	143.9
DevPF2-KD (4)	84.5	134.8
SPT5m-KD	85	179.4
DCL2/3/5-KD	85	153.2
EZL1-KD	77	145.7
ISWI1-KD	77	148.6
DevPF1-KD (1)	62	124.9
DevPF1-KD (3)	85	184.1
DevPF1-KD (4)	75	160.4
DevPF2-KD (1)	86	166.9
DevPF2-KD (2)	76	141.1
DevPF2-KD (3)	81	130.0
DevPF2-KD (5)	79	133.9
DevPF2-KD (6)	85	143.6

Journal of Cell Science • Supplementary information

Table S2. Statistics alternative excision

For each KD, the percentage of alternative excision events was determined for each IES.The median and mean values for all IESs are provided. Replicates are indicated in parentheses. Rep = replicates.

Knockdown	Rep	median [%]	mean [%]
ND7	3	0	2.72
	4	0	2.43
ISWI1	-	4.35	10.88
DevPF1	2	0	2.53
	3	0	3.82
	4	0	3.49
DevPF2	1	0	3.54
	3	0	3.85
	4	0	3.96
	5	0	3.52
	6	0	3.53

Journal of Cell Science • Supplementary information

Table S3. Protein abundance in DevPF1-IP

Selected proteins identified by mass spectrometry in DevPF1- and DevPF2-IP. Comparison: the compared samples and, if applicable, the common condition. Protein: Ambiguous hits are labeled with one name. “hit”: false discovery rate (FDR) smaller 5% and a fold-change (FC) of at least 100%; “candidate”: FDR below 20% and a FC of at least 50%.

Comparison	Protein	LogFC	Log(p)	Hit annotation
DevPF1 vs WT (early)	DevPF1	4.61	7.69	hit
	Ptiwi01	2.29	7.67	hit
	Histone 2B	0.84	0.97	candidate
	Histone 4	0.20	0.16	no hit
DevPF1 vs WT (late)	DevPF1	3.26	6.00	hit
	Ptiwi01	1.25	4.77	hit
	Histone 2B	2.37	3.49	hit
	Histone 4	2.20	3.29	hit
late vs early (DevPF1)	DevPF1	-0.47	0.64	no hit
	Ptiwi01	-1.35	5.11	hit
	Histone 2B	1.60	2.23	candidate
	Histone 4	1.59	2.26	candidate
late vs early (WT)	DevPF1	0.89	1.48	no hit
	Ptiwi01	-0.30	0.88	no hit
	Histone 2B	0.07	0.05	no hit
	Histone 4	-0.42	0.40	no hit
DevPF2 vs WT	DevPF2	2.67	6.08	hit
	Ptiwi10	2.28	4.92	hit
	Histone 2B	1.70	1.45	hit
	Histone 4	1.67	1.88	hit

Table S4. Differential expression of selected genes (p-value)

Adjusted p-values for differential expression (thresholds: |log2(Fold Change)| > 2; adjusted p-value < 0.01) of selected genes in different knockdown comparisons and time points. Magenta = upregulation; Blue = downregulation.

	DevPF1-KD vs *ND7*-KD				*DevPF2*-KD vs *ND7*-KD				*DevPF2*-KD vs *DevPF1*-KD			
Gene	onset	early	late	very late	onset	early	late	very late	onset	early	late	very late
DCL2	9.97E-01	1.00E+00	9.80E-01	6.60E-01	6.25E-01	1.00E+00	9.87E-01	9.85E-01	6.09E-01	1.00E+00	1.00E+00	1.00E+00
DCL3	9.96E-01	1.00E+00	9.99E-01	3.93E-01	6.85E-01	1.00E+00	9.97E-01	8.19E-01	7.26E-01	1.00E+00	1.00E+00	1.00E+00
DCL5	9.74E-01	9.87E-07	1.23E-01	1.97E-01	6.23E-01	7.36E-06	3.02E-01	6.29E-01	8.40E-01	1.00E+00	1.00E+00	1.00E+00
DevPF1	1.14E-02	6.41E-04	1.15E-01	5.81E-01	1.54E-01	1.00E+00	8.51E-01	9.61E-01	1.80E-01	8.07E-01	1.00E+00	1.00E+00
DevPF2	9.83E-01	6.44E-01	4.61E-02	6.63E-01	8.10E-01	2.36E-07	4.51E-08	5.54E-04	9.71E-01	2.25E-01	1.00E+00	4.75E-01
EZL1	9.97E-01	1.00E+00	9.20E-01	4.47E-01	6.19E-01	1.00E+00	9.53E-01	7.08E-01	6.53E-01	1.00E+00	1.00E+00	1.00E+00
GTSF1	9.93E-01	1.00E+00	9.16E-01	5.10E-01	7.25E-01	1.00E+00	9.64E-01	7.54E-01	8.26E-01	1.00E+00	1.00E+00	1.00E+00
ICOP1	9.94E-01	1.87E-20	8.06E-01	2.52E-03	6.28E-01	3.39E-13	9.97E-01	5.26E-03	5.83E-01	1.00E+00	1.00E+00	1.00E+00
ICOP2	8.98E-01	5.32E-25	9.70E-01	1.28E-01	3.22E-01	6.03E-14	7.81E-01	1.34E-02	8.69E-01	1.00E+00	1.00E+00	1.00E+00
ISWI1	8.16E-01	1.08E-03	7.46E-01	1.48E-02	9.13E-01	1.07E-04	9.90E-01	9.36E-03	3.43E-01	1.00E+00	1.00E+00	1.00E+00
KU80c	9.09E-01	1.44E-19	2.89E-01	5.67E-02	6.37E-01	8.69E-11	9.14E-01	4.30E-02	2.32E-01	1.00E+00	1.00E+00	1.00E+00
LIG4a	9.37E-01	1.00E+00	8.45E-01	8.04E-01	3.82E-01	1.00E+00	9.96E-01	9.92E-01	7.68E-01	1.00E+00	1.00E+00	1.00E+00
MSH4a	9.84E-01	1.00E+00	9.13E-01	7.58E-01	4.85E-01	1.00E+00	9.62E-01	9.82E-01	6.50E-01	1.00E+00	1.00E+00	1.00E+00
MSH4b	9.96E-01	1.00E+00	6.50E-01	4.96E-01	4.89E-01	1.00E+00	9.26E-01	7.73E-01	5.35E-01	1.00E+00	1.00E+00	1.00E+00
MSH5	9.94E-01	1.00E+00	9.75E-01	7.96E-01	7.16E-01	1.00E+00	9.94E-01	8.26E-01	7.84E-01	1.00E+00	1.00E+00	1.00E+00
ND7	1.65E-13	3.43E-20	3.25E-24	1.69E-46	2.89E-11	1.62E-16	2.85E-18	7.18E-51	6.89E-01	1.00E+00	1.00E+00	1.00E+00
NOWA1	9.87E-01	1.00E+00	8.57E-01	3.23E-01	6.00E-01	1.00E+00	9.62E-01	3.13E-01	7.21E-01	1.00E+00	1.00E+00	1.00E+00
NOWA2	9.87E-01	1.00E+00	7.92E-01	3.16E-01	5.68E-01	1.00E+00	9.60E-01	2.75E-01	7.02E-01	1.00E+00	1.00E+00	1.00E+00
PDSG2	8.53E-01	1.93E-13	6.82E-01	3.83E-02	3.18E-01	1.54E-12	9.64E-01	1.07E-01	9.93E-01	1.00E+00	1.00E+00	1.00E+00
PGM	9.31E-01	1.00E+00	9.71E-01	7.76E-02	8.30E-01	1.00E+00	9.61E-01	8.70E-02	7.76E-01	1.00E+00	1.00E+00	1.00E+00
PGML1	8.64E-01	1.65E-12	8.66E-01	9.71E-03	4.68E-01	2.62E-08	9.16E-01	3.97E-02	8.99E-01	1.00E+00	1.00E+00	1.00E+00
PGML2	9.96E-01	1.46E-19	3.47E-01	1.30E-02	8.92E-01	2.71E-14	8.99E-01	5.60E-03	9.36E-01	1.00E+00	1.00E+00	1.00E+00
PGML3a	9.49E-01	1.47E-12	9.96E-02	6.76E-02	6.66E-01	4.12E-08	9.49E-01	1.78E-02	9.95E-01	1.00E+00	1.00E+00	1.00E+00
PGML3b	8.98E-01	1.11E-06	4.02E-01	3.70E-01	9.87E-01	7.64E-03	9.11E-01	1.09E-01	5.15E-01	1.00E+00	1.00E+00	1.00E+00
PGML3c	9.87E-01	4.72E-01	4.14E-02	2.65E-01	5.12E-01	5.41E-01	4.73E-01	2.46E-01	6.44E-01	1.00E+00	1.00E+00	1.00E+00
PGML4a	9.58E-01	2.15E-10	7.33E-01	2.46E-04	8.05E-01	3.26E-08	8.67E-01	3.57E-02	9.23E-01	1.00E+00	1.00E+00	1.00E+00
PGML4b	9.20E-01	2.13E-08	9.15E-01	3.77E-03	8.67E-01	5.82E-07	8.67E-01	7.23E-03	7.08E-01	1.00E+00	1.00E+00	1.00E+00
PGML5a	9.50E-01	6.89E-09	9.28E-01	5.96E-03	5.70E-01	4.48E-06	9.26E-01	2.19E-02	9.04E-01	1.00E+00	1.00E+00	1.00E+00
PGML5b	9.94E-01	2.06E-17	4.86E-01	1.25E-01	9.25E-01	2.17E-09	9.14E-01	3.09E-01	9.89E-01	1.00E+00	1.00E+00	1.00E+00
PTCAF1	9.99E-01	1.00E+00	9.79E-01	4.52E-01	6.71E-01	1.00E+00	9.60E-01	6.73E-01	6.92E-01	1.00E+00	1.00E+00	1.00E+00
PTIWI01	9.97E-01	1.00E+00	9.65E-01	5.85E-01	8.81E-01	1.00E+00	9.79E-01	7.11E-01	8.68E-01	1.00E+00	1.00E+00	1.00E+00
PTIWI09	9.96E-01	1.00E+00	9.48E-01	5.78E-01	8.72E-01	1.00E+00	9.82E-01	7.56E-01	8.43E-01	1.00E+00	1.00E+00	1.00E+00
PTIWI10	9.77E-01	1.00E+00	6.29E-19	3.04E-11	5.72E-01	1.00E+00	7.47E-06	2.73E-02	7.70E-01	1.00E+00	1.73E-01	3.46E-03
PTIWI11	9.87E-01	1.00E+00	1.33E-17	1.96E-04	9.58E-01	1.00E+00	3.54E-03	4.99E-01	9.41E-01	1.00E+00	7.50E-03	2.11E-01
SPO11	9.77E-01	1.00E+00	8.32E-01	2.05E-01	6.36E-01	1.00E+00	9.02E-01	4.19E-01	8.36E-01	1.00E+00	1.00E+00	1.00E+00
SPT5m	9.96E-01	1.00E+00	9.66E-01	4.37E-01	6.67E-01	1.00E+00	9.81E-01	6.80E-01	7.24E-01	1.00E+00	1.00E+00	1.00E+00
TFIIS4	9.87E-01	1.00E+00	9.54E-01	6.85E-01	5.42E-01	1.00E+00	9.37E-01	4.74E-01	6.76E-01	1.00E+00	1.00E+00	1.00E+00

Journal of Cell Science • Supplementary information

Table S5. Differential expression of selected genes (fold change)

Log2 transformed fold change for differential expression (thresholds: |log2(Fold Change)| > 2; adjusted p-value < 0.01) of selected genes in different knockdown comparisons and time points. Magenta = upregulation; Blue = downregulation.

	DevPF1-KD vs *ND7*-KD				*DevPF2*-KD vs *ND7*-KD				*DevPF2*-KD vs *DevPF1*-KD			
Gene	onset	early	late	very late	onset	early	late	very late	onset	early	late	very late
DCL2	0.04	-0.28	0.07	0.60	-0.61	0.27	0.08	0.04	-0.65	0.56	0.00	-0.55
DCL3	-0.07	-0.31	0.01	1.31	-0.67	0.09	0.02	0.49	-0.60	0.40	0.01	-0.82
DCL5	-0.23	-2.50	-1.15	0.82	-0.43	-2.37	-1.09	0.40	-0.20	0.12	0.07	-0.41
DevPF1	-2.94	-3.13	-1.98	-0.70	-1.44	-0.88	-0.75	-0.10	1.50	2.25	1.23	0.60
DevPF2	-0.16	-0.92	-1.38	-0.35	-0.19	-2.61	-2.74	-1.57	-0.03	-1.69	-1.36	-1.22
EZL1	-0.04	-0.01	0.29	1.02	-0.71	-0.21	0.29	0.63	-0.66	-0.20	0.00	-0.39
GTSF1	-0.17	0.03	0.31	0.96	-0.53	-0.27	0.22	0.57	-0.36	-0.29	-0.09	-0.39
ICOP1	-0.06	-3.78	-0.29	1.43	0.35	-3.05	-0.01	1.18	0.41	0.74	0.28	-0.25
ICOP2	-0.48	-3.65	-0.05	0.72	-0.61	-2.70	0.44	0.92	-0.13	0.95	0.49	0.21
ISWI1	-0.72	-1.69	-0.39	1.27	-0.08	-1.91	-0.03	1.18	0.64	-0.22	0.35	-0.09
KU80c	-0.51	-4.13	-0.93	1.13	0.38	-3.08	-0.28	1.07	0.89	1.05	0.65	-0.05
LIG4a	-0.37	0.10	-0.28	-0.23	-0.60	-0.20	-0.01	-0.01	-0.24	-0.30	0.26	0.21
MSH4a	-0.23	0.01	0.26	0.43	-0.80	-0.27	0.20	-0.05	-0.56	-0.28	-0.06	-0.47
MSH4b	-0.03	0.10	0.68	0.63	-0.62	-0.09	0.30	0.34	-0.58	-0.20	-0.38	-0.29
MSH5	-0.10	-0.24	0.10	0.41	-0.49	-0.45	0.03	0.38	-0.39	-0.21	-0.06	-0.03
ND7	1.71	2.09	2.33	3.03	1.57	1.94	2.05	2.78	-0.14	-0.15	-0.28	-0.25
NOWA1	-0.17	-0.01	-0.40	1.05	-0.62	-0.25	-0.19	1.09	-0.44	-0.24	0.21	0.04
NOWA2	-0.18	-0.20	-0.52	0.99	-0.62	-0.34	-0.19	1.07	-0.44	-0.14	0.33	0.08
PDSG2	-0.97	-4.21	-0.61	1.48	-0.96	-4.13	0.14	1.14	0.01	0.08	0.74	-0.34
PGM	-0.42	-0.15	0.06	1.11	-0.18	-0.62	0.13	1.01	0.24	-0.47	0.06	-0.10
PGML1	-0.74	-3.48	0.25	1.52	-0.60	-2.90	0.28	1.15	0.13	0.59	0.03	-0.36
PGML2	-0.04	-4.19	-0.88	1.41	-0.12	-3.63	-0.33	1.35	-0.08	0.56	0.55	-0.07
PGML3a	-0.35	-3.38	-1.23	1.12	-0.36	-2.75	-0.17	1.21	-0.01	0.62	1.06	0.10
PGML3b	1.11	-3.96	-1.10	0.83	0.03	-2.45	-0.40	1.26	-1.08	1.51	0.71	0.43
PGML3c	0.13	-1.04	-1.30	0.68	0.53	-1.07	-0.87	0.70	0.40	-0.03	0.43	0.02
PGML4a	-0.31	-3.09	0.44	1.92	-0.21	-2.80	0.43	1.13	0.10	0.29	-0.02	-0.78
PGML4b	-0.39	-2.32	0.14	1.33	-0.12	-2.15	0.35	1.10	0.27	0.17	0.21	-0.23
PGML5a	-0.36	-3.08	-0.15	1.68	-0.48	-2.60	0.26	1.31	-0.12	0.48	0.42	-0.37
PGML5b	-0.08	-4.31	-0.77	1.03	-0.09	-3.16	-0.30	0.75	-0.01	1.15	0.47	-0.28
PTCAF1	-0.02	-0.22	0.08	0.94	-0.56	0.01	0.23	0.65	-0.54	0.23	0.15	-0.29
PTIWI01	0.06	-0.15	0.20	1.09	-0.31	-0.32	0.19	0.86	-0.37	-0.17	-0.01	-0.23
PTIWI09	0.11	-0.01	0.30	1.26	-0.38	-0.33	0.18	0.85	-0.50	-0.33	-0.12	-0.41
PTIWI10	-0.23	-0.11	-5.01	-3.76	-0.54	-0.18	-2.91	-1.43	-0.30	-0.07	2.10	2.33
PTIWI11	-0.15	-0.72	-5.32	-2.61	-0.06	-0.18	-2.45	-0.73	0.09	0.54	2.87	1.88
SPO11	-0.31	-0.69	0.49	1.41	-0.59	-0.58	0.54	1.04	-0.29	0.11	0.04	-0.37
SPT5m	-0.10	-0.27	0.14	1.10	-0.65	-0.07	0.13	0.73	-0.56	0.20	-0.02	-0.37
TFIIS4	-0.17	-0.49	-0.13	0.47	-0.64	-0.58	0.29	0.79	-0.47	-0.10	0.42	0.32

Table S6. Primers used in IES Retention PCRs

IES	Primer sequence (5' to 3' orientation)
MT Locus F	GGTGTTTATATCTTAATTGTTGACCCTCAC
MT Locus R	CCATCTATACTCCATTCTTTATCTTAATTCAT
51G4404 F	CTGTTGCTACACATTGTGCATATGTTACT
51G4404 R	GCTGTAAGATTAACATTGAGCATGATCAAG

Table S7. Adapter sequences used for trimming

IES	Illumina adapter sequence (5' to 3' orientation)
DNA-seq (read 1)	AGATCGGAAGAGCACACGTCTGAACTCCAGTCA
DNA-seq (read 2)	AGATCGGAAGAGCGTCGTGTAGGGAAAGAGTGT
mRNA-seq (read 1)	AGATCGGAAGAGCACACGTCTGAACTCCAGTCAC
mRNA-seq (read 2)	AGATCGGAAGAGCGTCGTGTAGGGAAAGAGTGT
