# Supplementary material for: Two paralogous PHD finger proteins participate in natural genome editing in Paramecium tetraurelia
Source: J Cell Sci. 2024 Aug 30;137(16):jcs261979. doi: 10.1242/jcs.261979 (PMC11385659; doi:10.1242/jcs.261979)
Supplement: Supplementary file 1 [file joces-137-261979_review_history.pdf]

Two paralogous PHD finger proteins participate in natural genome editing in *Paramecium tetraurelia*

Lilia Häußermann, Aditi Singh and Estienne C. Swart
DOI: 10.1242/jcs.261979

Editor: Megan King

Review timeline
Original submission: 24 January 2024
Editorial decision: 4 March 2024
First revision received: 12 June 2024
Editorial decision: 18 June 2024
Second revision received: 26 June 2024
Accepted: 28 June 2024

Original submission

First decision letter

MS ID#: JOCES/2024/261979

MS TITLE: Two paralogous PHD finger proteins participate in Paramecium tetraurelia's natural genome editing

AUTHORS: Lilia Häußermann, Aditi Singh, and Estienne C. Swart
ARTICLE TYPE: Research Article

We have now reached a decision on the above manuscript.

To see the reviewers' reports and a copy of this decision letter, please go to: https://submit-jcs.biologists.org and click on the 'Manuscripts with Decisions' queue in the Author Area. (Corresponding author only has access to reviews.)

As you will see, the reviewers gave favourable reports but raised some important points that will require further edits to your manuscript. I hope that you will be able to carry these out because I would like to be able to accept your paper.

Please ensure that you clearly highlight all changes made in the revised manuscript. Please avoid using 'Tracked changes' in Word files as these are lost in PDF conversion.

I should be grateful if you would also provide a point-by-point response detailing how you have dealt with the points raised by the reviewers in the 'Response to Reviewers' box. Please attend to all of the reviewers' comments. If you do not agree with any of their criticisms or suggestions please explain clearly why this is so.

Reviewer 1

Advance summary and potential significance to field

Häußermann and colleagues are analyzing the mechanisms of programmed DNA elimination in the ciliate Paramecium. The general topic is of great interest, not only from an epigenetic and

© 2024. Published by The Company of Biologists under the terms of the Creative Commons Attribution License (https://creativecommons.org/licenses/by/4.0/).

transgenerational point of view, but also to understand the mechanistic of DNA elimination, which has recently become more apparent in nematodes and, surprisingly, in new cancer data. This paper characterizes novel proteins that have not been previously described to be involved. These were identified by interaction screens of known factors such as ISWI or Ptiwi proteins, and their identification therefore belongs to forward genetics and not only to the transfer of putative candidates by reverse genetics approaches.

The results of this study show two paralogous proteins with different functions and phenotypes caused by RNAi against them. DevPF1 knockdown reveals the retention of long IESs, which are those dependent on small RNAs and epigenetic modifications. This finding is consistent with the global comparison of IES retention rates with other genetic factors, which indicates that the IES retention pattern is similar to that of TFIIS4 and Dcl2,3,5, the latter being involved in sRNA accumulation and the former being a transcription elongation factor for ncRNA in the new Mac, probably necessary for transcription of the sRNA targets. Dev1 KD results in more global IES retention and also in reduced sRNA accumulation. The study therefore shows that both paralogs have different functions and are crucial for DNA elimination. The authors have carefully analyzed the PHD domain by structural analysis to support the existence of the PHD finger, so in conclusion and in the future, these proteins may be prime candidates for understanding the sRNA-chromatin interaction necessary for the DNA elimination process.
The idea that these proteins act as transcription factors is quite interesting, although complicated at first glance why using the same TFs in the meiotic Mic and the new Mac. Also, itÂ’s quite interesting that a first factor is now discovered which may contribute to the selection of meiotic products. The authors are right that it may only contribute to this as they never can identify two meiotic mic products stained, only. Maybe this could be an artefact of the overexpression in transgenic lines, leading to stronger accumulation of the GFP-tagged protein in some Mics.

I went through this manuscript several times and decided not to spend more time trying to find something to criticize. All of the open questions, some of them are mentioned above, are sufficiently discussed on the respective chapter.
The study itself is well designed, also in terms of controls. This concerns the very careful analysis and comparison of different knockdown constructs and their effects for a single target, the confirmation of the structure of the PHD domains and also the extensive work and time spent on the localization of the proteins in several cases, which indeed results in more knowledge on the dynamics of the individual components of the mechanism.

Comments for the author

no comments

<u>Reviewer 2</u>

Advance summary and potential significance to field

Review: MS ID#: JOCES/2024/261979 MS title: Two paralogous PHD finger proteins participate in Paramecium tetraurelia's natural genome editing Authors: Lilia Häußermann, Aditi Singh, and Estienne C. Swart

Summary In this study, the authors report on the identification of two paralogous PhD-domain proteins in the ciliate Paramecium and demonstrate through RNAi knockdown that they are imporant for efficient DNA elimination that occurs during the differentiation of a new somatic nucleus. They were identified as proteins that were enrich in ISWI1 immunoprecipations that showed high lethality and DNA elimination failure when knockdown during sexual development. These two paralogous proteins, named DevPF1 and DevFP2, show differential expression and localization patterns. DevPF1 was expressed in early (meiotic) development whereas DevPF2 was only expressed during postzygotic development and localized to the developing macronuclei. Knockdown of each gene resulted in some cross-over reduction in gene expression due to the high sequence similarity between the two paralogs. Knockdown of DevPF1 resulted in loss of the production of meiotically produced small RNAs important for DNA elimination. Knockdown of both genes resulted in a loss of iesRNAs, that are believed to be generated from concatenated excised

© 2024. Published by The Company of Biologists under the terms of the Creative Commons Attribution License (https://creativecommons.org/licenses/by/4.0/).

DNA. The loss of these iesRNAs could be an indirect effect cause by the decrease in the efficiency of DNA elimination. Knockdown of DevPF1 and 2 showed some effects on gene expression during development, but the expression changes were difficult to interpret due to high experimental variability, likely of the knowdown approach and phenotype caused by the knockdown.

Comments for the author

General Comments

Strengths. The key finding of this study is that the authors have identified an new protein required for production of scanRNAs, which are the ciliate equivalent of piRNAs that act in the germlines of multicellular organisms to silence transposons and package repeat sequences during development. The fact the DevPF1 is in the meiotic nuclei where scanRNAs are made is consistant with this role. Overall the experiments are well executed which together provide new insights into a likely key player involved in the control of DNA elimination in Paramecium.

Weaknesses. The inability of the authors to knockdown one or the other DevPF protein is a limitation of the study. This is particularly true for making conclusions about the function of DevPF2 as DevPF1 acts both earlier and localizes to the same location as DevPF2. Likely because of this complication, and the fact that failure to produce scanRNAs early likely affects many downstream events in genome development, limits many conclusion about whether these two proteins are doing the same of distinct actions during development. Furhtemore, the effort to look at affects of the knockdown on differential gene expression are quite variable, and thus limited conclusions can be made.

Specific comments:

1. As noted, the most convincing phenotype beyond failure of DNA elimination, ie. IES retention, is the failure of DevPF1 knockdown cells to produce scanRNAs. Given that it this also results in failure of Ptwi09 localization, it suggests that it has a role in primary scanRNA production. Did the authors try to look to see whether or not the precursor RNAs are made? This is a critical experiment to attempt as the current manuscript makes it difficult to conclude which steps in DNA elimination that DevPF1 and/or 2 act in.
2. The authors have made a potential novel discovery of the role of PhD zinc figure proteins in DNA elimination. I found the lack of information of other PhD finger proteins involved in DNA elimination an oversight. I believe the Pgm excisase has a PhD finger (Genes Dev. 2009 Nov 1; 23(21): 2478–2483). The many Pgm-like proteins may similarly have the PhD zinc finger. How does the PhD domains of DevPF1 and 2 compare to the other PhD domain proteins in Paramecium? While it is clear from Figure 1 that these proteins have the cis-his containing domain, any relationship to the human proteins is clearly speculative.
3. How do the authors know whether the localization of DevPf1 is withing the micronuclei during pre-meiotic S phase or during prophase do meiosis 1? This may be due to the reviewers lack of knowledge of Paramecium meiosis. This is potentially relevant as that timing is consistent when piRNAs form in multicellular organisms.
4. Do the authors think the that what appears to be rather random localization of DevPF1 to post meiotic gametic nuclei is significant or just the failure of it to be turned over in some cells? Did the authors try to quantify how often they see it in one type of post-meiotic germline nucleus over another? If I understand the authors description, the protein shows up on all the different types of post-meiotic micronuclei, but not is all, all of the time, which may mean that the protein is no longer having a critical function.
5. The localization shown in Figure 3B is challenging to interpret. Its not clear why the DAPI staining is shown alone, but not the GFP localization, which is much harder to see. The DAPI image would be sufficient as part of the overlay.
6. I found the discussion section overly long and lacking in narrative focus. I think part of this originates from the authors efforts to figure out what both these proteins are doing given their paralogous structure. The problem with that approach is that it has led the authors trying to describe the results related to both protiens equally, when it seems the strongest data for functionality is with DevPF1 and the lack of scnRNAs. As the differential gene expression analysis was hard to interpret, it would have made as stronger study if the authors had selected one of the two proteins to describe in more detail, what role it might have.

© 2024. Published by The Company of Biologists under the terms of the Creative Commons Attribution License (https://creativecommons.org/licenses/by/4.0/).

7. The fact that the authors felt the need to discuss many speculative roles for these two protiens stems from the fact that the manuscript cannot convincingly assign a role for either protein in DNA elimination (or other developmental process).

Minor comments:
1. Line 37-38: “Tetrahymena, IESs are predominantly imprecisely excised and only tolerated in intergenic regions…” This is not fully accurate a small number of IES in Tetrahymena are found in coding region and are precisely excised. Better to add replace “only' with “mostly” see: (G3 (Bethesda) 2011 Nov;1(6):515-22. doi: 10.1534/g3.111.000927. Epub 2011 Nov 1 and Nucleic Acids Res. 2017 Sep 19;45(16):9481-9502. doi: 10.1093/nar/gkx652.)

2. Line 41-42 “Since the coding density of the Paramecium MAC genome is high, most IESs are intragenic (Arnaiz et al., 2012).” I don't understand the logic here. The coding density of Tetrahymena is also high, but the IES are mostly intergenic. This basis for this statement is unclear.

3. line 442 : “hypothesized” is misspelled.

4. Lines 665-668. Two sentence paragraphs are generally insufficient to make strong discussion point. If one thinks this idea is important, it would be better to integrate into a more developed and focused paragraph.

First revision

Author response to reviewers' comments

Reviewer 1 Advance Summary and Potential Significance to Field:
Häußermann and colleagues are analyzing the mechanisms of programmed DNA elimination in the ciliate Paramecium. The general topic is of great interest, not only from an epigenetic and transgenerational point of view, but also to understand the mechanistic of DNA elimination, which has recently become more apparent in nematodes and, surprisingly, in new cancer data. This paper characterizes novel proteins that have not been previously described to be involved. These were identified by interaction screens of known factors such as ISWI or Ptiwi proteins, and their identification therefore belongs to forward genetics and not only to the transfer of putative candidates by reverse genetics approaches.
The results of this study show two paralogous proteins with different functions and phenotypes caused by RNAi against them. DevPF1 knockdown reveals the retention of long IESs, which are those dependent on small RNAs and epigenetic modifications. This finding is consistent with the global comparison of IES retention rates with other genetic factors, which indicates that the IES retention pattern is similar to that of TFIIS4 and Dcl2,3,5, the latter being involved in sRNA accumulation and the former being a transcription elongation factor for ncRNA in the new Mac, probably necessary for transcription of the sRNA targets. Dev1 KD results in more global IES retention and also in reduced sRNA accumulation. The study therefore shows that both paralogs have different functions and are crucial for DNA elimination. The authors have carefully analyzed the PHD domain by structural analysis to support the existence of the PHD finger, so in conclusion and in the future, these proteins may be prime candidates for understanding the sRNA- chromatin interaction necessary for the DNA elimination process.
The idea that these proteins act as transcription factors is quite interesting, although complicated at first glance why using the same TFs in the meiotic Mic and the new Mac. Also, it's quite interesting that a first factor is now discovered which may contribute to the selection of meiotic products. The authors are right that it may only contribute to this as they never can identify two meiotic mic products stained, only. Maybe this could be an artefact of the overexpression in transgenic lines, leading to stronger accumulation of the GFP-tagged protein in some Mics.
I went through this manuscript several times and decided not to spend more time trying to find something to criticize. All of the open questions, some of them are mentioned above, are sufficiently discussed on the respective chapter.
The study itself is well designed, also in terms of controls. This concerns the very careful analysis

© 2024. Published by The Company of Biologists under the terms of the Creative Commons Attribution License (https://creativecommons.org/licenses/by/4.0/).

and comparison of different knockdown constructs and their effects for a single target, the confirmation of the structure of the PHD domains and also the extensive work and time spent on the localization of the proteins in several cases, which indeed results in more knowledge on the dynamics of the individual components of the mechanism.

Reviewer 1 Comments for the Author:
no comments

We thank the reviewer for their kind words and understanding of the technical challenges we encountered with this project in determining the functions of the two paralogs individually. This is especially true for the DevPF1 protein that localizes in the MICs.

*****
Reviewer 2 Advance Summary and Potential Significance to Field:
Review: MS ID#: JOCES/2024/261979
MS title: Two paralogous PHD finger proteins participate in Paramecium tetraurelia's natural genome editing
Authors: Lilia Häußermann, Aditi Singh, and Estienne C. Swart

Summary
In this study, the authors report on the identification of two paralogous PhD- domain proteins in the ciliate Paramecium and demonstrate through RNAi knockdown that they are imporant for efficient DNA elimination that occurs during the differentiation of a new somatic nucleus. They were identified as proteins that were enrich in ISWI1 immunoprecipations that showed high lethality and DNA elimination failure when knockdown during sexual development. These two paralogous proteins, named DevPF1 and DevFP2, show differential expression and localization patterns. DevPF1 was expressed in early (meiotic) development whereas DevPF2 was only expressed during postzygotic development and localized to the developing macronuclei. Knockdown of each gene resulted in some cross-over reduction in gene expression due to the high sequence similarity between the two paralogs. Knockdown of DevPF1 resulted in loss of the production of meiotically produced small RNAs important for DNA elimination. Knockdown of both genes resulted in a loss of iesRNAs, that are believed to be generated from concatenated excised DNA. The loss of these iesRNAs could be an indirect effect cause by the decrease in the efficiency of DNA elimination. Knockdown of DevPF1 and 2 showed some effects on gene expression during development, but the expression changes were difficult to interpret due to high experimental variability, likely of the knowdown approach and phenotype caused by the knockdown.

Reviewer 2 Comments for the Author:
General Comments

Strengths. The key finding of this study is that the authors have identified an new protein required for production of scanRNAs, which are the ciliate equivalent of piRNAs that act in the germlines of multicellular organisms to silence transposons and package repeat sequences during development. The fact the DevPF1 is in the meiotic nuclei where scanRNAs are made is consistant with this role. Overall the experiments are well executed which together provide new insights into a likely key player involved in the control of DNA elimination in Paramecium.

Weaknesses. The inability of the authors to knockdown one or the other DevPF protein is a limitation of the study. This is particularly true for making conclusions about the function of DevPF2 as DevPF1 acts both earlier and localizes to the same location as DevPF2. Likely because of this complication, and the fact that failure to produce scanRNAs early likely affects many downstream events in genome development, limits many conclusion about whether these two proteins are doing the same of distinct actions during development. Furhtemore, the effort to look at affects of the knockdown on differential gene expression are quite variable, and thus limited conclusions can be made.

We appreciate the comments. As we tried to carefully explain and analyze, it simply is not currently possible to knock down such close paralogs in Paramecium without some cross-silencing.

© 2024. Published by The Company of Biologists under the terms of the Creative Commons Attribution License (https://creativecommons.org/licenses/by/4.0/).

Nevertheless, we did report differences with silencing constructs targeting one gene vs. the other (esp. the sRNAs in Fig 6A), consistent with each gene being affected more by its silencing construct. We agree that it is difficult to conclude the exact functions of DevPF proteins based on the results presented, including their similarity at the nucleotide and amino acid levels, and similar localization during the late stages of the sexual reproduction. This is why we have chosen not to assign very specific roles for the proteins and rather focussed on the possible explanations for the observations.

Specific comments:

1. As noted, the most convincing phenotype beyond failure of DNA elimination, ie. IES retention, is the failure of DevPF1 knockdown cells to produce scanRNAs. Given that it this also results in failure of Ptwi09 localization, it suggests that it has a role in primary scanRNA production. Did the authors try to look to see whether or not the precursor RNAs are made? This is a critical experiment to attempt as the current manuscript makes it difficult to conclude which steps in DNA elimination that DevPF1 and/or 2 act in.

There are three kinds of precursor RNAs of scnRNAs: (i) long ssRNAs which become (ii) long dsRNAs and are further processed into (iii) short dsRNA products of Dcl2/3 cleavage of the long dsRNAs. As far as we are aware, no researchers have sequenced any of these precursors or attempted to analyze them by other means.

It is not known what properties distinguish the long ssRNA precursors from mRNAs. In principle, long dsRNAs could be distinguished from mRNAs, but, without a specific distinguishing feature, could not be disentangled from long dsRNAs produced by antisense transcription by RNA-dependent RNA Polymerases (RdRPs) from mRNAs (necessary for siRNA production). Furthermore, it has been postulated that the long RNAs are transcribed from MICs early during meiosis or at the onset of autogamy, but the exact timing is unknown. Analyzing the long RNA precursors would thus be difficult without additional knowledge about them. We believe this topic warrants an entire research project/paper of its own (we wrote: “Future investigations of bi-directional transcription and scnRNA biogenesis will allow identification of how these molecules cooperate.” - lines 434-435).

As far as the short dsRNA precursors are concerned we did not observe much difference in the expression of these genes with or without DevPF1-KD (Figure 7D). We did not investigate Dcl2 or 3 localization, so there is a possibility that their localization to meiotic MICs was inhibited by DevPF1-KD. Nevertheless, the consequences of this would be expected to be similar to that of failed Ptiwi01/09 localization: failure to produce the mature single- stranded scnRNAs. We would thus argue that the information we obtained from the sequenced scnRNAs is sufficient at present.

2. The authors have made a potential novel discovery of the role of PhD zinc figure proteins in DNA elimination. I found the lack of information of other PhD finger proteins involved in DNA elimination an oversight. I believe the Pgm excisase has a PhD finger (Genes Dev. 2009 Nov 1; 23(21): 2478–2483). The many Pgm-like proteins may similarly have the PhD zinc finger. How does the PhD domains of DevPF1 and 2 compare to the other PhD domain proteins in Paramecium? While it is clear from Figure 1 that these proteins have the cis- his containing domain, any relationship to the human proteins is clearly speculative.

We are unsure what is being suggested about the inclusion of human proteins. We included human and Drosophila homologs for the purposes of multiple sequence alignments and structural biology. Whether the Paramecium proteins have a common ancestor with these or evolved in parallel would, of course, be a question we have not attempted to address.

There are hundreds of PHD or PHD-like zinc finger domain-containing proteins in the *Paramecium* MAC genome, including Pgm and Pgmls (e.g. 913 with the following query: https://www.uniprot.org/uniprotkb?query=%28organism_id%3A5888%29+AN D+PHD). PHD finger proteins in *Paramecium* fall into a very diverse range of proteins. We avoided making comparisons to other *Paramecium* PHD finger proteins as they have not been studied in *Paramecium*, and their functions remain unknown.

© 2024. Published by The Company of Biologists under the terms of the Creative Commons Attribution License (https://creativecommons.org/licenses/by/4.0/).

It has been reported that the Pgm does not have a canonical PHD domain structure, but rather a C1 cross-brace zinc finger motif (doi.org/10.1186/s13100-021-00240-4 - “[PGM] adopts a PHD-like cross- brace zinc finger fold (i.e. a zinc finger, in which the structural cores of the two zinc ions overlap)”; see also: https://doi.org/10.1093/nar/gky044). On the other hand, our multiple sequence alignments and the Alpha Fold predictions strongly point towards a highly conserved PHD domain with different structures in DevPFs. We have added a sentence to the manuscript (current lines 140-141), to describe this. If there is any relation between the DevPF PHD zinc fingers and those of PGM or the PGM-likes, we presume it would be due to ancient common ancestry, potentially prior to the origin of PGMs.

3. How do the authors know whether the localization of DevPf1 is withing the micronuclei during pre-meiotic S phase or during prophase do meiosis 1? This may be due to the reviewers lack of knowledge of Paramecium meiosis. This is potentially relevant as that timing is consistent when piRNAs form in multicellular organisms.

The pre-meiotic S-phase is when the MICs in starved cells are swollen (https://doi.org/10.1016/0014-4827(86)90492-1) and look relatively large compared to vegetative MICs. The MICs then form a crescent shape that supposedly marks the prophase of meiosis I. The pre-meiotic S-phase stage is shown in Fig 2A labelled “S-phase” and DevPF1-GFP localization is visible within the swollen MICs.

4. Do the authors think the that what appears to be rather random localization of DevPF1 to post meiotic gametic nuclei is significant or just the failure of it to be turned over in some cells? Did the authors try to quantify how often they see it in one type of post-meiotic germline nucleus over another? If I understand the authors description, the protein shows up on all the different types of post-meiotic micronuclei, but not is all, all of the time, which may mean that the protein is no longer having a critical function.

We rather think the localization is significant. If the localization of DevPF1- GFP were a result of failed turnover in some cells, this random behavior would be asynchronous over a very long time range and over several nuclear stages (gametic and zygotic nuclei). We think this is unlikely, as protein localization is commonly quite tightly regulated. We cannot exclude that DevPF1-GFP has no critical function in these nuclei. However, the vast majority of proteins localizing to a sub-cellular compartment do have a function there. A scenario where a protein is randomly imported to some nuclei while it is excluded from others is much harder to explain than if it is specifically imported to a location where it carries out a function.

As the reviewer has pointed out in question 5, the GFP localization is hard to see, making quantification challenging. Quantification of how often DevPF1- GFP appears in different types of germline nuclei in a sufficiently high number of cells is difficult as it requires a lot of imaging time and post-processing analysis to identify DevPF1-GFP-positive cells. The signal is only visible in individual z-planes after a careful comparison of the GFP- and DAPI channels (see Fig 3A). Nevertheless, we have seen the localization in multiple sets of experiments, so we are confident that this is not a random artefact. The signal is overwhelmed in maximum intensity projections (see Fig 2A).

5. The localization shown in Figure 3B is challenging to interpret. Its not clear why the DAPI staining is shown alone, but not the GFP localization, which is much harder to see. The DAPI image would be sufficient as part of the overlay.

In Fig 3B, for each image, maximum intensity projections (upper part) and individual z-planes (lower part) are shown. Due to space constraints within the figure, not all channels were shown for the maximum intensity projection and the z-planes. The DAPI channel is shown alone for the maximum intensity projection to show the positions of all the nuclei. It was not shown again for the z-planes, as the DAPI is nicely visible in the overlays. As shown in Fig 2A, the DevPF1-GFP localization to MICs is not visible in maximum intensity projections. To save space, the GFP channel has not been included for the maximum intensity projections. It is only shown for the individual z-planes, as the important information can be extracted there. Also, the tubulin localization is shown alone for the individual z-planes, as the aim of this figure is to investigate the dependence of DevPF1-GFP localization on nuclear division (visualized by the spindle

© 2024. Published by The Company of Biologists under the terms of the Creative Commons Attribution License (https://creativecommons.org/licenses/by/4.0/).

apparatus). Including the DAPI channel would necessitate decreasing the size of each picture, which would further reduce visibility.

6. I found the discussion section overly long and lacking in narrative focus. I think part of this originates from the authors efforts to figure out what both these proteins are doing given their paralogous structure. The problem with that approach is that it has led the authors trying to describe the results related to both protiens equally, when it seems the strongest data for functionality is with DevPF1 and the lack of scnRNAs. As the differential gene expression analysis was hard to interpret, it would have made as stronger study if the authors had selected one of the two proteins to describe in more detail, what role it might have.

We double-checked Discussion to ensure that it contained no superfluous text. Minus References, Discussion is about 24% of the main manuscript's word count, which does not seem excessive. The Discussion is structured as follows: (i) Consideration of the DevPF PHD domains and interactions in the context of what is known in other organisms; (ii) Consideration of whether the DevPF's may have a role as transcription factors; (iii) Consideration of the unusual nuclear localization pattern of DevPF1; (iv) Conclusion. About half of this considers the possible roles of the DevPFs. We think this is not unreasonable. The fact that there is a dedicated discussion section about the DevPF1 localization demonstrates that this protein was considered in more detail. Certainly, we did not seek to balance the discussion of the two proteins. We also feel that it is honest and better serves possible readers to discuss not just the strongest results, but also that which is uncertain and needs to be resolved.

Furthermore, these proteins were not described together solely because they are paralogs, but rather because it is quite common in *Paramecium* for such paralogs to arise due to a whole genome duplication. It is always of interest to characterize all the related paralogs, since it's possible that they may have functionally diverged since the genome duplication. There are many examples of paralogous genes exhibiting similar functions (e. g. PTIWI01/09, DCL2/3, ICOP1/2) or different functions (DCL2/3 vs. DCL5, ISWI1 vs other ISIWI paralogs). Splitting up the stories and only focusing on one paralog would have led to other researchers wondering what is known about the other paralog (we've had reviewers objecting to not characterizing all the WGD paralogs of particular proteins in the past, though it is not always practical when there are too many of them). Although DevPF2 knockdown shows no loss of scnRNA production, it has many biologically relevant effects on IES excision, including IES retention and loss of iesRNA production. This is also of interest to other researchers and essential to present in the context of DevPF1.

7. The fact that the authors felt the need to discuss many speculative roles for these two protiens stems from the fact that the manuscript cannot convincingly assign a role for either protein in DNA elimination (or other developmental process).

It was not the manuscript's intention to ascribe precise roles to these proteins beyond their necessity for new MAC genome development. This simply was not possible within the time frame of one PhD student's studies, though a range of analyses was performed to gain as much understanding as possible. We present significant data in identifying the precise role of these and other proteins in future. We do not hide that additional research is still needed to understand the functions. Hence, we presented our key findings without making unsubstantiated claims and impartially discussed our subsequent considerations.

Minor comments:
1. Line 37-38: "Tetrahymena, IESs are predominantly imprecisely excised and only tolerated in intergenic regions…" This is not fully accurate a small number of IES in Tetrahymena are found in coding region and are precisely excised. Better to add replace "only' with "mostly" see: (G3 (Bethesda) 2011 Nov;1(6):515-22. doi: 10.1534/g3.111.000927. Epub 2011 Nov 1 and Nucleic Acids Res. 2017 Sep 19;45(16):9481-9502. doi: 10.1093/nar/gkx652.)

Sorry for the error, we meant "mostly". We have corrected this in the manuscript.

2. Line 41-42 "Since the coding density of the Paramecium MAC genome is high, most IESs are intragenic (Arnaiz et al., 2012)." I don't understand the logic here. The coding density of Tetrahymena is also high, but the IES are mostly intergenic. This basis for this statement is

© 2024. Published by The Company of Biologists under the terms of the Creative Commons Attribution License (https://creativecommons.org/licenses/by/4.0/).

unclear.

Apologies about this, part of the logic was indeed missing. We changed this sentence to: The coding density of the Paramecium MAC genome is high and there is only a weak bias towards IESs being located in intergenic regions, so most IESs are intragenic (Arnaiz et al., 2012).

3. line 442 : "hypothesized" is misspelled.

Corrected.

4. Lines 665-668. Two sentence paragraphs are generally insufficient to make strong discussion point. If one thinks this idea is important, it would be better to integrate into a more developed and focused paragraph.

We believe the line numbers mentioned are incorrect; the reviewers meant 465-468. We have merged these sentences with the paragraph above where it fits better.

Second decision letter

MS ID#: JOCES/2024/261979

MS TITLE: Two paralogous PHD finger proteins participate in Paramecium tetraurelia's natural genome editing

AUTHORS: Lilia Häußermann, Aditi Singh, and Estienne C. Swart
ARTICLE TYPE: Research Article

Thank you for your recent resubmission. I would like to be able to accept your paper without returning it to the reviewers. However, I would ask that you consider one point made by Reviewer 2 - namely that you clarify in the text if there have been/have not been any prior studies linking PhD finger proteins to DNA elimination in Paramecium or indeed any organism. Even if many related proteins have not been studied to date, it would still be helpful to the reader to understand the current state of the field.

The specific point was the following:
2. The authors have made a potential novel discovery of the role of PhD zinc figure proteins in DNA elimination. I found the lack of information of other PhD finger proteins involved in DNA elimination an oversight.

Again, perhaps there is no literature to cite on this point, but even if not it would be helpful if there was a clear statement to that effect, which at least I could not discern from the manuscript or the response to the reviewer.

A short response detailing how you have addressed the point above in a cover letter will suffice. Thank you.

Second revision

Author response to reviewers' comments

Dear Dr. King,

Thank you for your time and for considering our manuscript " Two paralogous PHD finger proteins participate in Paramecium tetraurelia's natural genome editing" (MS ID#: JOCES/2024/261979) for publication.

© 2024. Published by The Company of Biologists under the terms of the Creative Commons Attribution License (https://creativecommons.org/licenses/by/4.0/).

We apologize for the delay in replying: one of us has been travelling and both of us were ill this weekend. We also needed a little more time to look into your request.

As per your suggestion and that of Reviewer 2, we again reviewed the literature to find other PHD finger proteins that might have a suggested role in DNA elimination. As we explain, one paper misclassified a PHD finger in a different protein in Tetrahymena involved in DNA elimination. We could find only one other relevant protein, which was described in passing in a paper reporting a specialized condensin complex involved in Tetrahymena DNA elimination but is orthologous to the proteins we report. We did not find enrichment in condensin protein peptides in our Paramecium PHD finger protein IPs. So, for the moment, the relationship between the DevPFs and condensin complexes remains an open question. The revisions are highlighted and included in lines 143-158 in Results and 416-427 in Discussion.

We are confident that the revisions we have made in response to the reviewers' comments have now effectively addressed all their main concerns. We look forward to the possibility of our manuscript now being accepted for publication.

We would also kindly request that JCS includes Dr. Aditi Singh as a co-corresponding author, as we were unable to find a way to indicate this in the submission system.

Kind regards,

Dr. Estienne C. Swart and Dr. Aditi Singh

<u>Third decision letter</u>

MS ID#: JOCES/2024/261979

MS TITLE: Two paralogous PHD finger proteins participate in Paramecium tetraurelia's natural genome editing

AUTHORS: Lilia Häußermann, Aditi Singh, and Estienne C. Swart
ARTICLE TYPE: Research Article

I am happy to tell you that your manuscript has been accepted for publication in Journal of Cell Science, pending standard publication integrity checks.

© 2024. Published by The Company of Biologists under the terms of the Creative Commons Attribution License (https://creativecommons.org/licenses/by/4.0/).
